# Supplementary material for: Connexin43 promotes exocytosis of damaged lysosomes through actin remodelling
Source: EMBO J. 2024 Jul 23;43(17):3627–49. doi: 10.1038/s44318-024-00177-3 (PMC11377567; doi:10.1038/s44318-024-00177-3)
Supplement: Supplementary file 1 — Appendix [file 44318_2024_177_MOESM1_ESM.pptx]

## Slide 1
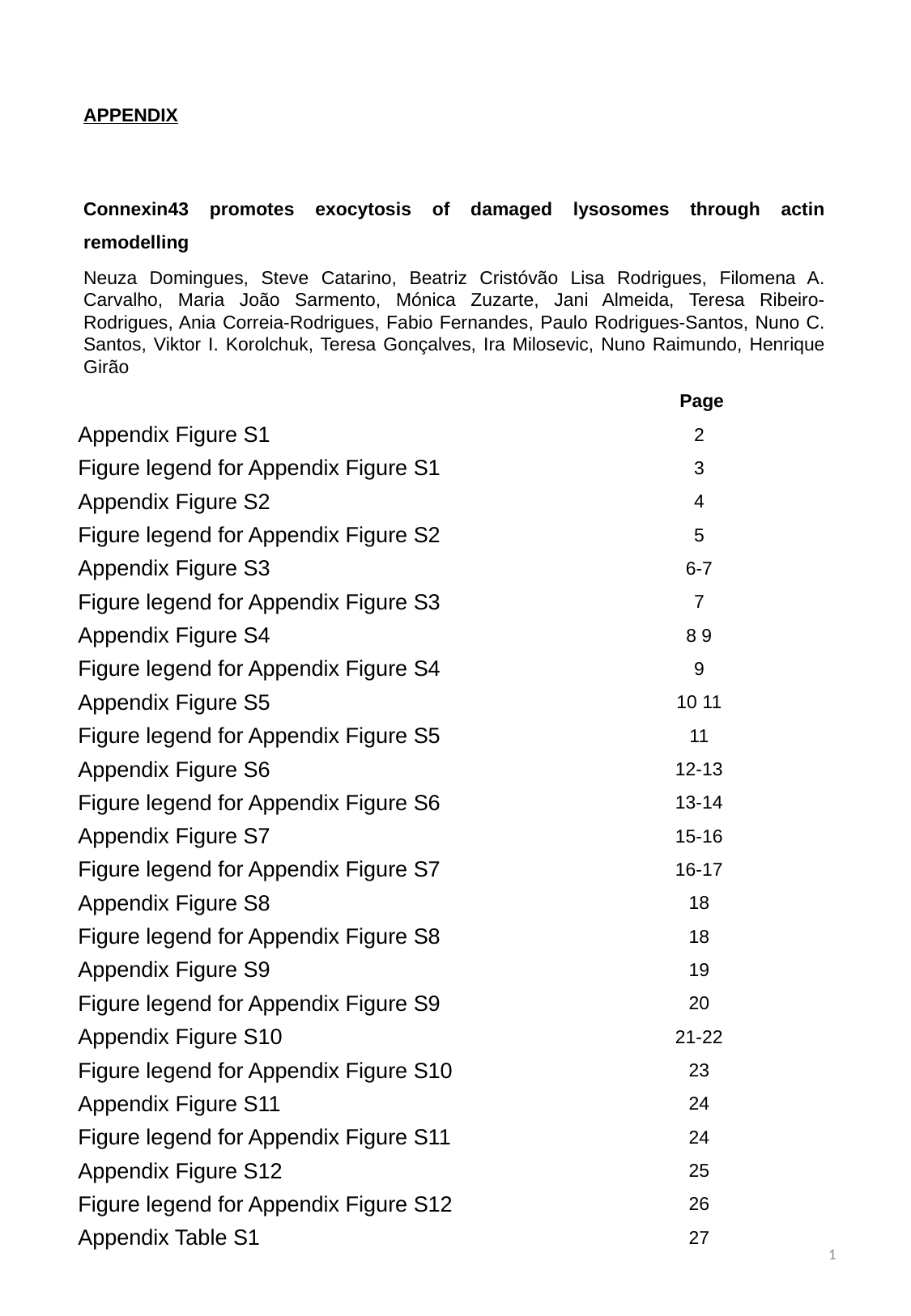

APPENDIX
Connexin43 promotes exocytosis of damaged lysosomes through actin remodelling
Neuza Domingues, Steve Catarino, Beatriz Cristóvão Lisa Rodrigues, Filomena A. Carvalho, Maria João Sarmento, Mónica Zuzarte, Jani Almeida, Teresa Ribeiro-Rodrigues, Ania Correia-Rodrigues, Fabio Fernandes, Paulo Rodrigues-Santos, Nuno C. Santos, Viktor I. Korolchuk, Teresa Gonçalves, Ira Milosevic, Nuno Raimundo, Henrique Girão
| | Page |
| --- | --- |
| Appendix Figure S1 | 2 |
| Figure legend for Appendix Figure S1 | 3 |
| Appendix Figure S2 | 4 |
| Figure legend for Appendix Figure S2 | 5 |
| Appendix Figure S3 | 6-7 |
| Figure legend for Appendix Figure S3 | 7 |
| Appendix Figure S4 | 8 9 |
| Figure legend for Appendix Figure S4 | 9 |
| Appendix Figure S5 | 10 11 |
| Figure legend for Appendix Figure S5 | 11 |
| Appendix Figure S6 | 12-13 |
| Figure legend for Appendix Figure S6 | 13-14 |
| Appendix Figure S7 | 15-16 |
| Figure legend for Appendix Figure S7 | 16-17 |
| Appendix Figure S8 | 18 |
| Figure legend for Appendix Figure S8 | 18 |
| Appendix Figure S9 | 19 |
| Figure legend for Appendix Figure S9 | 20 |
| Appendix Figure S10 | 21-22 |
| Figure legend for Appendix Figure S10 | 23 |
| Appendix Figure S11 | 24 |
| Figure legend for Appendix Figure S11 | 24 |
| Appendix Figure S12 | 25 |
| Figure legend for Appendix Figure S12 | 26 |
| Appendix Table S1 | 27 |
1

## Slide 2
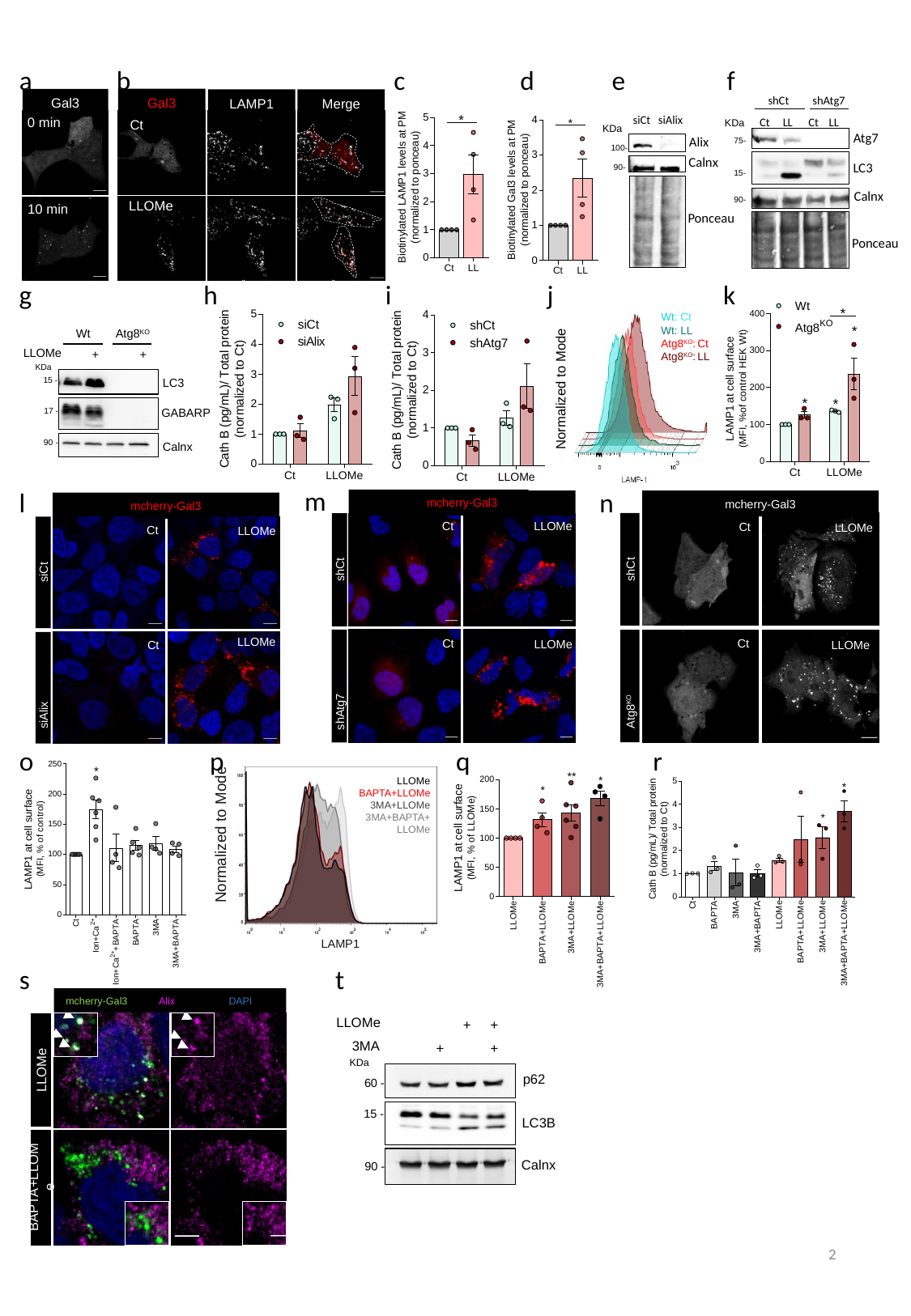

a
b
c
d
e
f
shCt shAtg7
 Ct LL Ct LL
Atg7
LC3
Calnx
Ponceau
KDa
75-
15-
90-
Gal3
0 min
10 min
Gal3
LAMP1
Merge
Ct
LLOMe
siCt siAlix
Alix
Ponceau
Calnx
KDa
100-
90-
g
h
i
j
k
Normalized to Mode
Wt: Ct
Wt: LL
Atg8KO: Ct
Atg8KO: LL
Atg8KO
Wt
LLOMe
+
+
KDa
15 -
LC3
90 -
Calnx
GABARP
17 -
m
l
n
mcherry-Gal3
shCt
shAtg7
Ct
LLOMe
Ct
LLOMe
mcherry-Gal3
shCt
Ct
LLOMe
Atg8KO
Ct
LLOMe
mcherry-Gal3
siCt
siAlix
Ct
LLOMe
LLOMe
Ct
o
p
q
r
LLOMe
BAPTA+LLOMe
3MA+LLOMe
3MA+BAPTA+
LLOMe
Normalized to Mode
LAMP1
s
t
mcherry-Gal3 Alix DAPI
LLOMe
BAPTA+LLOMe
LLOMe
+
+
3MA
+
+
KDa
p62
60 -
15 -
LC3B
Calnx
90 -
2

## Slide 3
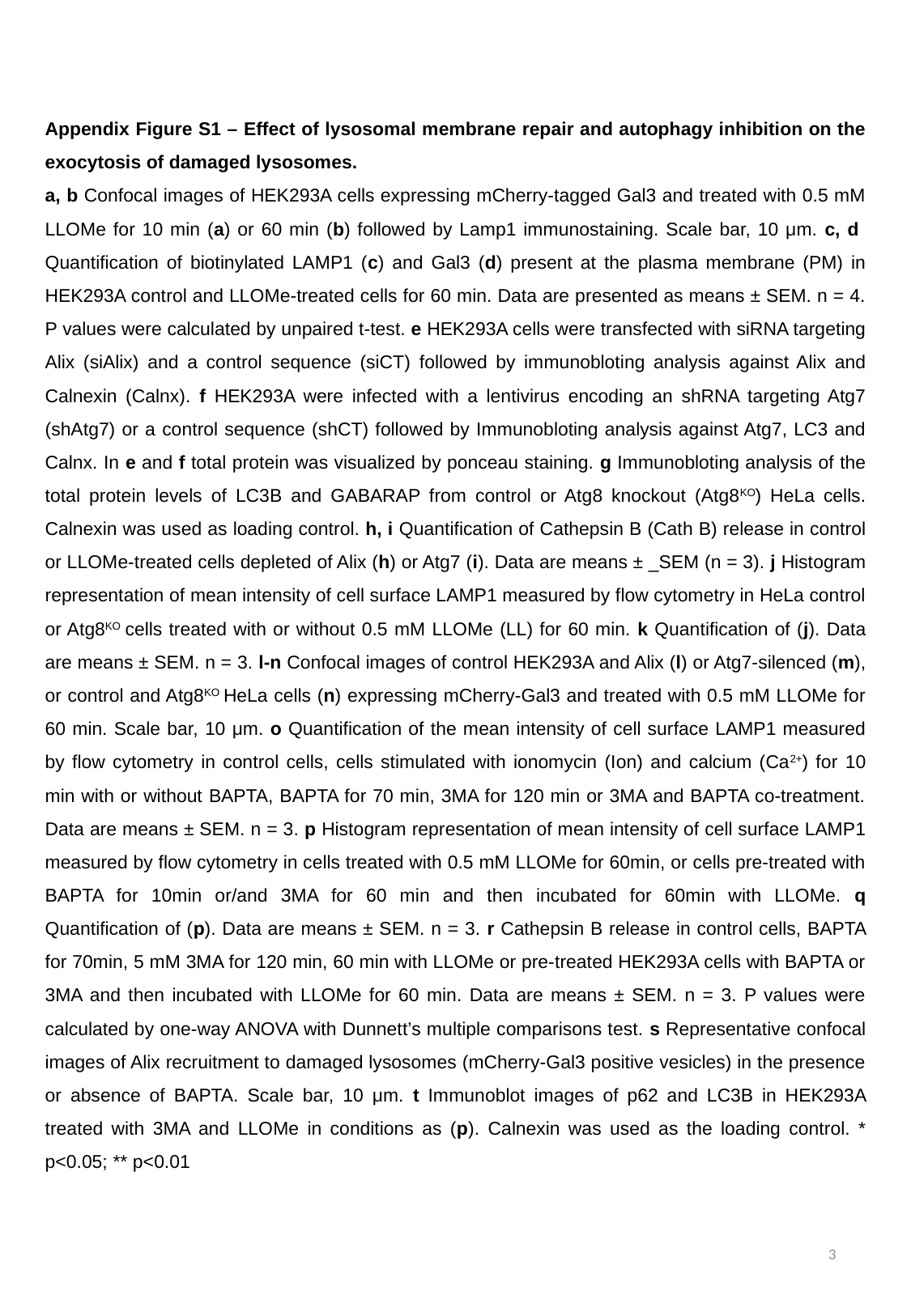

Appendix Figure S1 – Effect of lysosomal membrane repair and autophagy inhibition on the exocytosis of damaged lysosomes.
a, b Confocal images of HEK293A cells expressing mCherry-tagged Gal3 and treated with 0.5 mM LLOMe for 10 min (a) or 60 min (b) followed by Lamp1 immunostaining. Scale bar, 10 μm. c, d Quantification of biotinylated LAMP1 (c) and Gal3 (d) present at the plasma membrane (PM) in HEK293A control and LLOMe-treated cells for 60 min. Data are presented as means ± SEM. n = 4. P values were calculated by unpaired t-test. e HEK293A cells were transfected with siRNA targeting Alix (siAlix) and a control sequence (siCT) followed by immunobloting analysis against Alix and Calnexin (Calnx). f HEK293A were infected with a lentivirus encoding an shRNA targeting Atg7 (shAtg7) or a control sequence (shCT) followed by Immunobloting analysis against Atg7, LC3 and Calnx. In e and f total protein was visualized by ponceau staining. g Immunobloting analysis of the total protein levels of LC3B and GABARAP from control or Atg8 knockout (Atg8KO) HeLa cells. Calnexin was used as loading control. h, i Quantification of Cathepsin B (Cath B) release in control or LLOMe-treated cells depleted of Alix (h) or Atg7 (i). Data are means ± _SEM (n = 3). j Histogram representation of mean intensity of cell surface LAMP1 measured by flow cytometry in HeLa control or Atg8KO cells treated with or without 0.5 mM LLOMe (LL) for 60 min. k Quantification of (j). Data are means ± SEM. n = 3. l-n Confocal images of control HEK293A and Alix (l) or Atg7-silenced (m), or control and Atg8KO HeLa cells (n) expressing mCherry-Gal3 and treated with 0.5 mM LLOMe for 60 min. Scale bar, 10 μm. o Quantification of the mean intensity of cell surface LAMP1 measured by flow cytometry in control cells, cells stimulated with ionomycin (Ion) and calcium (Ca2+) for 10 min with or without BAPTA, BAPTA for 70 min, 3MA for 120 min or 3MA and BAPTA co-treatment. Data are means ± SEM. n = 3. p Histogram representation of mean intensity of cell surface LAMP1 measured by flow cytometry in cells treated with 0.5 mM LLOMe for 60min, or cells pre-treated with BAPTA for 10min or/and 3MA for 60 min and then incubated for 60min with LLOMe. q Quantification of (p). Data are means ± SEM. n = 3. r Cathepsin B release in control cells, BAPTA for 70min, 5 mM 3MA for 120 min, 60 min with LLOMe or pre-treated HEK293A cells with BAPTA or 3MA and then incubated with LLOMe for 60 min. Data are means ± SEM. n = 3. P values were calculated by one-way ANOVA with Dunnett’s multiple comparisons test. s Representative confocal images of Alix recruitment to damaged lysosomes (mCherry-Gal3 positive vesicles) in the presence or absence of BAPTA. Scale bar, 10 μm. t Immunoblot images of p62 and LC3B in HEK293A treated with 3MA and LLOMe in conditions as (p). Calnexin was used as the loading control. * p<0.05; ** p<0.01
3

## Slide 4
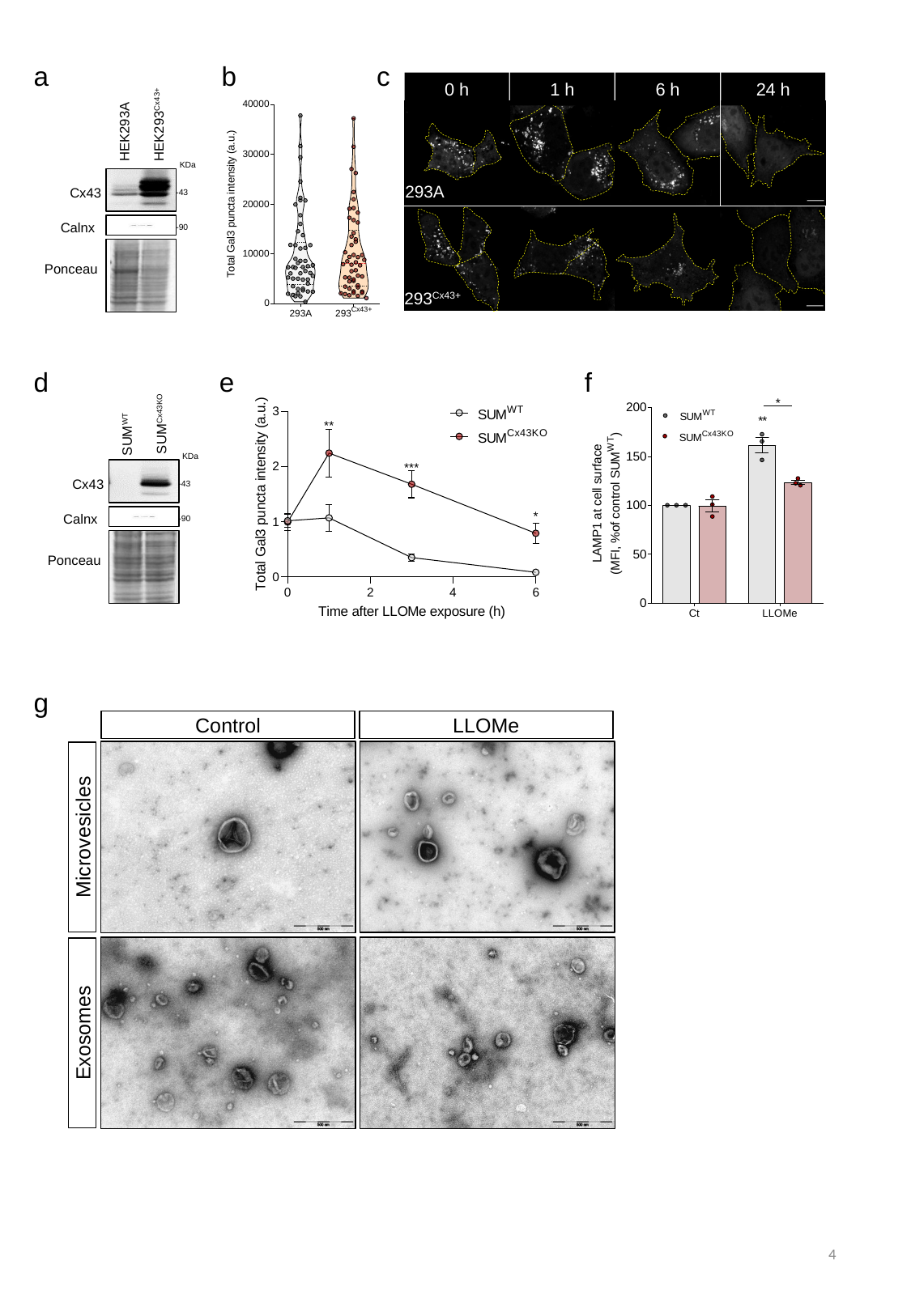

a
b
c
HEK293Cx43+
HEK293A
KDa
Cx43
-43
Calnx
-90
Ponceau
0 h
1 h
6 h
24 h
293A
293Cx43+
d
e
f
SUMCx43KO
SUMWT
KDa
Cx43
-43
Calnx
-90
Ponceau
g
Control
LLOMe
Microvesicles
Exosomes
4

## Slide 5
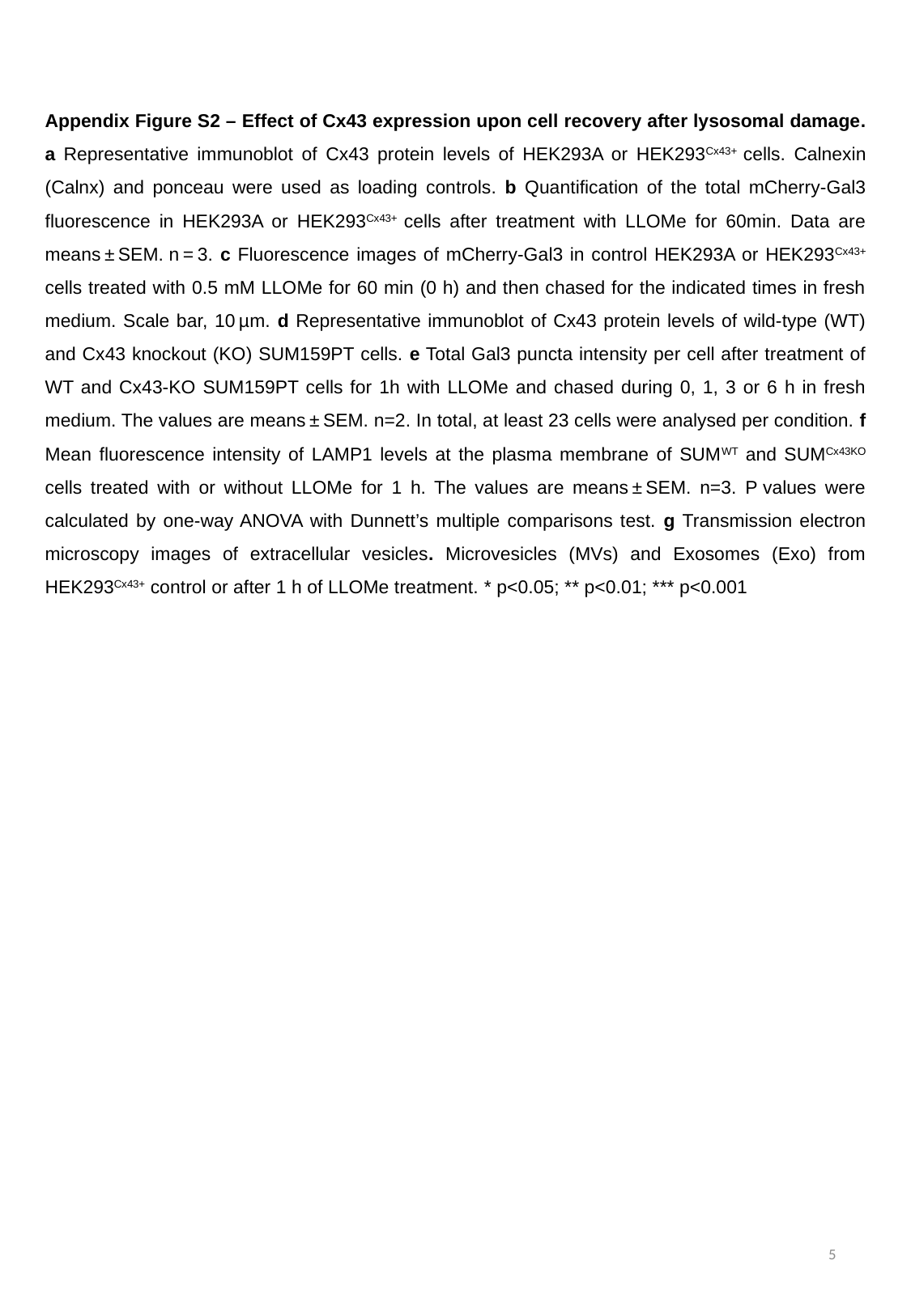

Appendix Figure S2 – Effect of Cx43 expression upon cell recovery after lysosomal damage. a Representative immunoblot of Cx43 protein levels of HEK293A or HEK293Cx43+ cells. Calnexin (Calnx) and ponceau were used as loading controls. b Quantification of the total mCherry-Gal3 fluorescence in HEK293A or HEK293Cx43+ cells after treatment with LLOMe for 60min. Data are means ± SEM. n = 3. c Fluorescence images of mCherry-Gal3 in control HEK293A or HEK293Cx43+ cells treated with 0.5 mM LLOMe for 60 min (0 h) and then chased for the indicated times in fresh medium. Scale bar, 10 µm. d Representative immunoblot of Cx43 protein levels of wild-type (WT) and Cx43 knockout (KO) SUM159PT cells. e Total Gal3 puncta intensity per cell after treatment of WT and Cx43-KO SUM159PT cells for 1h with LLOMe and chased during 0, 1, 3 or 6 h in fresh medium. The values are means ± SEM. n=2. In total, at least 23 cells were analysed per condition. f Mean fluorescence intensity of LAMP1 levels at the plasma membrane of SUMWT and SUMCx43KO cells treated with or without LLOMe for 1 h. The values are means ± SEM. n=3. P values were calculated by one-way ANOVA with Dunnett’s multiple comparisons test. g Transmission electron microscopy images of extracellular vesicles. Microvesicles (MVs) and Exosomes (Exo) from HEK293Cx43+ control or after 1 h of LLOMe treatment. * p<0.05; ** p<0.01; *** p<0.001
5

## Slide 6
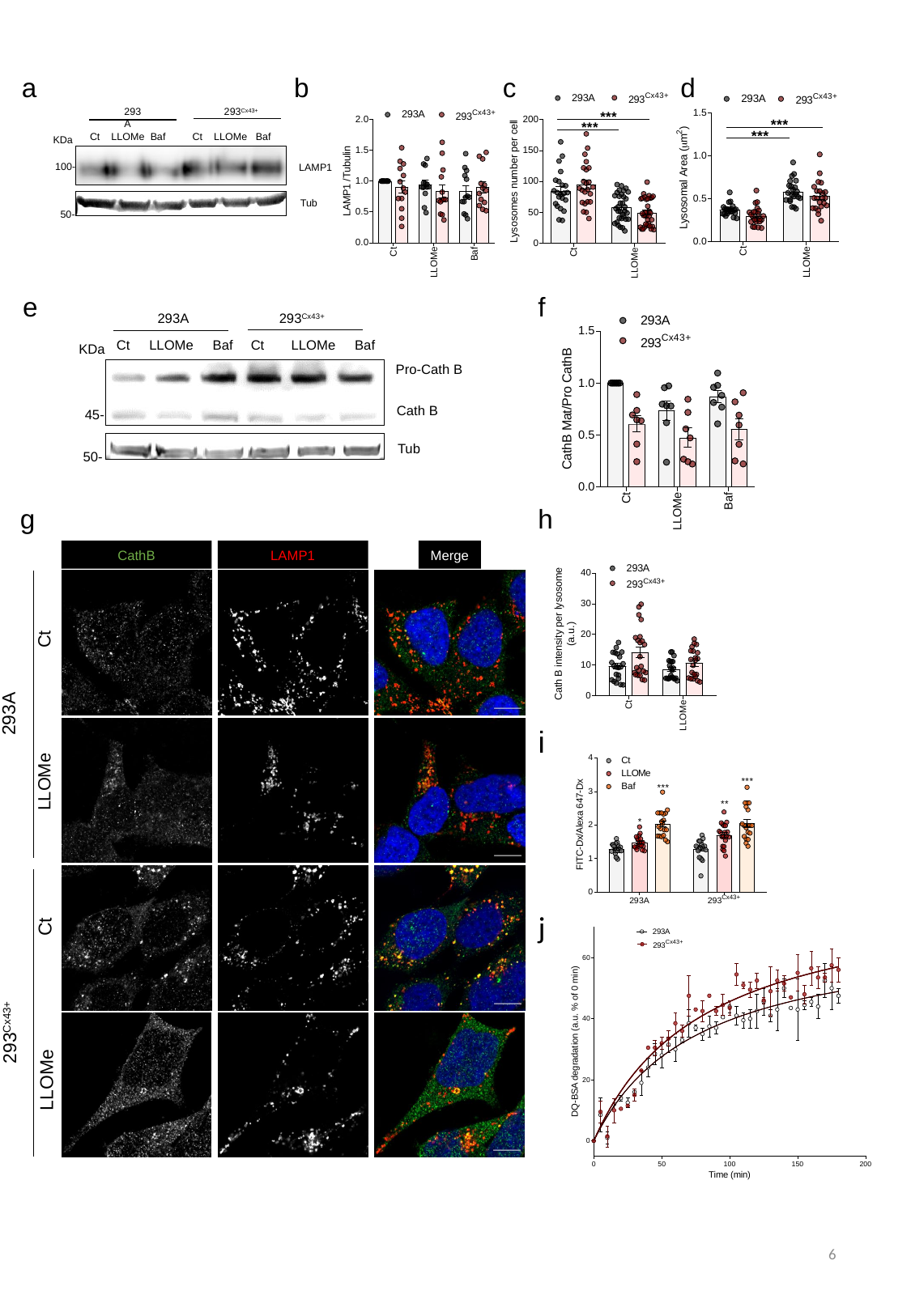

a
b
c
d
293A
293Cx43+
Ct LLOMe Baf
Ct LLOMe Baf
LAMP1
Tub
KDa
100-
50-
e
f
293A
293Cx43+
Ct LLOMe Baf
Ct LLOMe Baf
Pro-Cath B
Cath B
Tub
KDa
45-
50-
h
g
CathB
LAMP1
Merge
Ct
293A
LLOMe
Ct
293Cx43+
LLOMe
i
j
6

## Slide 7
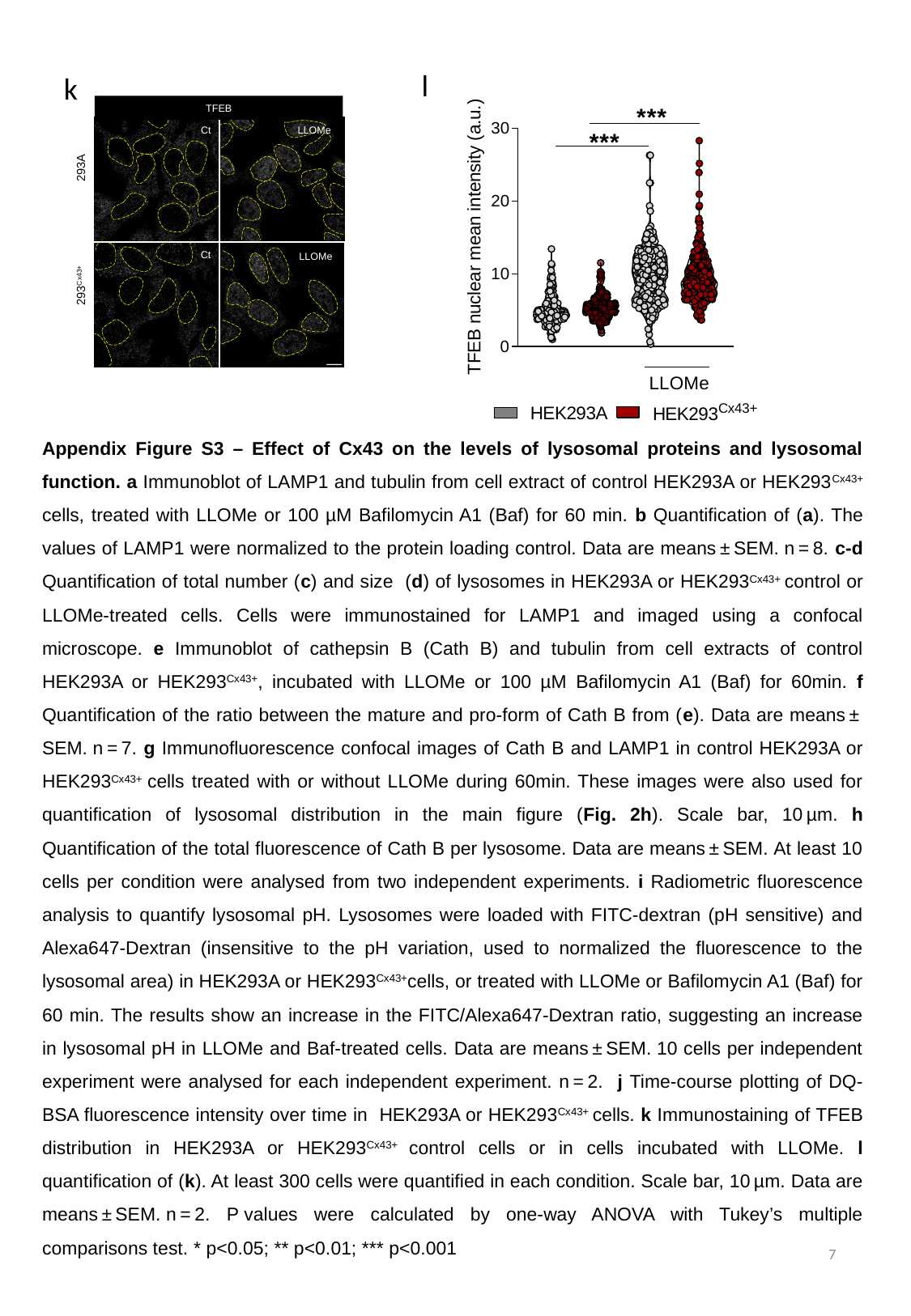

l
k
TFEB
LLOMe
Ct
293A
Ct
LLOMe
293Cx43+
Appendix Figure S3 – Effect of Cx43 on the levels of lysosomal proteins and lysosomal function. a Immunoblot of LAMP1 and tubulin from cell extract of control HEK293A or HEK293Cx43+ cells, treated with LLOMe or 100 µM Bafilomycin A1 (Baf) for 60 min. b Quantification of (a). The values of LAMP1 were normalized to the protein loading control. Data are means ± SEM. n = 8. c-d Quantification of total number (c) and size (d) of lysosomes in HEK293A or HEK293Cx43+ control or LLOMe-treated cells. Cells were immunostained for LAMP1 and imaged using a confocal microscope. e Immunoblot of cathepsin B (Cath B) and tubulin from cell extracts of control HEK293A or HEK293Cx43+, incubated with LLOMe or 100 µM Bafilomycin A1 (Baf) for 60min. f Quantification of the ratio between the mature and pro-form of Cath B from (e). Data are means ± SEM. n = 7. g Immunofluorescence confocal images of Cath B and LAMP1 in control HEK293A or HEK293Cx43+ cells treated with or without LLOMe during 60min. These images were also used for quantification of lysosomal distribution in the main figure (Fig. 2h). Scale bar, 10 µm. h Quantification of the total fluorescence of Cath B per lysosome. Data are means ± SEM. At least 10 cells per condition were analysed from two independent experiments. i Radiometric fluorescence analysis to quantify lysosomal pH. Lysosomes were loaded with FITC-dextran (pH sensitive) and Alexa647-Dextran (insensitive to the pH variation, used to normalized the fluorescence to the lysosomal area) in HEK293A or HEK293Cx43+cells, or treated with LLOMe or Bafilomycin A1 (Baf) for 60 min. The results show an increase in the FITC/Alexa647-Dextran ratio, suggesting an increase in lysosomal pH in LLOMe and Baf-treated cells. Data are means ± SEM. 10 cells per independent experiment were analysed for each independent experiment. n = 2. j Time-course plotting of DQ-BSA fluorescence intensity over time in HEK293A or HEK293Cx43+ cells. k Immunostaining of TFEB distribution in HEK293A or HEK293Cx43+ control cells or in cells incubated with LLOMe. l quantification of (k). At least 300 cells were quantified in each condition. Scale bar, 10 µm. Data are means ± SEM. n = 2. P values were calculated by one-way ANOVA with Tukey’s multiple comparisons test. * p<0.05; ** p<0.01; *** p<0.001
7

## Slide 8
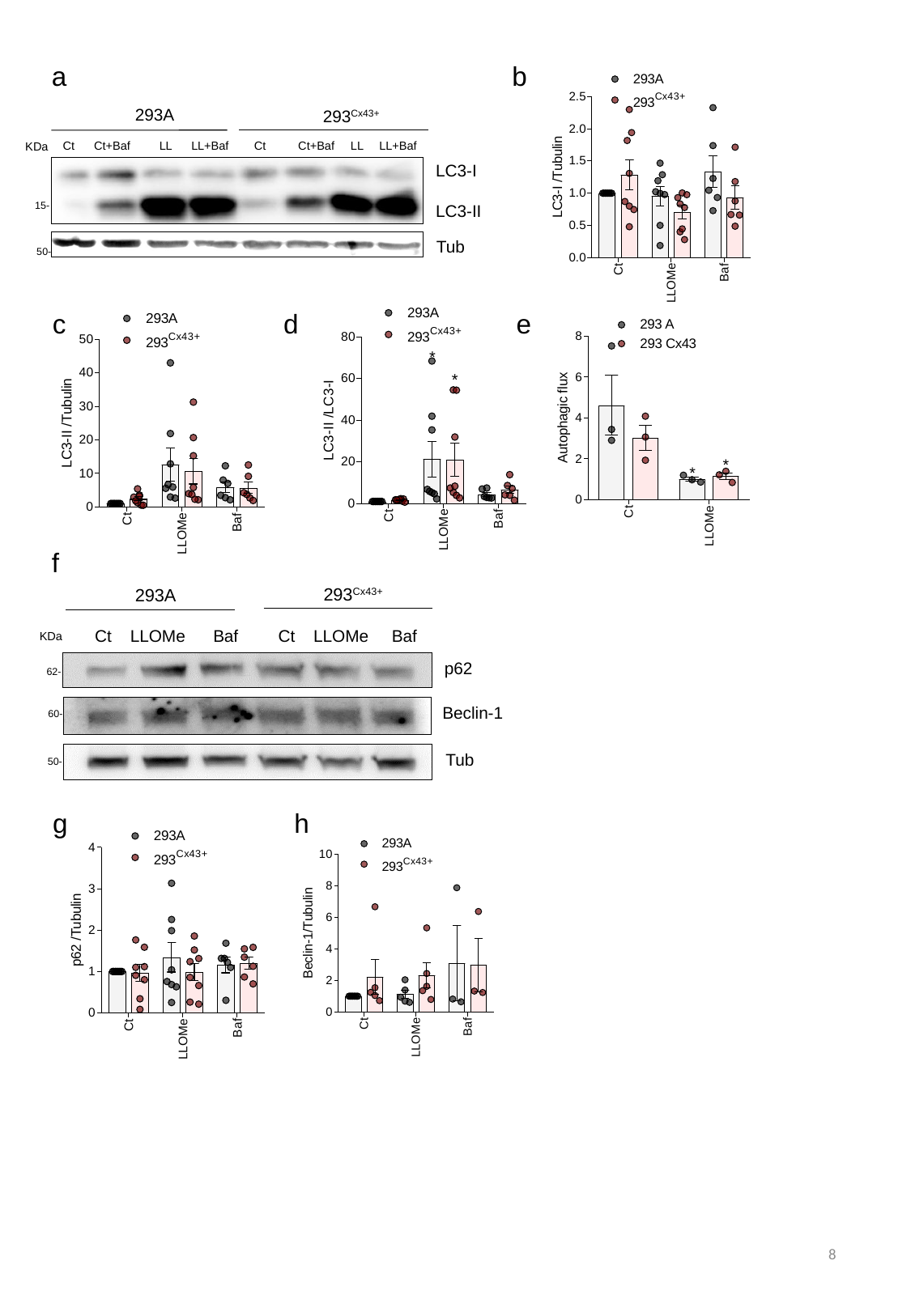

a
b
293A
293Cx43+
Ct Ct+Baf LL LL+Baf
Ct Ct+Baf LL LL+Baf
LC3-I
LC3-II
Tub
KDa
15-
50-
c
d
e
f
293Cx43+
293A
Ct LLOMe Baf
Ct LLOMe Baf
KDa
p62
62-
Beclin-1
60-
Tub
50-
g
h
8

## Slide 9
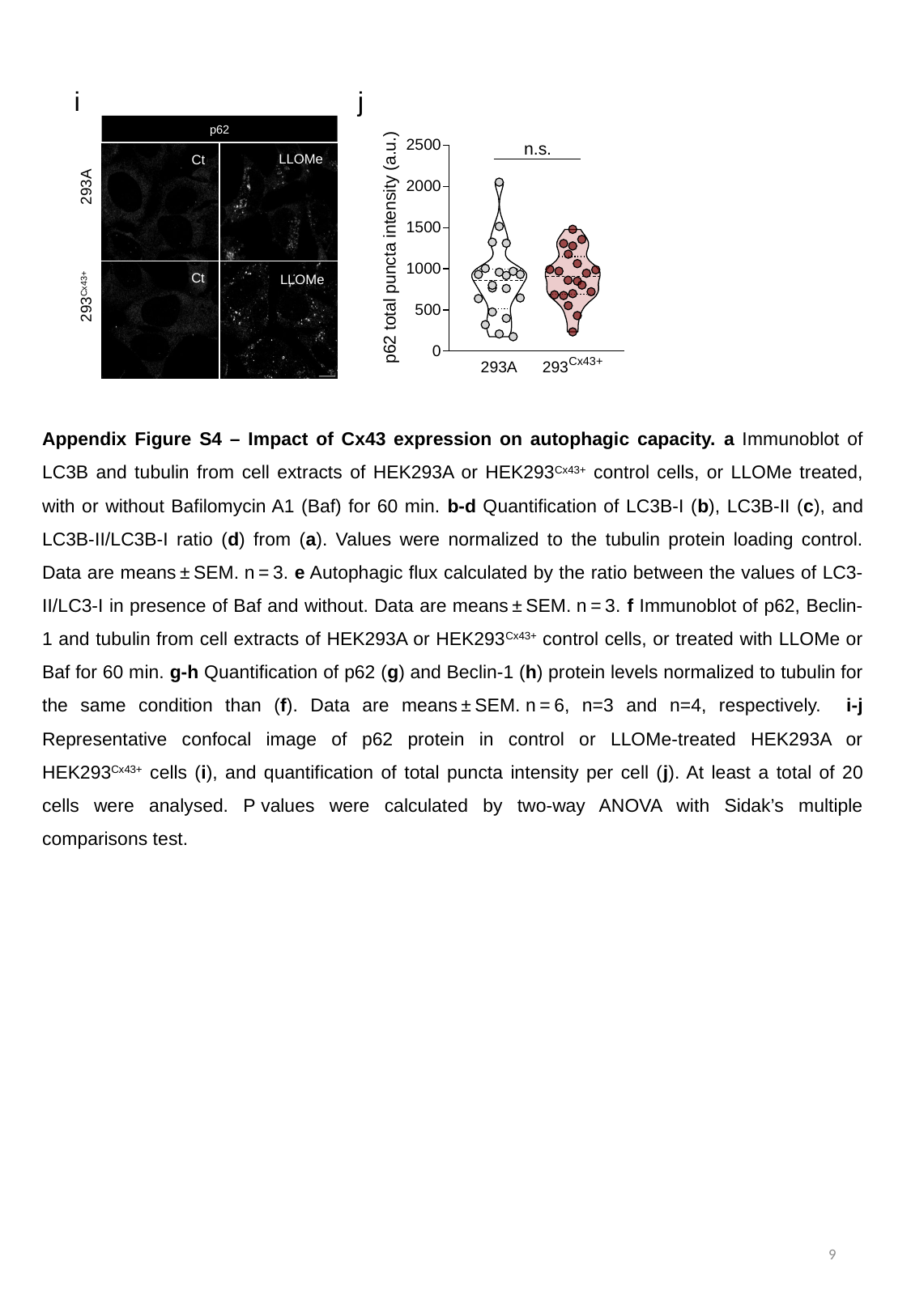

i
j
p62
LLOMe
Ct
293A
Ct
LLOMe
293Cx43+
Appendix Figure S4 – Impact of Cx43 expression on autophagic capacity. a Immunoblot of LC3B and tubulin from cell extracts of HEK293A or HEK293Cx43+ control cells, or LLOMe treated, with or without Bafilomycin A1 (Baf) for 60 min. b-d Quantification of LC3B-I (b), LC3B-II (c), and LC3B-II/LC3B-I ratio (d) from (a). Values were normalized to the tubulin protein loading control. Data are means ± SEM. n = 3. e Autophagic flux calculated by the ratio between the values of LC3-II/LC3-I in presence of Baf and without. Data are means ± SEM. n = 3. f Immunoblot of p62, Beclin-1 and tubulin from cell extracts of HEK293A or HEK293Cx43+ control cells, or treated with LLOMe or Baf for 60 min. g-h Quantification of p62 (g) and Beclin-1 (h) protein levels normalized to tubulin for the same condition than (f). Data are means ± SEM. n = 6, n=3 and n=4, respectively. i-j Representative confocal image of p62 protein in control or LLOMe-treated HEK293A or HEK293Cx43+ cells (i), and quantification of total puncta intensity per cell (j). At least a total of 20 cells were analysed. P values were calculated by two-way ANOVA with Sidak’s multiple comparisons test.
9

## Slide 10
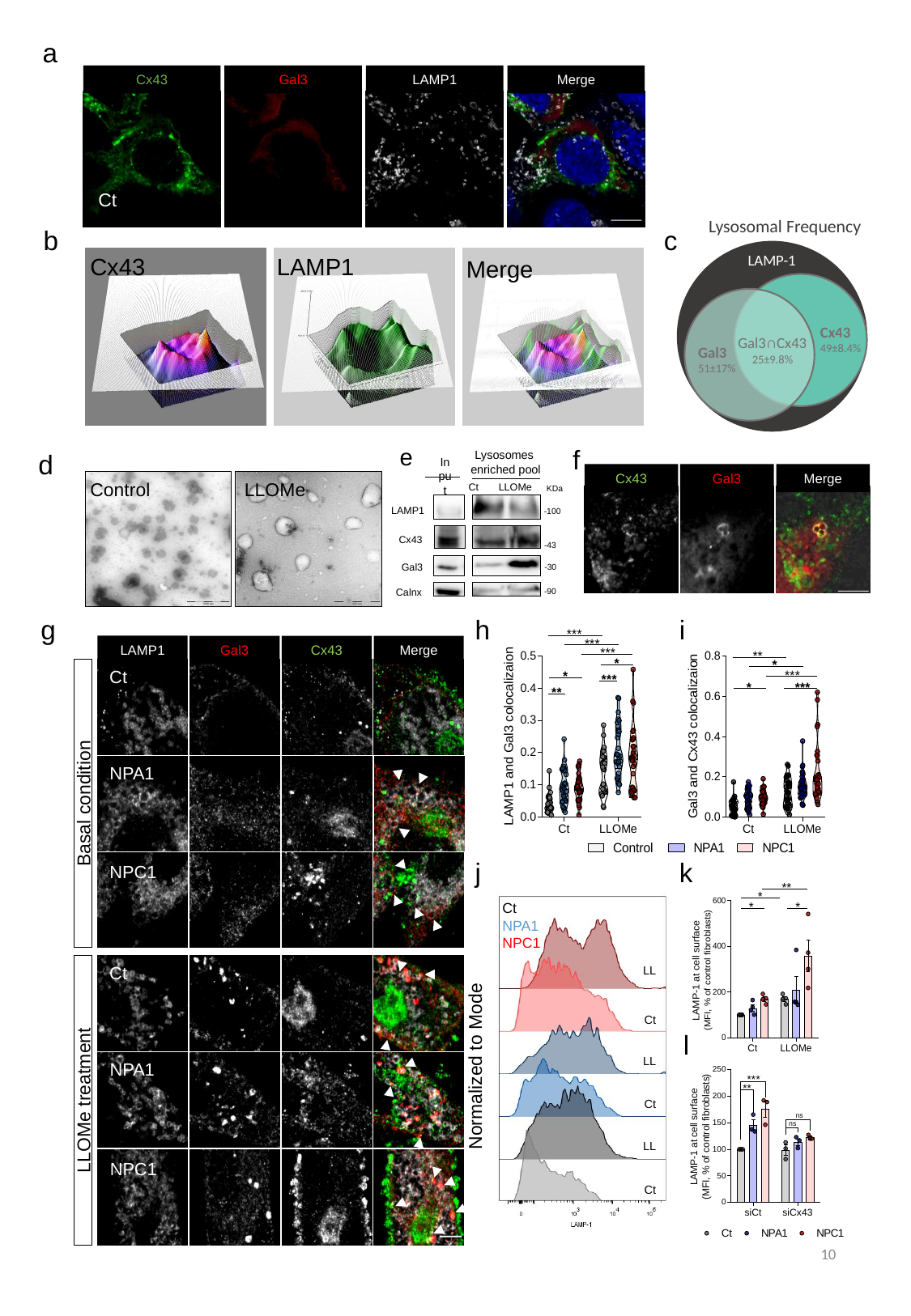

a
Cx43
Gal3
LAMP1
Merge
Ct
Lysosomal Frequency
LAMP-1
Cx43
49±8.4%
Gal3∩Cx43
25±9.8%
Gal3
51±17%
b
c
LAMP1
Cx43
Merge
e
f
d
Lysosomes
enriched pool
Input
 Ct LLOMe
LAMP1
Cx43
Gal3
Calnx
KDa
-100
-43
-30
-90
Cx43
Merge
Gal3
LLOMe
Control
g
h
i
LAMP1
Gal3
Cx43
Merge
Basal condition
Ct
NPA1
NPC1
LLOMe treatment
Ct
NPA1
NPC1
j
k
Ct
NPA1
NPC1
Normalized to Mode
LL
Ct
l
LL
Ct
LL
Ct
10

## Slide 11
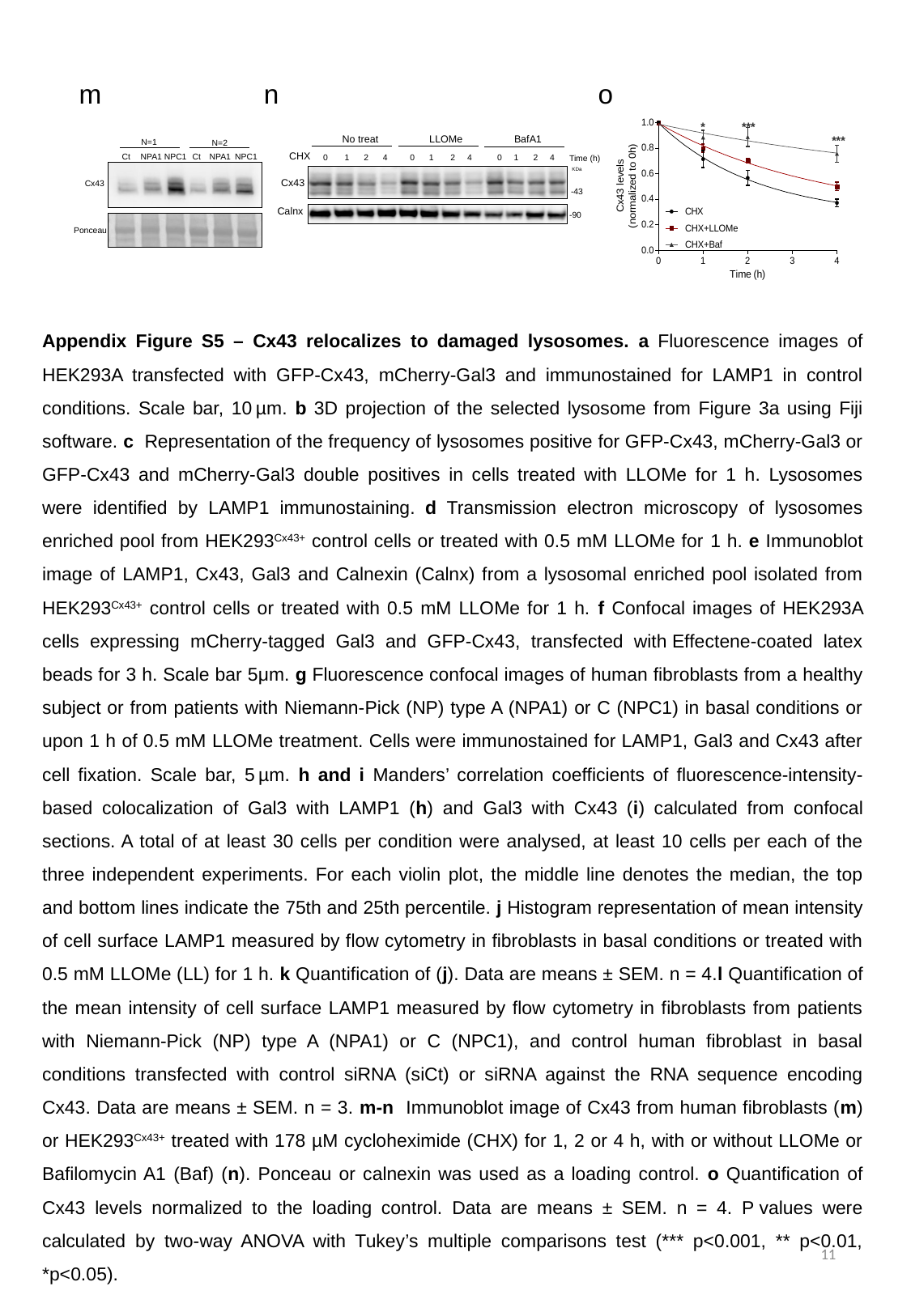

m
n
o
No treat
LLOMe
BafA1
CHX
0 1 2 4
0 1 2 4
0 1 2 4
Time (h)
KDa
Cx43
-43
Calnx
-90
N=1
N=2
NPC1
Ct
NPA1
NPC1
Ct
NPA1
Cx43
Ponceau
Appendix Figure S5 – Cx43 relocalizes to damaged lysosomes. a Fluorescence images of HEK293A transfected with GFP-Cx43, mCherry-Gal3 and immunostained for LAMP1 in control conditions. Scale bar, 10 µm. b 3D projection of the selected lysosome from Figure 3a using Fiji software. c Representation of the frequency of lysosomes positive for GFP-Cx43, mCherry-Gal3 or GFP-Cx43 and mCherry-Gal3 double positives in cells treated with LLOMe for 1 h. Lysosomes were identified by LAMP1 immunostaining. d Transmission electron microscopy of lysosomes enriched pool from HEK293Cx43+ control cells or treated with 0.5 mM LLOMe for 1 h. e Immunoblot image of LAMP1, Cx43, Gal3 and Calnexin (Calnx) from a lysosomal enriched pool isolated from HEK293Cx43+ control cells or treated with 0.5 mM LLOMe for 1 h. f Confocal images of HEK293A cells expressing mCherry-tagged Gal3 and GFP-Cx43, transfected with Effectene-coated latex beads for 3 h. Scale bar 5μm. g Fluorescence confocal images of human fibroblasts from a healthy subject or from patients with Niemann-Pick (NP) type A (NPA1) or C (NPC1) in basal conditions or upon 1 h of 0.5 mM LLOMe treatment. Cells were immunostained for LAMP1, Gal3 and Cx43 after cell fixation. Scale bar, 5 µm. h and i Manders’ correlation coefficients of fluorescence-intensity-based colocalization of Gal3 with LAMP1 (h) and Gal3 with Cx43 (i) calculated from confocal sections. A total of at least 30 cells per condition were analysed, at least 10 cells per each of the three independent experiments. For each violin plot, the middle line denotes the median, the top and bottom lines indicate the 75th and 25th percentile. j Histogram representation of mean intensity of cell surface LAMP1 measured by flow cytometry in fibroblasts in basal conditions or treated with 0.5 mM LLOMe (LL) for 1 h. k Quantification of (j). Data are means ± SEM. n = 4.l Quantification of the mean intensity of cell surface LAMP1 measured by flow cytometry in fibroblasts from patients with Niemann-Pick (NP) type A (NPA1) or C (NPC1), and control human fibroblast in basal conditions transfected with control siRNA (siCt) or siRNA against the RNA sequence encoding Cx43. Data are means ± SEM. n = 3. m-n Immunoblot image of Cx43 from human fibroblasts (m) or HEK293Cx43+ treated with 178 µM cycloheximide (CHX) for 1, 2 or 4 h, with or without LLOMe or Bafilomycin A1 (Baf) (n). Ponceau or calnexin was used as a loading control. o Quantification of Cx43 levels normalized to the loading control. Data are means ± SEM. n = 4. P values were calculated by two-way ANOVA with Tukey’s multiple comparisons test (*** p<0.001, ** p<0.01, *p<0.05).
11

## Slide 12
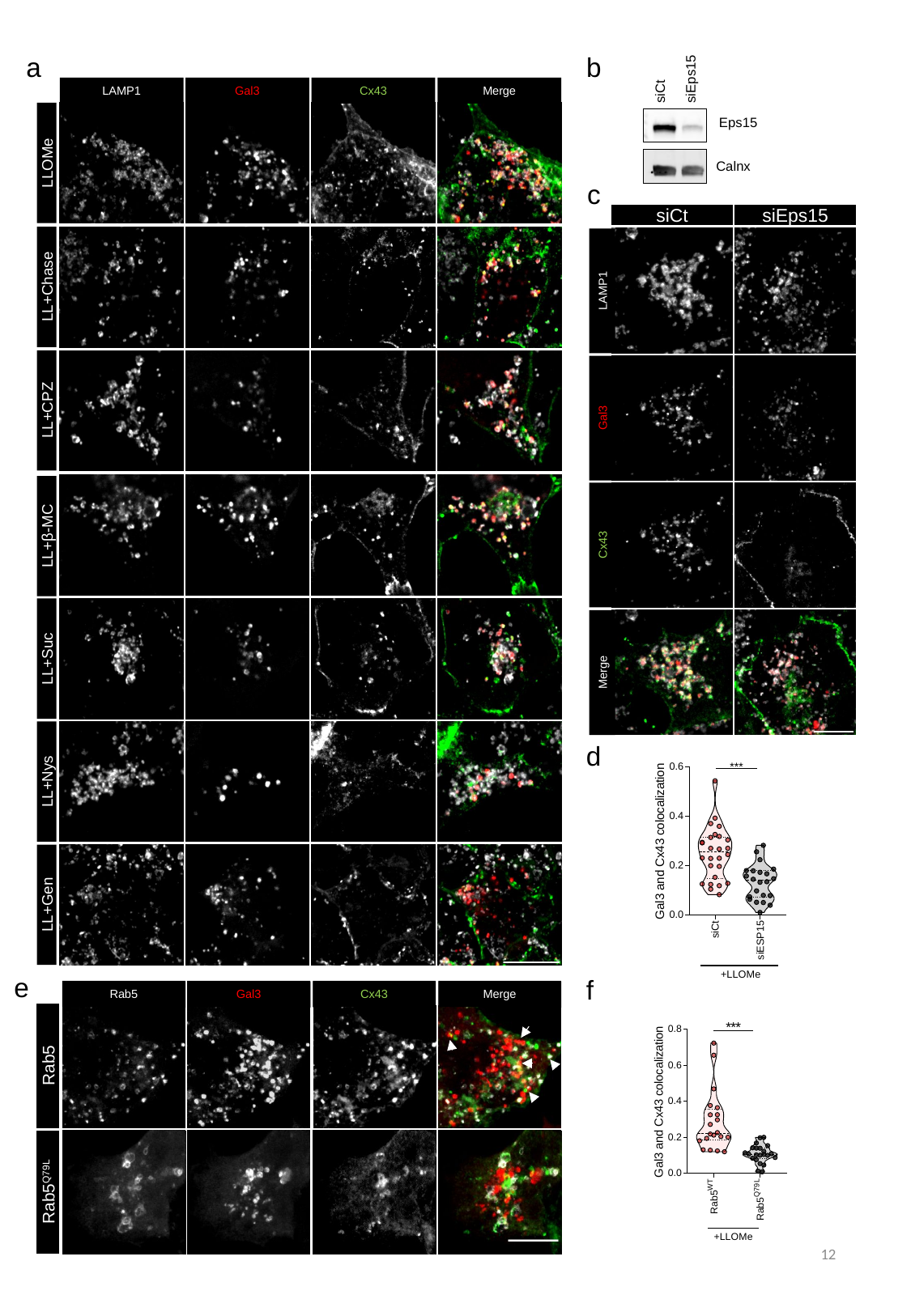

siEps15
siCt
Eps15
Calnx
a
b
LAMP1
Gal3
Cx43
Merge
LLOMe
LL+Chase
LL+CPZ
LL+β-MC
LL+Suc
LL+Nys
LL+Gen
siCt
siEps15
siCt
c
LAMP1
Gal3
Cx43
Merge
d
e
f
Rab5
Gal3
Cx43
Merge
Rab5
Rab5Q79L
Rab5
Gal3
Cx43
Merge
Rab5
Rab5Q79L
12

## Slide 13
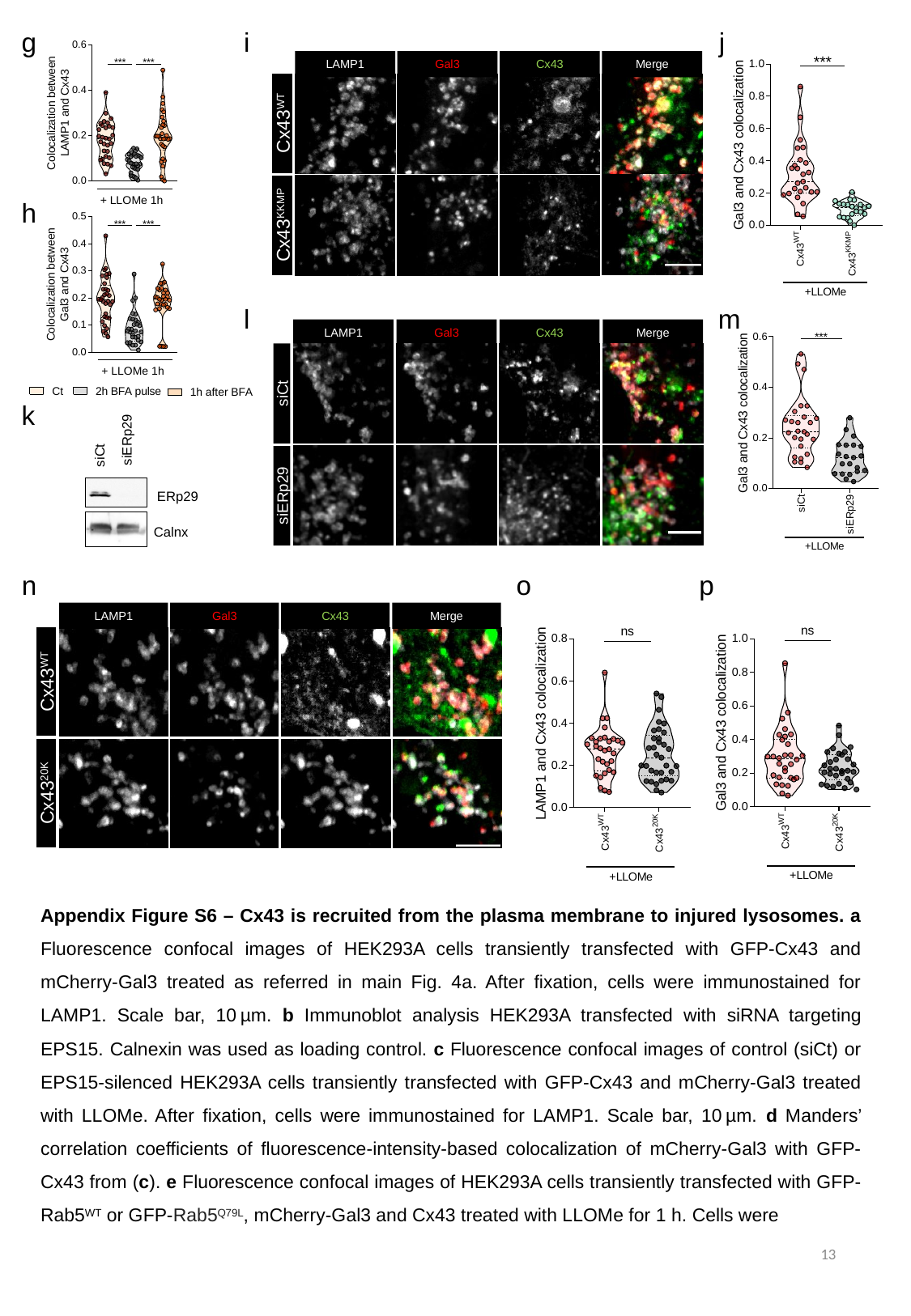

g
i
j
LAMP1
Gal3
Cx43
Merge
Cx43WT
Cx43KKMP
h
l
m
LAMP1
Gal3
Cx43
Merge
siCt
siERp29
k
siERp29
siCt
ERp29
Calnx
n
o
p
LAMP1
Gal3
Cx43
Merge
Cx43WT
Cx4320K
Appendix Figure S6 – Cx43 is recruited from the plasma membrane to injured lysosomes. a Fluorescence confocal images of HEK293A cells transiently transfected with GFP-Cx43 and mCherry-Gal3 treated as referred in main Fig. 4a. After fixation, cells were immunostained for LAMP1. Scale bar, 10 µm. b Immunoblot analysis HEK293A transfected with siRNA targeting EPS15. Calnexin was used as loading control. c Fluorescence confocal images of control (siCt) or EPS15-silenced HEK293A cells transiently transfected with GFP-Cx43 and mCherry-Gal3 treated with LLOMe. After fixation, cells were immunostained for LAMP1. Scale bar, 10 µm. d Manders’ correlation coefficients of fluorescence-intensity-based colocalization of mCherry-Gal3 with GFP-Cx43 from (c). e Fluorescence confocal images of HEK293A cells transiently transfected with GFP-Rab5WT or GFP-Rab5Q79L, mCherry-Gal3 and Cx43 treated with LLOMe for 1 h. Cells were
13

## Slide 14
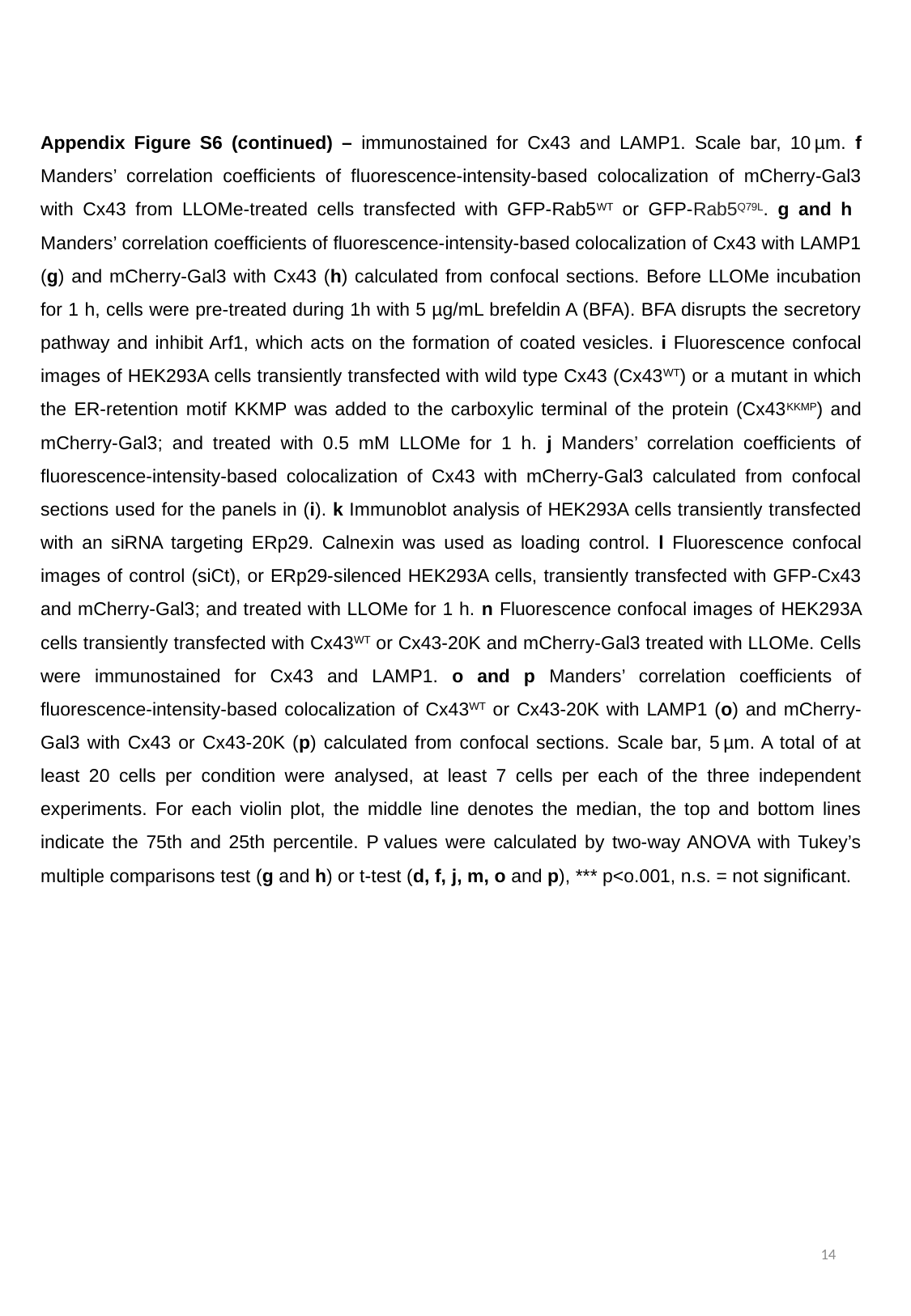

Appendix Figure S6 (continued) – immunostained for Cx43 and LAMP1. Scale bar, 10 µm. f Manders’ correlation coefficients of fluorescence-intensity-based colocalization of mCherry-Gal3 with Cx43 from LLOMe-treated cells transfected with GFP-Rab5WT or GFP-Rab5Q79L. g and h Manders’ correlation coefficients of fluorescence-intensity-based colocalization of Cx43 with LAMP1 (g) and mCherry-Gal3 with Cx43 (h) calculated from confocal sections. Before LLOMe incubation for 1 h, cells were pre-treated during 1h with 5 µg/mL brefeldin A (BFA). BFA disrupts the secretory pathway and inhibit Arf1, which acts on the formation of coated vesicles. i Fluorescence confocal images of HEK293A cells transiently transfected with wild type Cx43 (Cx43WT) or a mutant in which the ER-retention motif KKMP was added to the carboxylic terminal of the protein (Cx43KKMP) and mCherry-Gal3; and treated with 0.5 mM LLOMe for 1 h. j Manders’ correlation coefficients of fluorescence-intensity-based colocalization of Cx43 with mCherry-Gal3 calculated from confocal sections used for the panels in (i). k Immunoblot analysis of HEK293A cells transiently transfected with an siRNA targeting ERp29. Calnexin was used as loading control. l Fluorescence confocal images of control (siCt), or ERp29-silenced HEK293A cells, transiently transfected with GFP-Cx43 and mCherry-Gal3; and treated with LLOMe for 1 h. n Fluorescence confocal images of HEK293A cells transiently transfected with Cx43WT or Cx43-20K and mCherry-Gal3 treated with LLOMe. Cells were immunostained for Cx43 and LAMP1. o and p Manders’ correlation coefficients of fluorescence-intensity-based colocalization of Cx43WT or Cx43-20K with LAMP1 (o) and mCherry-Gal3 with Cx43 or Cx43-20K (p) calculated from confocal sections. Scale bar, 5 µm. A total of at least 20 cells per condition were analysed, at least 7 cells per each of the three independent experiments. For each violin plot, the middle line denotes the median, the top and bottom lines indicate the 75th and 25th percentile. P values were calculated by two-way ANOVA with Tukey’s multiple comparisons test (g and h) or t-test (d, f, j, m, o and p), *** p<o.001, n.s. = not significant.
14

## Slide 15
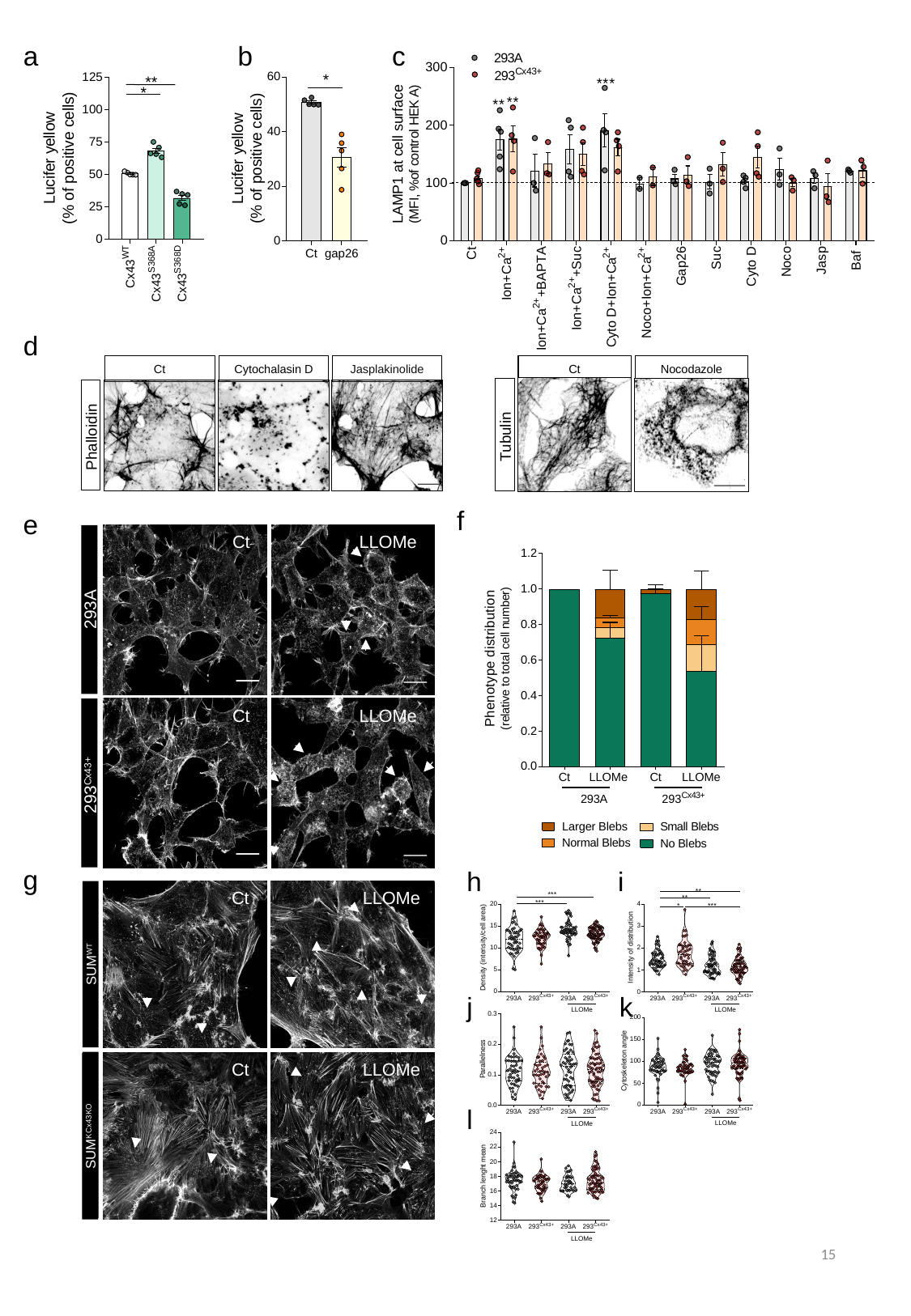

a
b
c
d
Ct
Jasplakinolide
Cytochalasin D
Phalloidin
Ct
Nocodazole
Tubulin
f
e
Ct
LLOMe
293A
Ct
LLOMe
293Cx43+
g
h
i
Ct
LLOMe
SUMWT
Ct
LLOMe
293Cx43+
SUMKCx43KO
j
k
l
15

## Slide 16
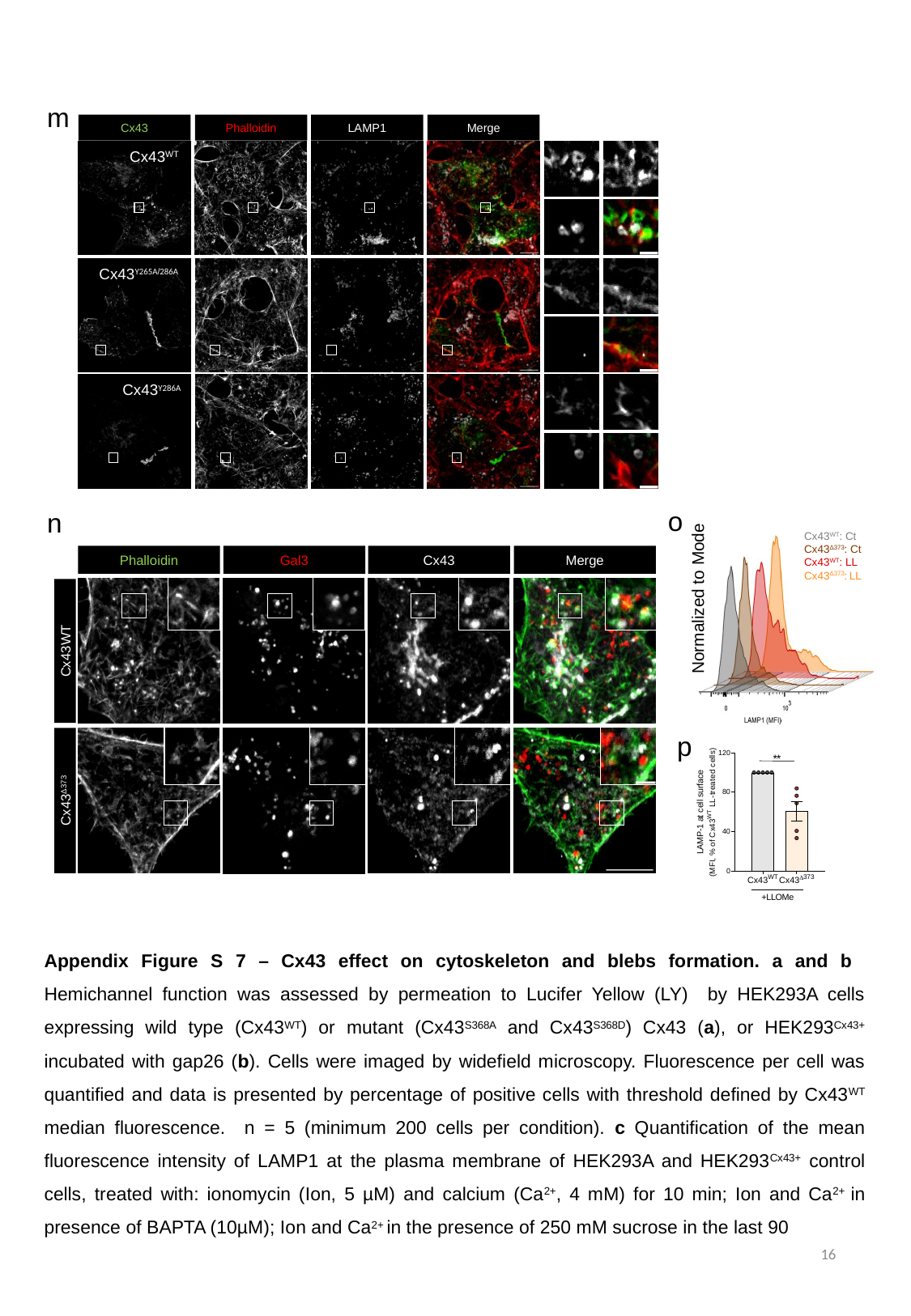

Phalloidin
LAMP1
Merge
Cx43
Cx43WT
Cx43Y265A/286A
Cx43Y286A
m
o
n
Normalized to Mode
Cx43WT: Ct
Cx43Δ373: Ct
Cx43WT: LL
Cx43Δ373: LL
Phalloidin
Gal3
Cx43
Merge
Cx43WT
Cx43Δ373
p
Appendix Figure S 7 – Cx43 effect on cytoskeleton and blebs formation. a and b Hemichannel function was assessed by permeation to Lucifer Yellow (LY) by HEK293A cells expressing wild type (Cx43WT) or mutant (Cx43S368A and Cx43S368D) Cx43 (a), or HEK293Cx43+ incubated with gap26 (b). Cells were imaged by widefield microscopy. Fluorescence per cell was quantified and data is presented by percentage of positive cells with threshold defined by Cx43WT median fluorescence. n = 5 (minimum 200 cells per condition). c Quantification of the mean fluorescence intensity of LAMP1 at the plasma membrane of HEK293A and HEK293Cx43+ control cells, treated with: ionomycin (Ion, 5 µM) and calcium (Ca2+, 4 mM) for 10 min; Ion and Ca2+ in presence of BAPTA (10µM); Ion and Ca2+ in the presence of 250 mM sucrose in the last 90
16

## Slide 17
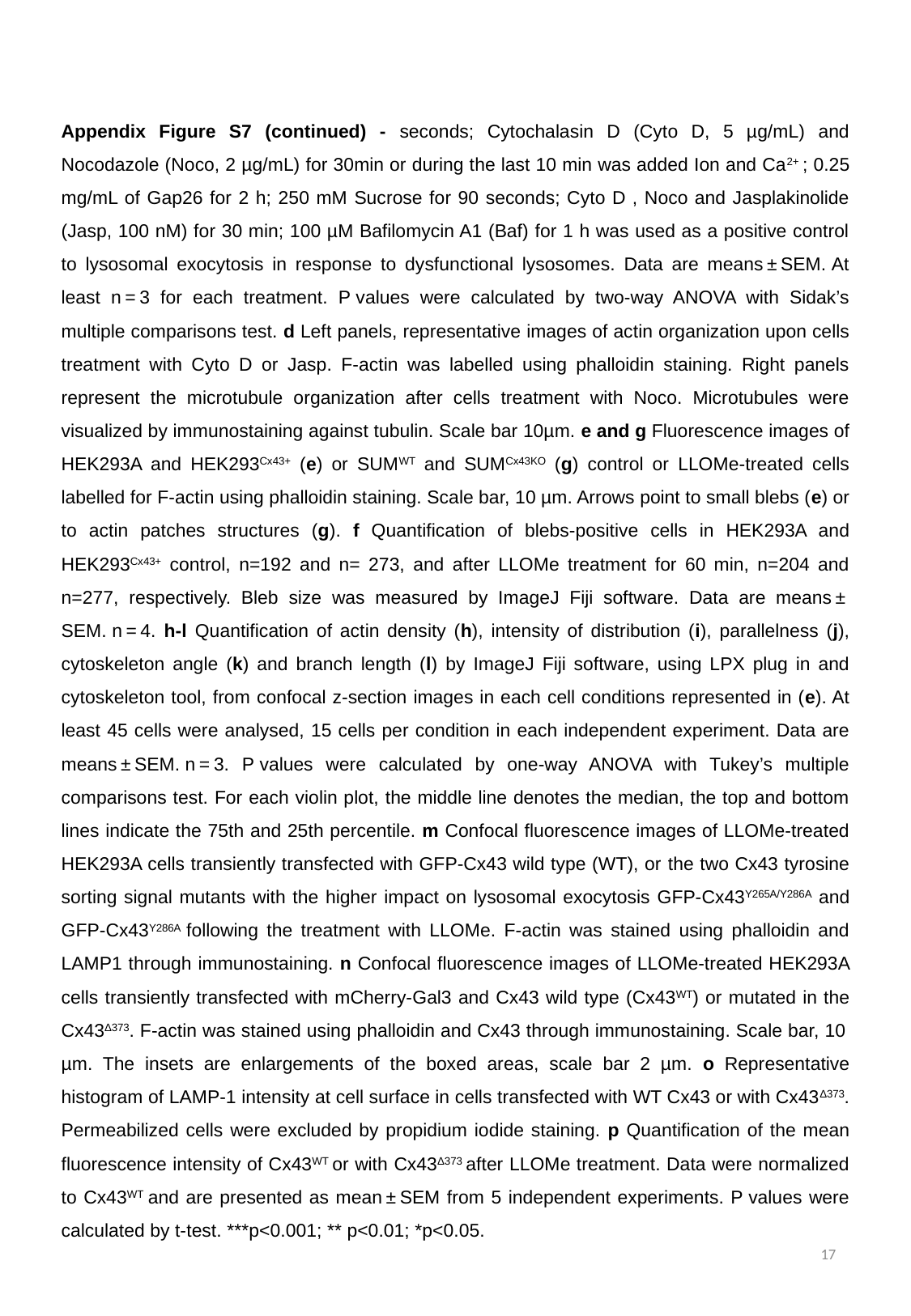

Appendix Figure S7 (continued) - seconds; Cytochalasin D (Cyto D, 5 µg/mL) and Nocodazole (Noco, 2 µg/mL) for 30min or during the last 10 min was added Ion and Ca2+ ; 0.25 mg/mL of Gap26 for 2 h; 250 mM Sucrose for 90 seconds; Cyto D , Noco and Jasplakinolide (Jasp, 100 nM) for 30 min; 100 µM Bafilomycin A1 (Baf) for 1 h was used as a positive control to lysosomal exocytosis in response to dysfunctional lysosomes. Data are means ± SEM. At least n = 3 for each treatment. P values were calculated by two-way ANOVA with Sidak’s multiple comparisons test. d Left panels, representative images of actin organization upon cells treatment with Cyto D or Jasp. F-actin was labelled using phalloidin staining. Right panels represent the microtubule organization after cells treatment with Noco. Microtubules were visualized by immunostaining against tubulin. Scale bar 10µm. e and g Fluorescence images of HEK293A and HEK293Cx43+ (e) or SUMWT and SUMCx43KO (g) control or LLOMe-treated cells labelled for F-actin using phalloidin staining. Scale bar, 10 µm. Arrows point to small blebs (e) or to actin patches structures (g). f Quantification of blebs-positive cells in HEK293A and HEK293Cx43+ control, n=192 and n= 273, and after LLOMe treatment for 60 min, n=204 and n=277, respectively. Bleb size was measured by ImageJ Fiji software. Data are means ± SEM. n = 4. h-l Quantification of actin density (h), intensity of distribution (i), parallelness (j), cytoskeleton angle (k) and branch length (l) by ImageJ Fiji software, using LPX plug in and cytoskeleton tool, from confocal z-section images in each cell conditions represented in (e). At least 45 cells were analysed, 15 cells per condition in each independent experiment. Data are means ± SEM. n = 3. P values were calculated by one-way ANOVA with Tukey’s multiple comparisons test. For each violin plot, the middle line denotes the median, the top and bottom lines indicate the 75th and 25th percentile. m Confocal fluorescence images of LLOMe-treated HEK293A cells transiently transfected with GFP-Cx43 wild type (WT), or the two Cx43 tyrosine sorting signal mutants with the higher impact on lysosomal exocytosis GFP-Cx43Y265A/Y286A and GFP-Cx43Y286A following the treatment with LLOMe. F-actin was stained using phalloidin and LAMP1 through immunostaining. n Confocal fluorescence images of LLOMe-treated HEK293A cells transiently transfected with mCherry-Gal3 and Cx43 wild type (Cx43WT) or mutated in the Cx43Δ373. F-actin was stained using phalloidin and Cx43 through immunostaining. Scale bar, 10 µm. The insets are enlargements of the boxed areas, scale bar 2 µm. o Representative histogram of LAMP-1 intensity at cell surface in cells transfected with WT Cx43 or with Cx43Δ373. Permeabilized cells were excluded by propidium iodide staining. p Quantification of the mean fluorescence intensity of Cx43WT or with Cx43Δ373 after LLOMe treatment. Data were normalized to Cx43WT and are presented as mean ± SEM from 5 independent experiments. P values were calculated by t-test. ***p<0.001; ** p<0.01; *p<0.05.
17

## Slide 18
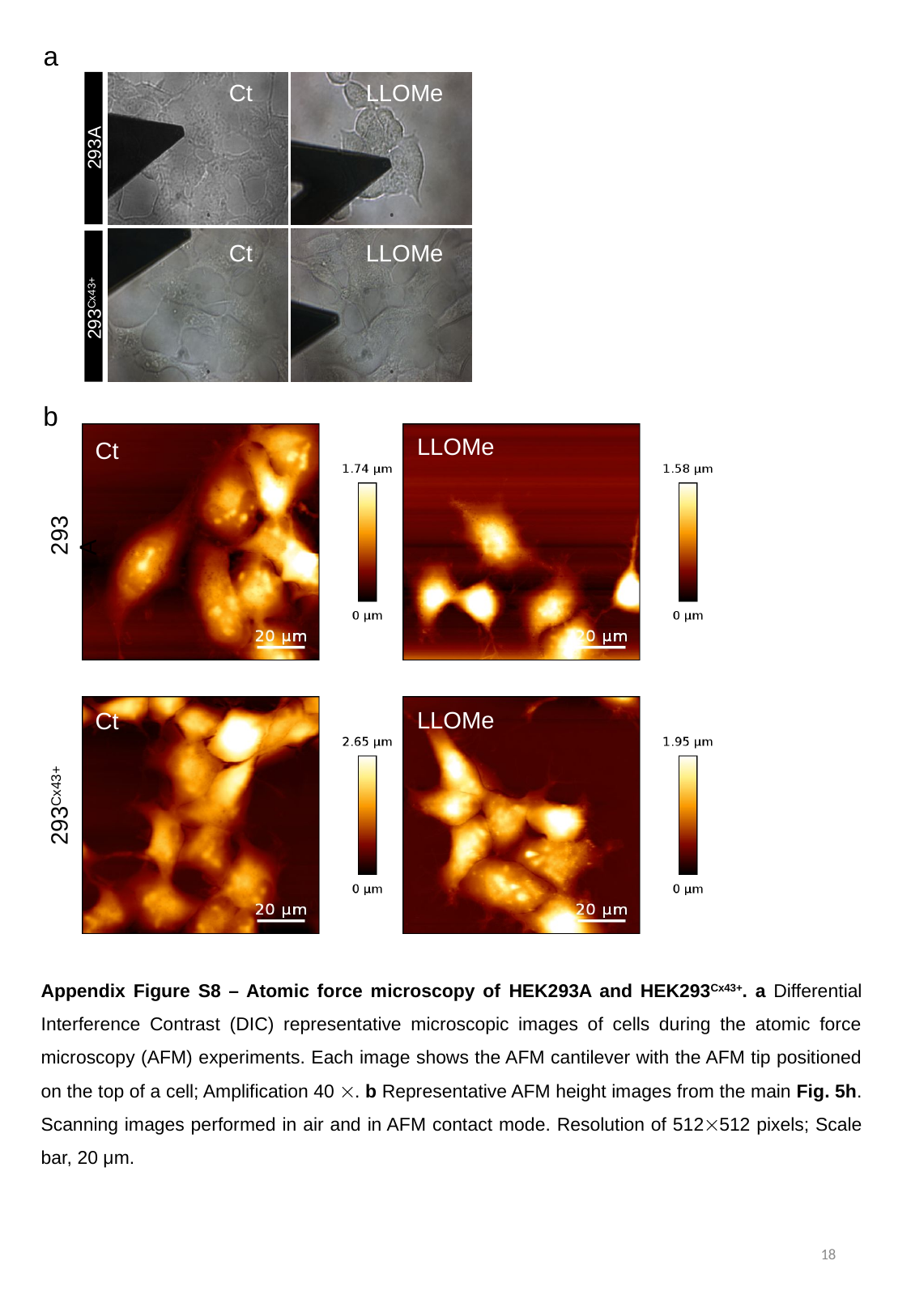

a
Ct
LLOMe
293A
Ct
LLOMe
293Cx43+
b
LLOMe
Ct
293A
LLOMe
Ct
293Cx43+
Appendix Figure S8 – Atomic force microscopy of HEK293A and HEK293Cx43+. a Differential Interference Contrast (DIC) representative microscopic images of cells during the atomic force microscopy (AFM) experiments. Each image shows the AFM cantilever with the AFM tip positioned on the top of a cell; Amplification 40 ×. b Representative AFM height images from the main Fig. 5h. Scanning images performed in air and in AFM contact mode. Resolution of 512×512 pixels; Scale bar, 20 μm.
18

## Slide 19
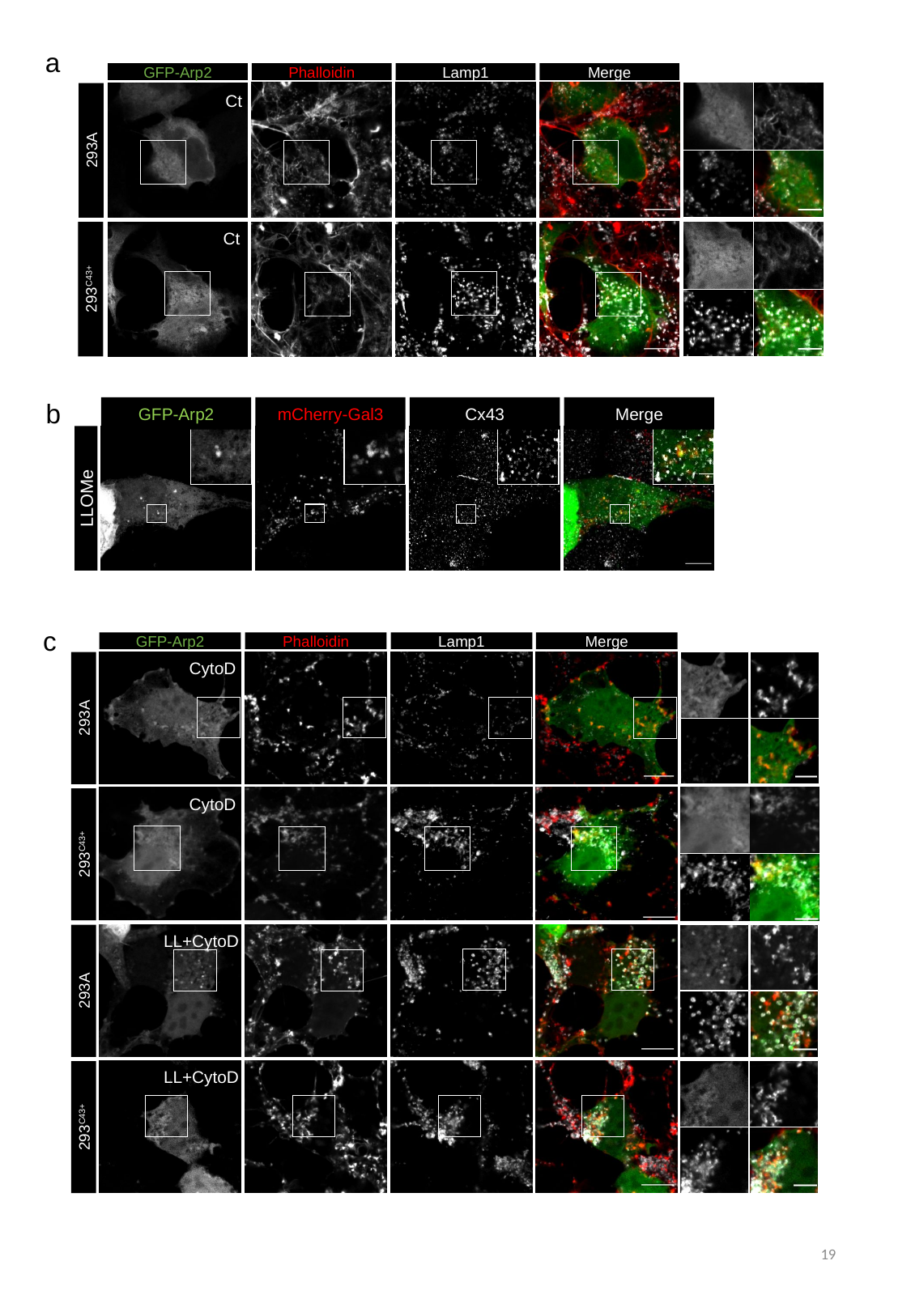

a
GFP-Arp2
Phalloidin
Lamp1
Merge
293A
293C43+
Ct
Ct
b
GFP-Arp2
mCherry-Gal3
Cx43
Merge
LLOMe
c
GFP-Arp2
Phalloidin
Lamp1
Merge
293A
293C43+
293A
293C43+
CytoD
CytoD
LL+CytoD
LL+CytoD
19

## Slide 20
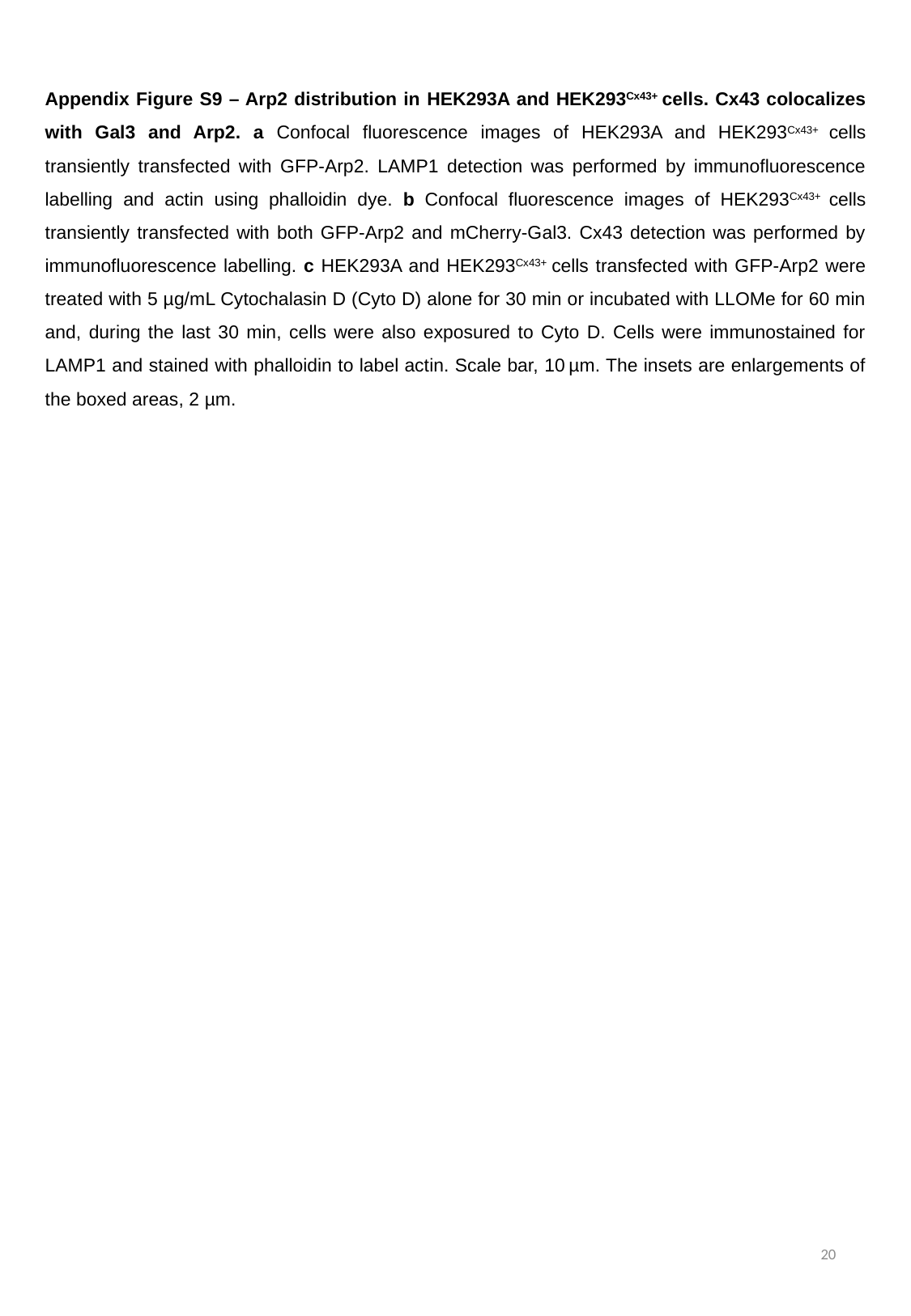

Appendix Figure S9 – Arp2 distribution in HEK293A and HEK293Cx43+ cells. Cx43 colocalizes with Gal3 and Arp2. a Confocal fluorescence images of HEK293A and HEK293Cx43+ cells transiently transfected with GFP-Arp2. LAMP1 detection was performed by immunofluorescence labelling and actin using phalloidin dye. b Confocal fluorescence images of HEK293Cx43+ cells transiently transfected with both GFP-Arp2 and mCherry-Gal3. Cx43 detection was performed by immunofluorescence labelling. c HEK293A and HEK293Cx43+ cells transfected with GFP-Arp2 were treated with 5 µg/mL Cytochalasin D (Cyto D) alone for 30 min or incubated with LLOMe for 60 min and, during the last 30 min, cells were also exposured to Cyto D. Cells were immunostained for LAMP1 and stained with phalloidin to label actin. Scale bar, 10 µm. The insets are enlargements of the boxed areas, 2 µm.
20

## Slide 21
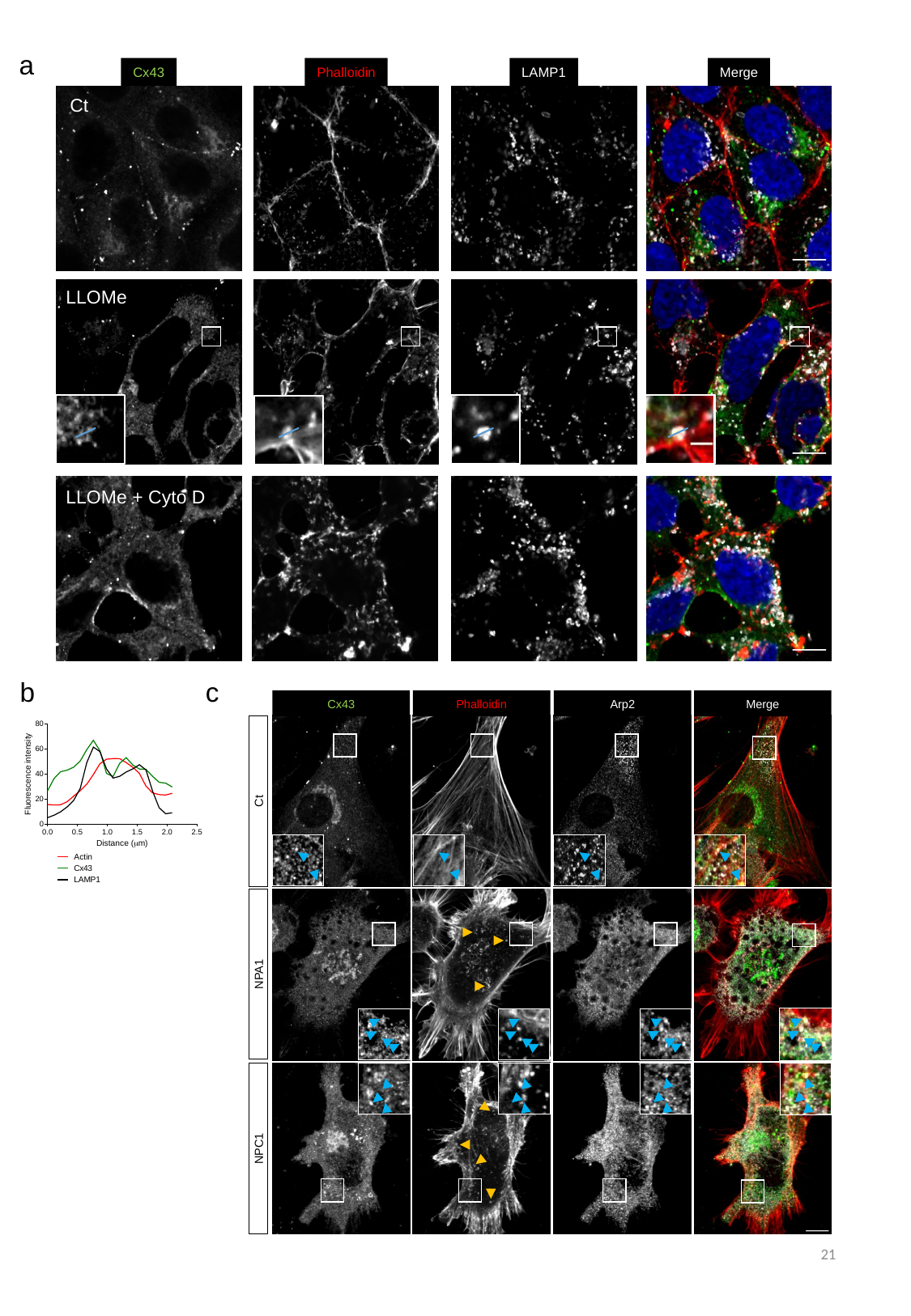

a
Cx43
Phalloidin
LAMP1
Merge
Ct
LLOMe
LLOMe + Cyto D
b
c
Cx43
Phalloidin
Arp2
Merge
Ct
NPA1
NPC1
21

## Slide 22
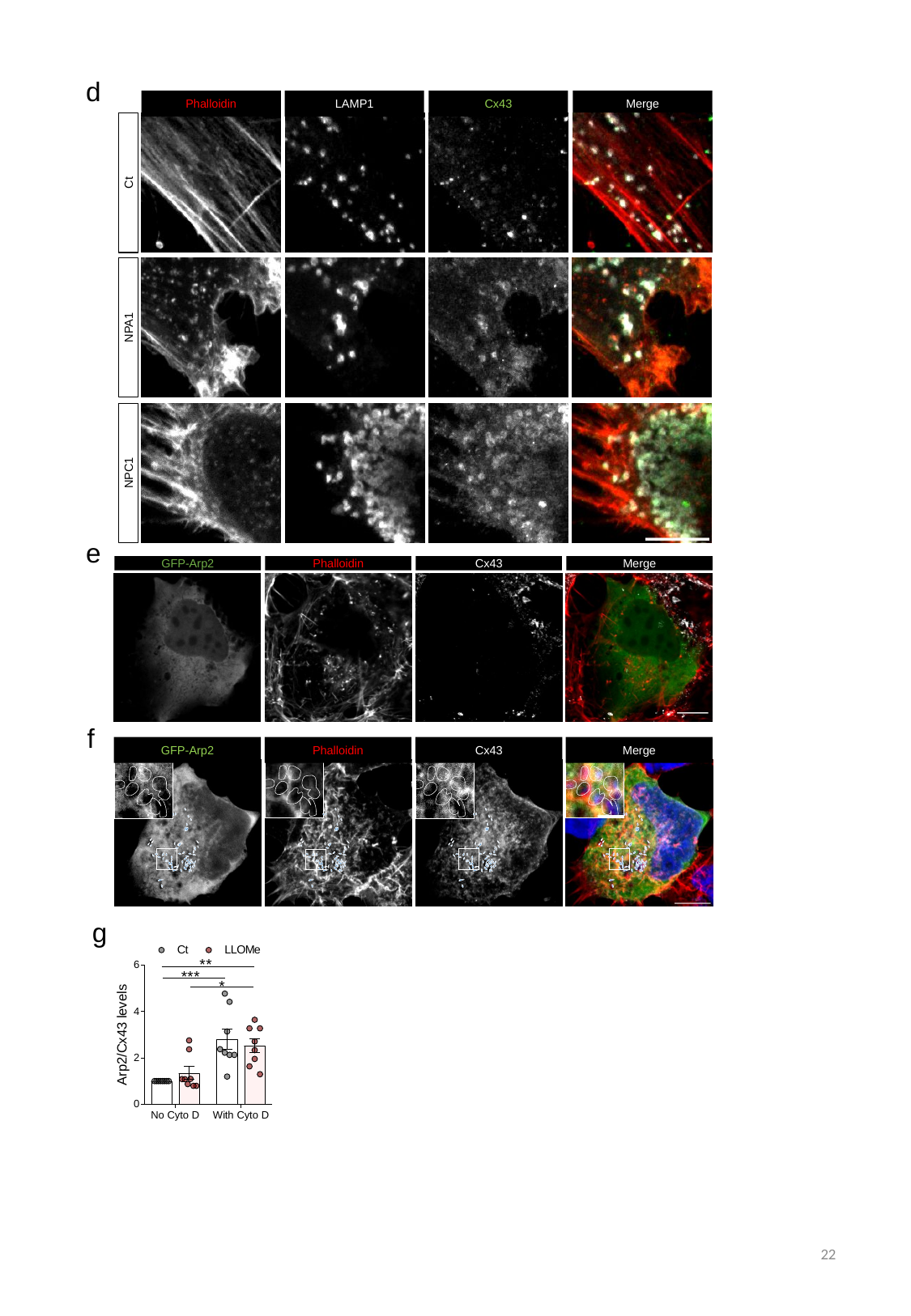

d
LAMP1
Phalloidin
Cx43
Merge
Ct
NPA1
NPC1
e
GFP-Arp2
Phalloidin
Cx43
Merge
f
GFP-Arp2
Phalloidin
Cx43
Merge
g
22

## Slide 23
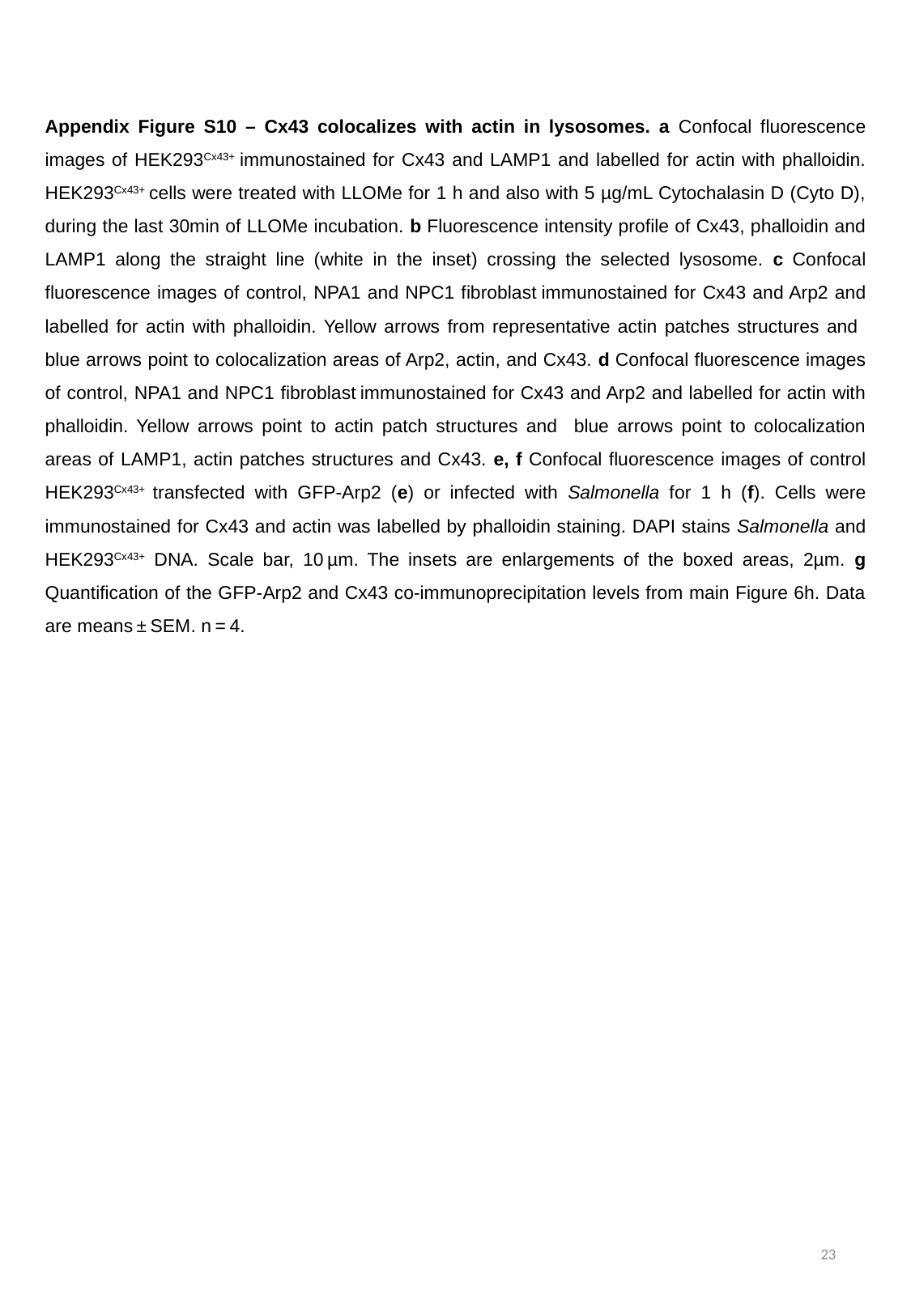

Appendix Figure S10 – Cx43 colocalizes with actin in lysosomes. a Confocal fluorescence images of HEK293Cx43+ immunostained for Cx43 and LAMP1 and labelled for actin with phalloidin. HEK293Cx43+ cells were treated with LLOMe for 1 h and also with 5 µg/mL Cytochalasin D (Cyto D), during the last 30min of LLOMe incubation. b Fluorescence intensity profile of Cx43, phalloidin and LAMP1 along the straight line (white in the inset) crossing the selected lysosome. c Confocal fluorescence images of control, NPA1 and NPC1 fibroblast immunostained for Cx43 and Arp2 and labelled for actin with phalloidin. Yellow arrows from representative actin patches structures and blue arrows point to colocalization areas of Arp2, actin, and Cx43. d Confocal fluorescence images of control, NPA1 and NPC1 fibroblast immunostained for Cx43 and Arp2 and labelled for actin with phalloidin. Yellow arrows point to actin patch structures and blue arrows point to colocalization areas of LAMP1, actin patches structures and Cx43. e, f Confocal fluorescence images of control HEK293Cx43+ transfected with GFP-Arp2 (e) or infected with Salmonella for 1 h (f). Cells were immunostained for Cx43 and actin was labelled by phalloidin staining. DAPI stains Salmonella and HEK293Cx43+ DNA. Scale bar, 10 µm. The insets are enlargements of the boxed areas, 2µm. g Quantification of the GFP-Arp2 and Cx43 co-immunoprecipitation levels from main Figure 6h. Data are means ± SEM. n = 4.
23

## Slide 24
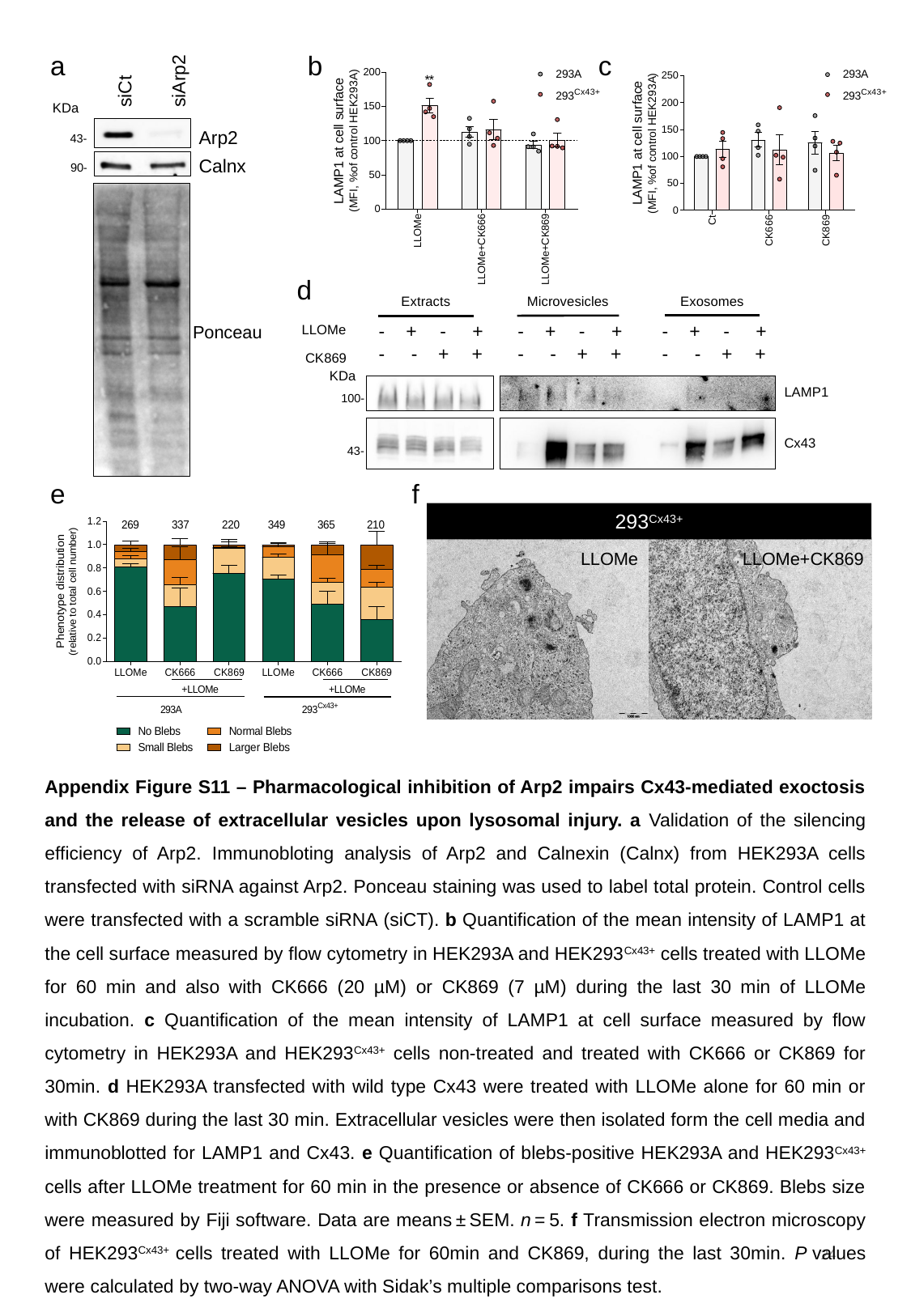

siArp2
siCt
Arp2
Calnx
KDa
43-
90-
a
b
c
d
Exosomes
Microvesicles
Extracts
- +
- +
- +
- +
- +
- +
LLOMe
- -
+
+
- -
+
+
- -
+
+
CK869
LAMP1
Cx43
KDa
100-
43-
Ponceau
e
f
293Cx43+
LLOMe
LLOMe+CK869
Appendix Figure S11 – Pharmacological inhibition of Arp2 impairs Cx43-mediated exoctosis and the release of extracellular vesicles upon lysosomal injury. a Validation of the silencing efficiency of Arp2. Immunobloting analysis of Arp2 and Calnexin (Calnx) from HEK293A cells transfected with siRNA against Arp2. Ponceau staining was used to label total protein. Control cells were transfected with a scramble siRNA (siCT). b Quantification of the mean intensity of LAMP1 at the cell surface measured by flow cytometry in HEK293A and HEK293Cx43+ cells treated with LLOMe for 60 min and also with CK666 (20 µM) or CK869 (7 µM) during the last 30 min of LLOMe incubation. c Quantification of the mean intensity of LAMP1 at cell surface measured by flow cytometry in HEK293A and HEK293Cx43+ cells non-treated and treated with CK666 or CK869 for 30min. d HEK293A transfected with wild type Cx43 were treated with LLOMe alone for 60 min or with CK869 during the last 30 min. Extracellular vesicles were then isolated form the cell media and immunoblotted for LAMP1 and Cx43. e Quantification of blebs-positive HEK293A and HEK293Cx43+ cells after LLOMe treatment for 60 min in the presence or absence of CK666 or CK869. Blebs size were measured by Fiji software. Data are means ± SEM. n = 5. f Transmission electron microscopy of HEK293Cx43+ cells treated with LLOMe for 60min and CK869, during the last 30min. P values were calculated by two-way ANOVA with Sidak’s multiple comparisons test.
24

## Slide 25
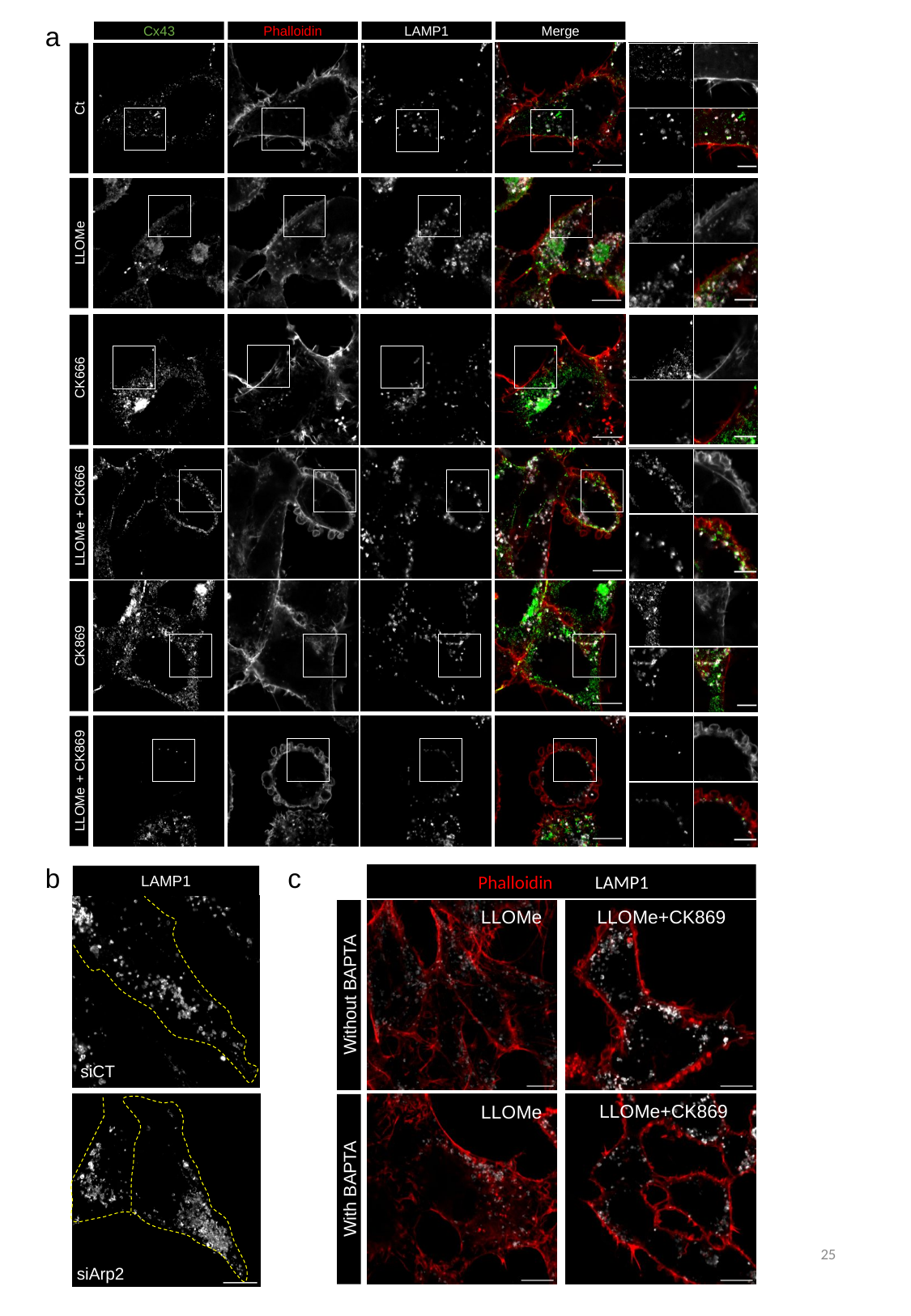

a
Cx43
Phalloidin
LAMP1
Merge
Ct
LLOMe
 CK666
LLOMe + CK666
CK869
LLOMe + CK869
b
c
 Phalloidin LAMP1
LLOMe+CK869
LLOMe
Without BAPTA
LLOMe+CK869
With BAPTA
LLOMe
LAMP1
siCT
siArp2
25

## Slide 26
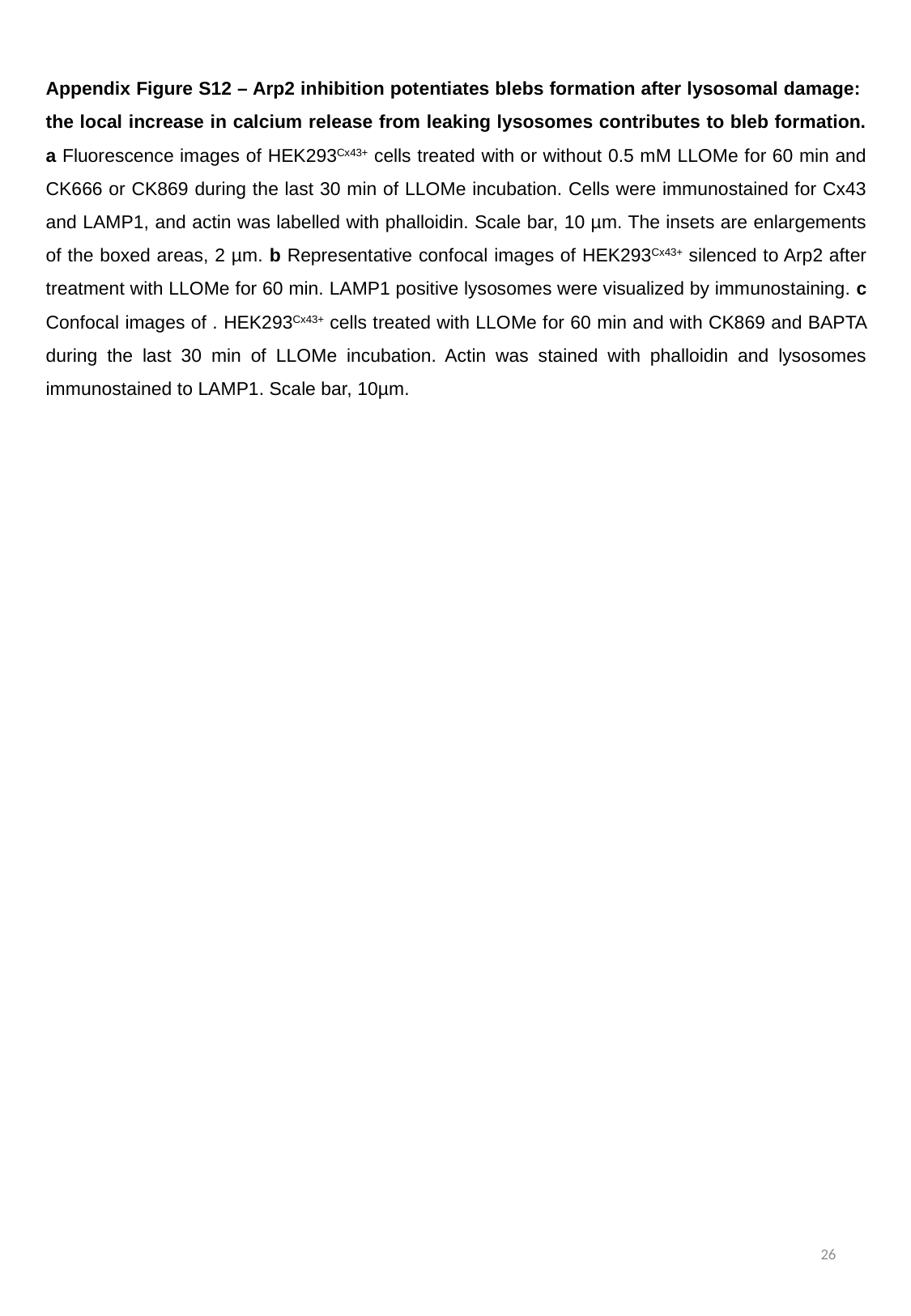

Appendix Figure S12 – Arp2 inhibition potentiates blebs formation after lysosomal damage: the local increase in calcium release from leaking lysosomes contributes to bleb formation. a Fluorescence images of HEK293Cx43+ cells treated with or without 0.5 mM LLOMe for 60 min and CK666 or CK869 during the last 30 min of LLOMe incubation. Cells were immunostained for Cx43 and LAMP1, and actin was labelled with phalloidin. Scale bar, 10 µm. The insets are enlargements of the boxed areas, 2 µm. b Representative confocal images of HEK293Cx43+ silenced to Arp2 after treatment with LLOMe for 60 min. LAMP1 positive lysosomes were visualized by immunostaining. c Confocal images of . HEK293Cx43+ cells treated with LLOMe for 60 min and with CK869 and BAPTA during the last 30 min of LLOMe incubation. Actin was stained with phalloidin and lysosomes immunostained to LAMP1. Scale bar, 10µm.
26

## Slide 27
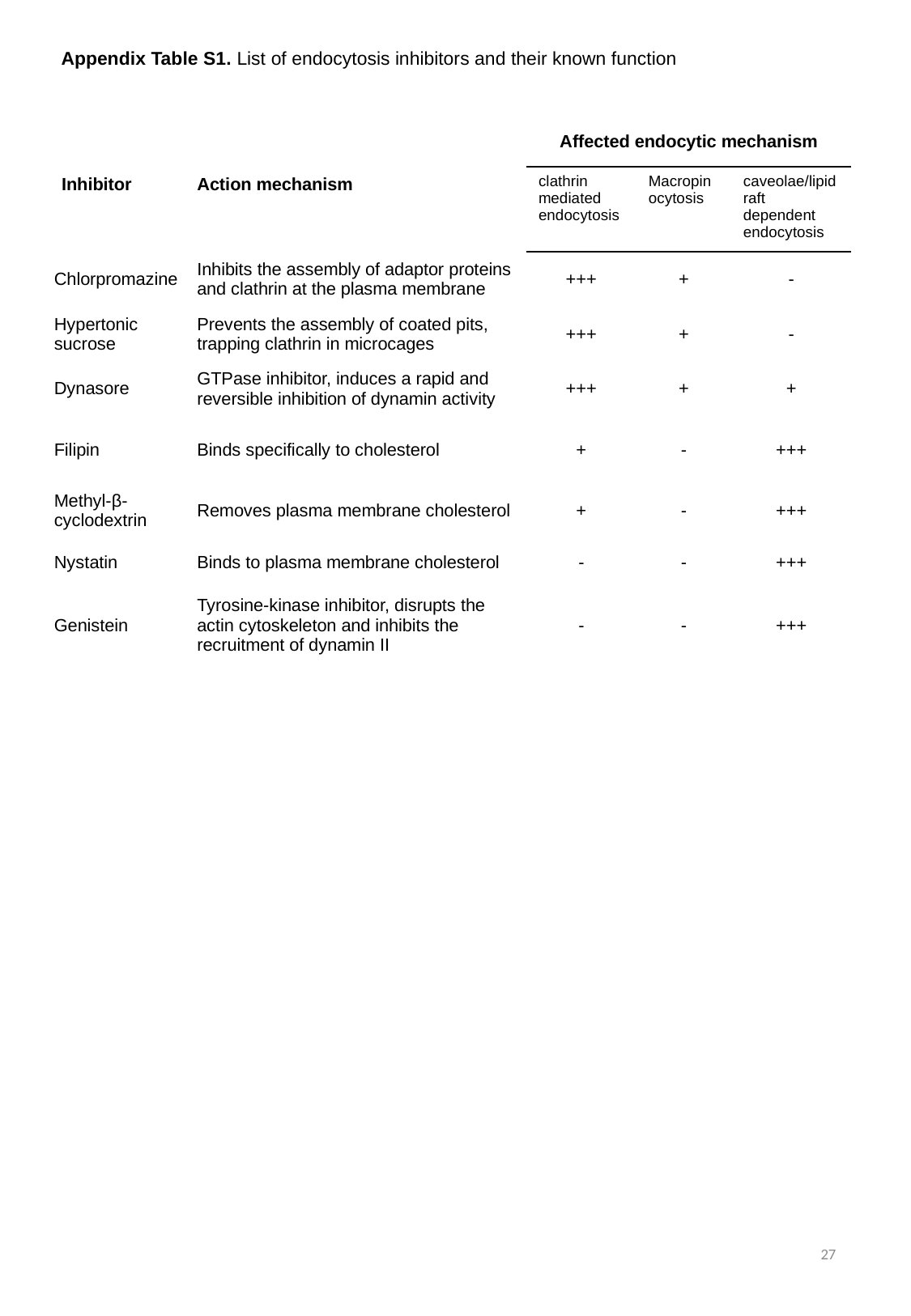

Appendix Table S1. List of endocytosis inhibitors and their known function
| Inhibitor | Action mechanism | Affected endocytic mechanism | | |
| --- | --- | --- | --- | --- |
| | | clathrin mediated endocytosis | Macropinocytosis | caveolae/lipid raft dependent endocytosis |
| Chlorpromazine | Inhibits the assembly of adaptor proteins and clathrin at the plasma membrane | +++ | + | - |
| Hypertonic sucrose | Prevents the assembly of coated pits, trapping clathrin in microcages | +++ | + | - |
| Dynasore | GTPase inhibitor, induces a rapid and reversible inhibition of dynamin activity | +++ | + | + |
| Filipin | Binds specifically to cholesterol | + | - | +++ |
| Methyl-β-cyclodextrin | Removes plasma membrane cholesterol | + | - | +++ |
| Nystatin | Binds to plasma membrane cholesterol | - | - | +++ |
| Genistein | Tyrosine-kinase inhibitor, disrupts the actin cytoskeleton and inhibits the recruitment of dynamin II | - | - | +++ |
27
