# Supplementary material for: Connexin43 promotes exocytosis of damaged lysosomes through actin remodelling
Source: EMBO J. 2024 Jul 23;43(17):3627–49. doi: 10.1038/s44318-024-00177-3 (PMC11377567; doi:10.1038/s44318-024-00177-3)
Supplement: Supplementary file 3 — Movie EV1 [file 44318_2024_177_MOESM3_ESM.zip › Movie EV1 legend.docx]

Movie EV1 – Cx43 partitions to Gal3-positive vesicles after LLOME treatment.

Time-lapse confocal imaging, through the acquisition of one z-stack image per 30 seconds for a total of 15 min, of HEK293A cells transiently transfected with GFP-Cx43 and mCherry-Gal3 and treated with 0.5mM LLOMe for 60min.
