# Supplementary material for: Connexin43 promotes exocytosis of damaged lysosomes through actin remodelling
Source: EMBO J. 2024 Jul 23;43(17):3627–49. doi: 10.1038/s44318-024-00177-3 (PMC11377567; doi:10.1038/s44318-024-00177-3)
Supplement: Supplementary file 4 — Movie EV2 [file 44318_2024_177_MOESM4_ESM.zip › Movie EV2 legend.docx]

Movie EV2 - Cx43 colocalizes with actin after LLOME treatment.

Confocal z-stack image series of HEK293A cells transiently transfected with GFP-Cx43 and mCherry-LifeAct, to visualize actin cytoskeleton, and treated for 60min with LLOMe.
