# Supplementary material for: Connexin43 promotes exocytosis of damaged lysosomes through actin remodelling
Source: EMBO J. 2024 Jul 23;43(17):3627–49. doi: 10.1038/s44318-024-00177-3 (PMC11377567; doi:10.1038/s44318-024-00177-3)
Supplement: Supplementary file 5 — Source data Fig. 1 [file 44318_2024_177_MOESM5_ESM.zip › Figure 1/1E/Western LAMP1/Western Lamp1 README.docx]

To obtain the Lamp1 “Biotin at PM pull down” source data, during the acquisition of the image, the part of the membrane containing the input samples was covered so the signal of the biotinylated samples was more easily visible.

This image was then flipped horizontally so that the treatments appeared in the same order as in the “Input” panel.
