# Supplementary material for: Connexin43 promotes exocytosis of damaged lysosomes through actin remodelling
Source: EMBO J. 2024 Jul 23;43(17):3627–49. doi: 10.1038/s44318-024-00177-3 (PMC11377567; doi:10.1038/s44318-024-00177-3)
Supplement: Supplementary file 5 — Source data Fig. 1 [file 44318_2024_177_MOESM5_ESM.zip › Figure 1/1E/Western Gal3/Western Gal3 README.docx]

To build the Gal3 “Biotin at PM pull down” panel, the original image was flipped horizontally so that the treatments appeared in the same order as in the “Input” panel.
