# Supplementary figures and images for: Connexin43 promotes exocytosis of damaged lysosomes through actin remodelling
Source: EMBO J. 2024 Jul 23;43(17):3627–49. doi: 10.1038/s44318-024-00177-3 (PMC11377567; doi:10.1038/s44318-024-00177-3)

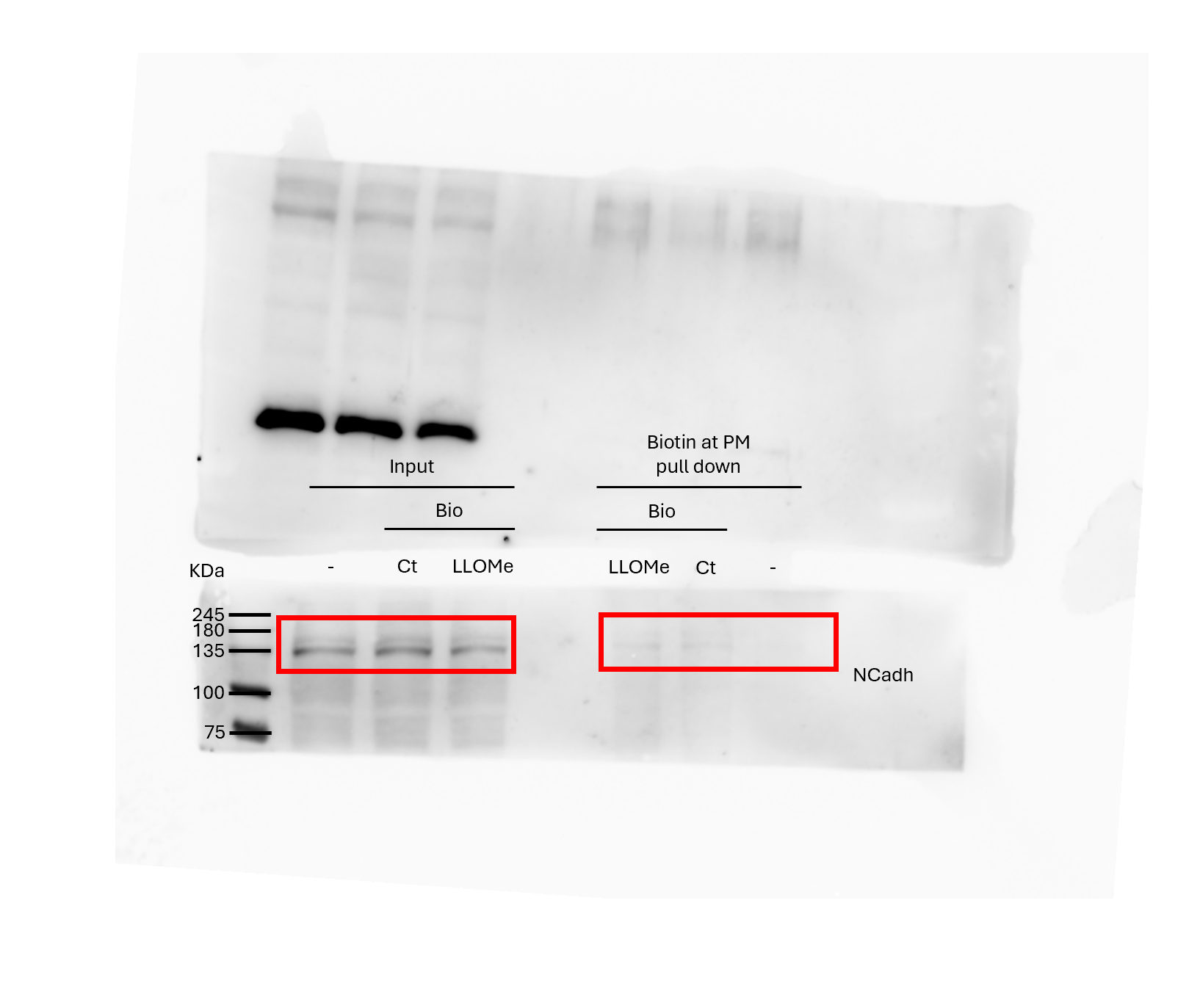

Supplement: Supplementary file 5 — Source data Fig. 1 [file 44318_2024_177_MOESM5_ESM.zip › Figure 1/1E/Western NCadh/Western NCadh.tif]

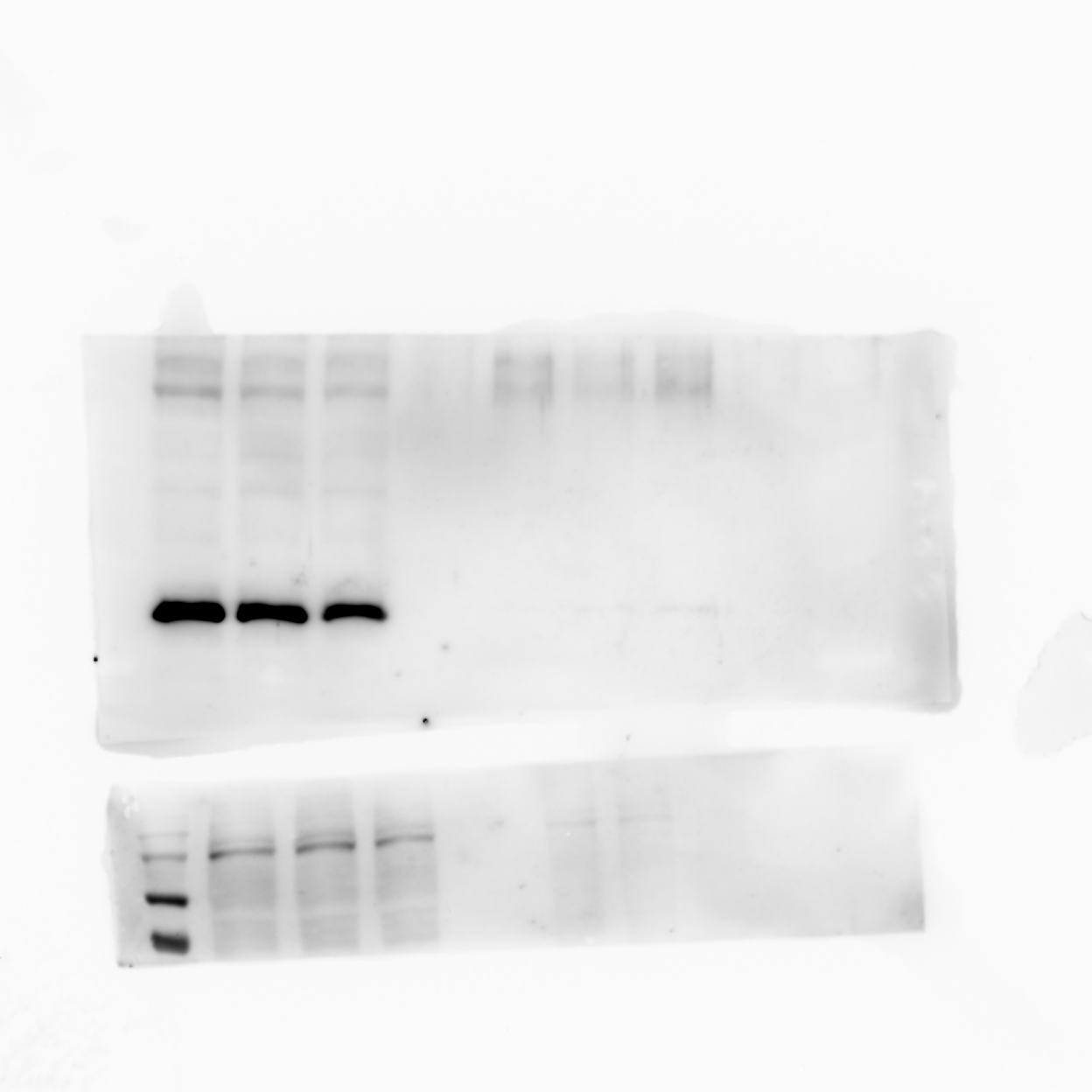

Supplement: Supplementary file 5 — Source data Fig. 1 [file 44318_2024_177_MOESM5_ESM.zip › Figure 1/1E/Western NCadh/Western NCadh 20220121_123950-08_Ch.tif]

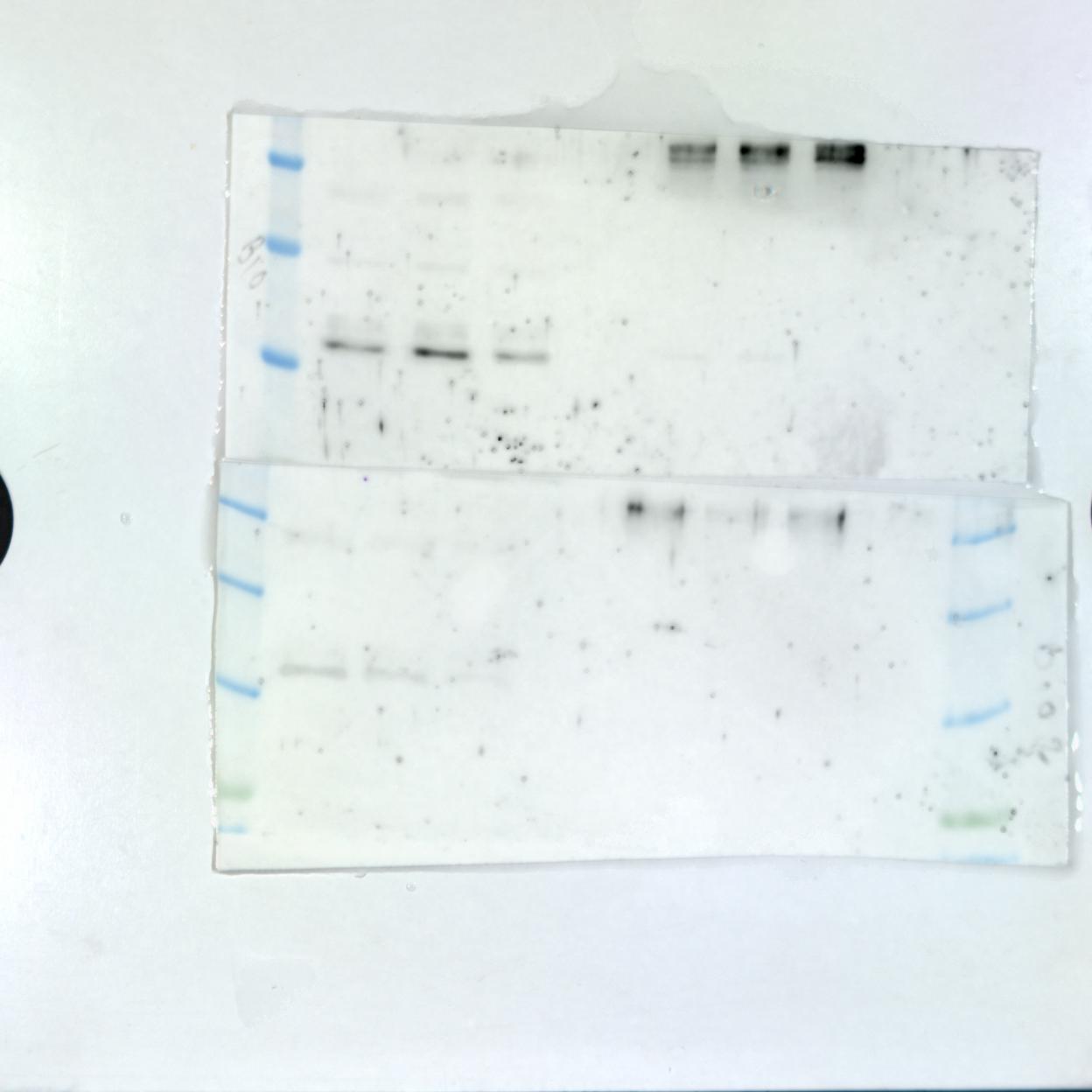

Supplement: Supplementary file 5 — Source data Fig. 1 [file 44318_2024_177_MOESM5_ESM.zip › Figure 1/1E/Western hnRNPA2B1/bc.nucl.2 20220125_162050_Ch+Marker.jpg]

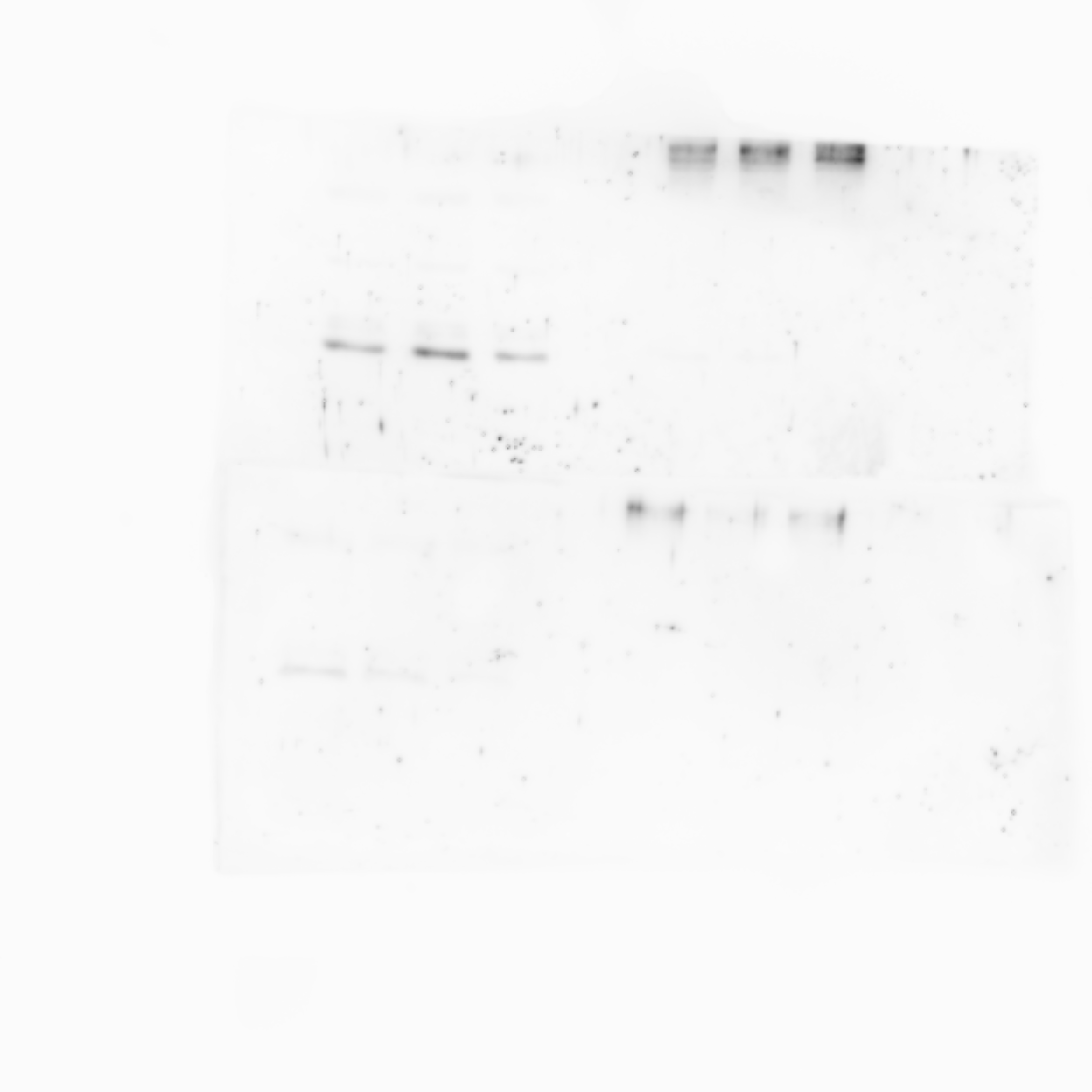

Supplement: Supplementary file 5 — Source data Fig. 1 [file 44318_2024_177_MOESM5_ESM.zip › Figure 1/1E/Western hnRNPA2B1/Western hnRNPA2B1 2 20220125_162050_Ch.tif]

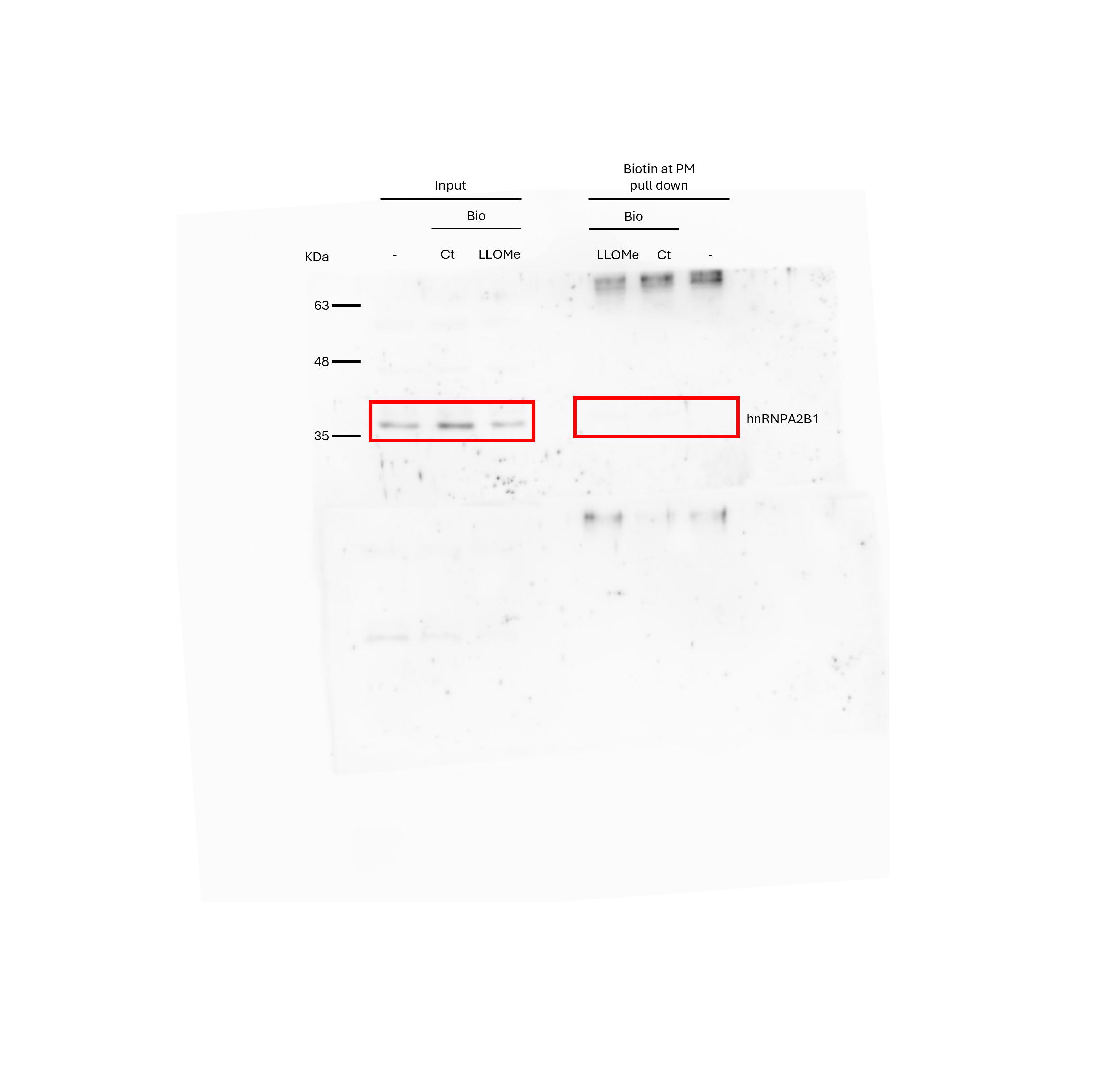

Supplement: Supplementary file 5 — Source data Fig. 1 [file 44318_2024_177_MOESM5_ESM.zip › Figure 1/1E/Western hnRNPA2B1/Western hnRNPA2B1.tif]

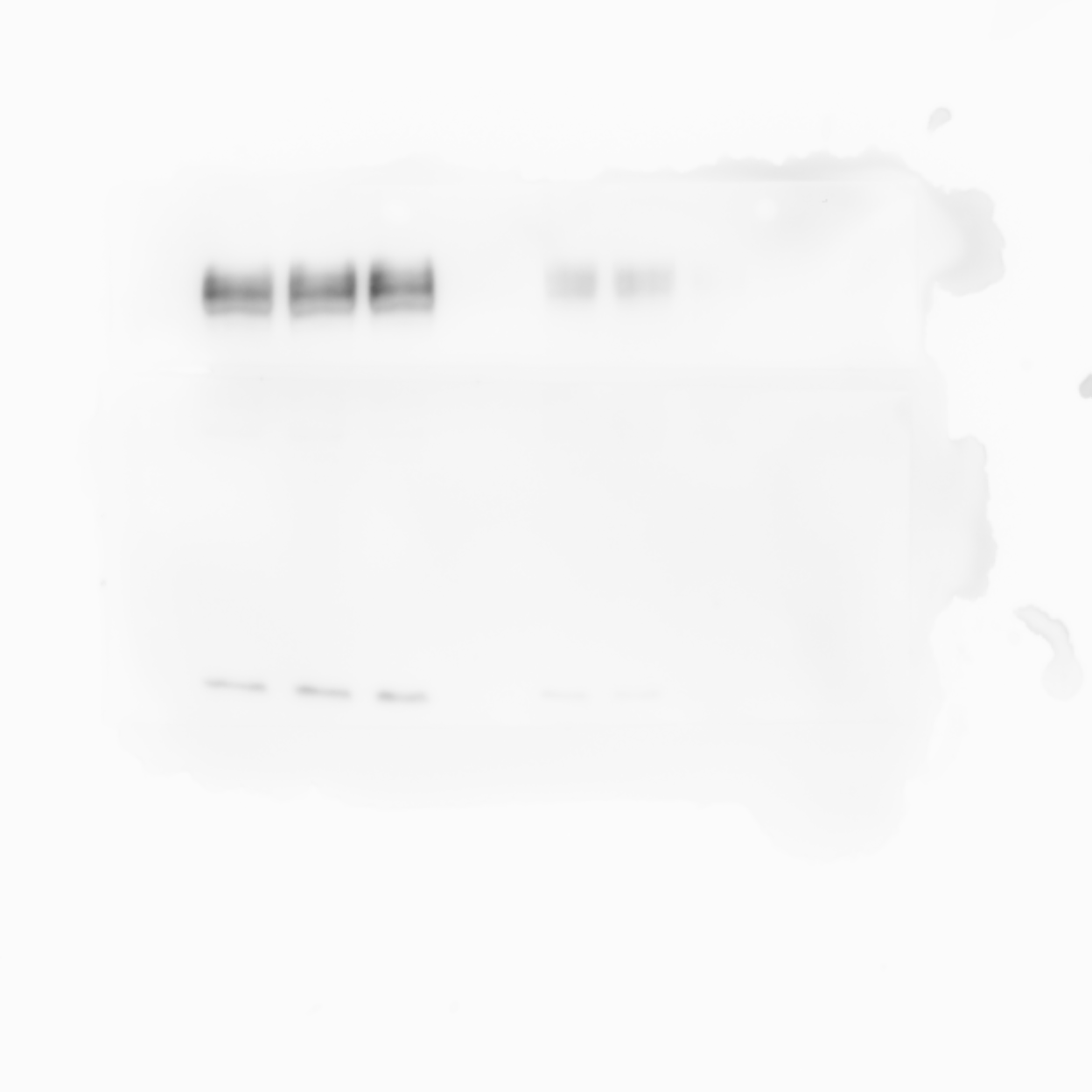

Supplement: Supplementary file 5 — Source data Fig. 1 [file 44318_2024_177_MOESM5_ESM.zip › Figure 1/1E/Western LAMP1/Western LAMP1 20220119_120059_Ch.tif]

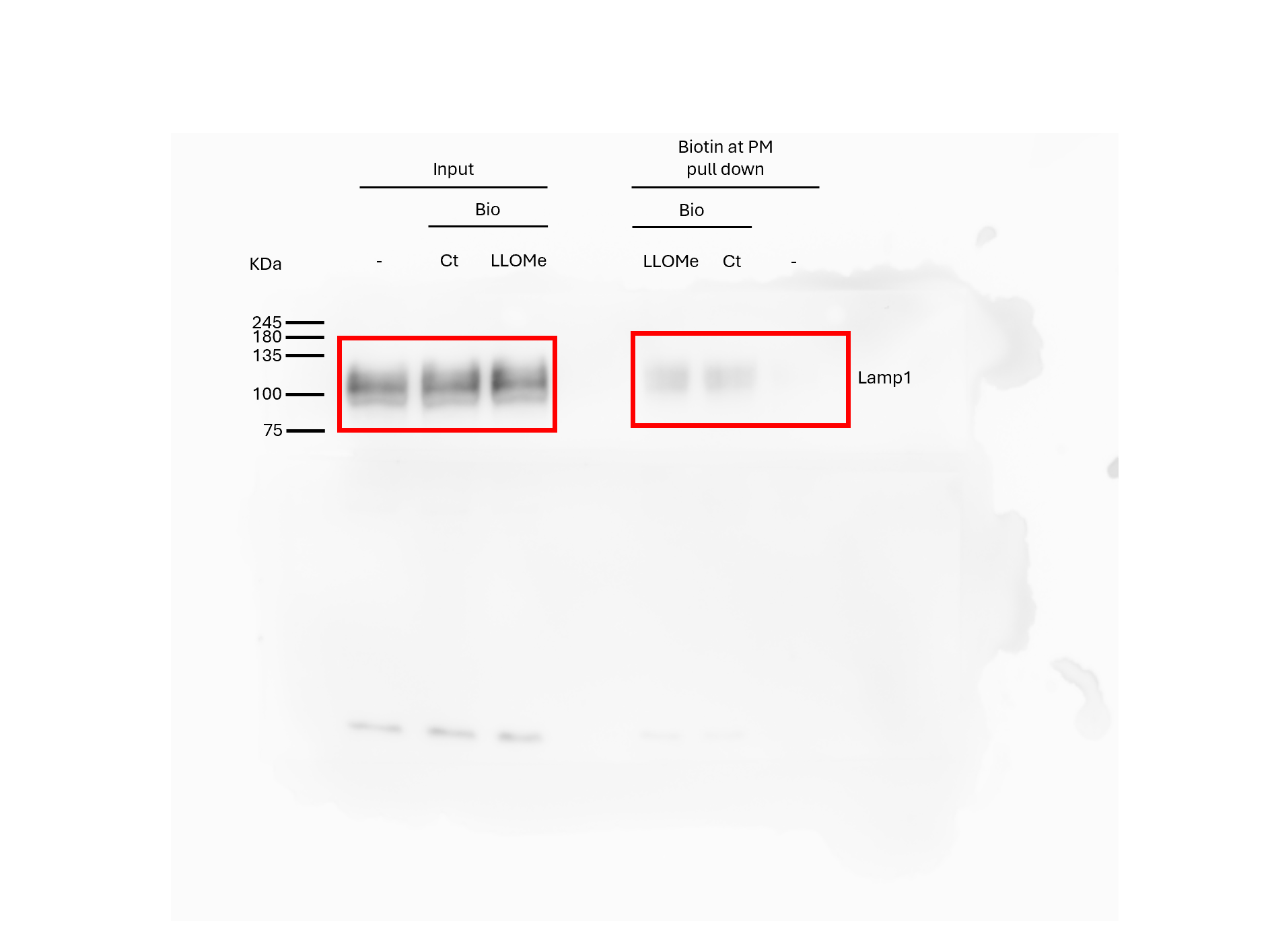

Supplement: Supplementary file 5 — Source data Fig. 1 [file 44318_2024_177_MOESM5_ESM.zip › Figure 1/1E/Western LAMP1/Western LAMP1.tif]

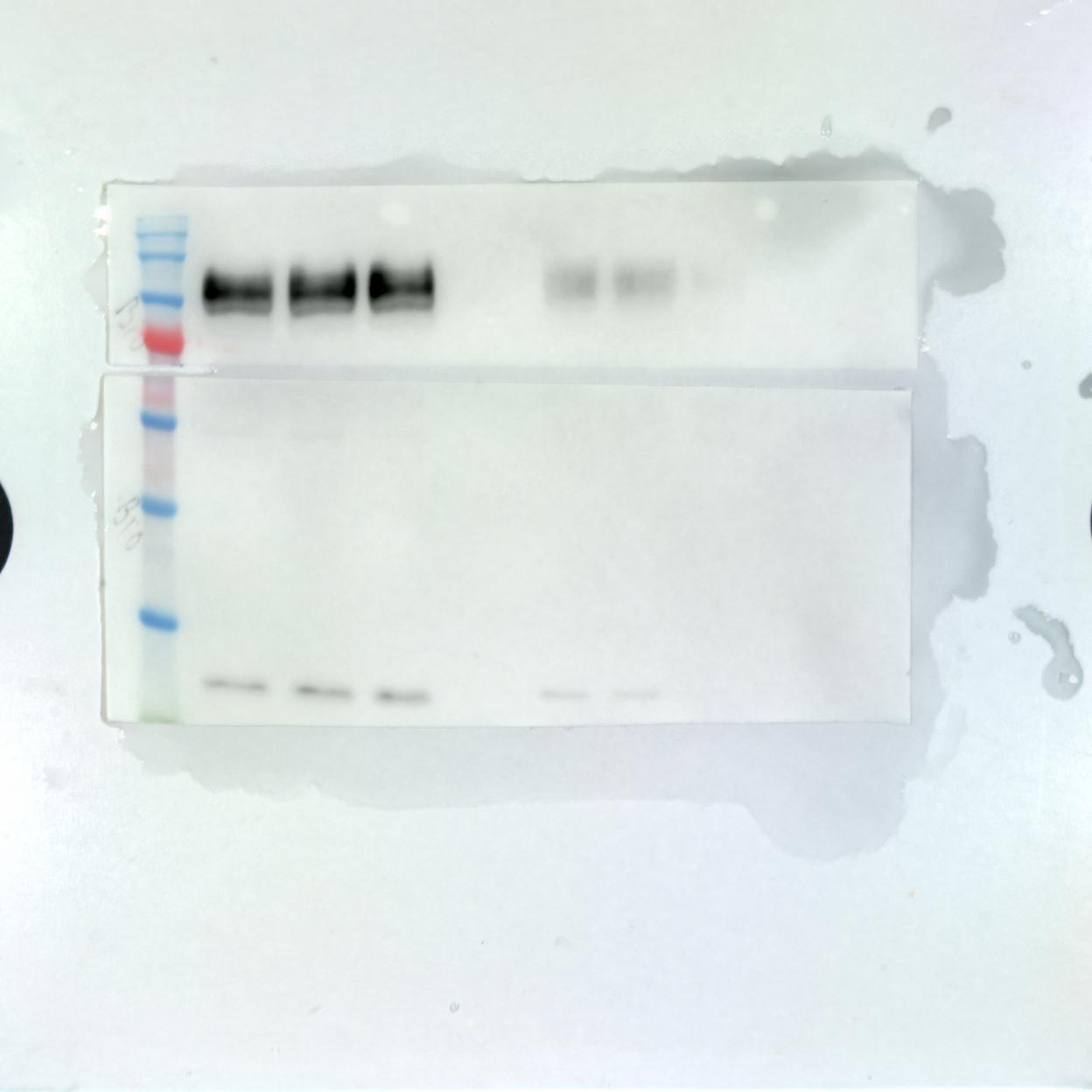

Supplement: Supplementary file 5 — Source data Fig. 1 [file 44318_2024_177_MOESM5_ESM.zip › Figure 1/1E/Western LAMP1/Western LAMP1 20220119_120059_Ch+Marker.jpg]

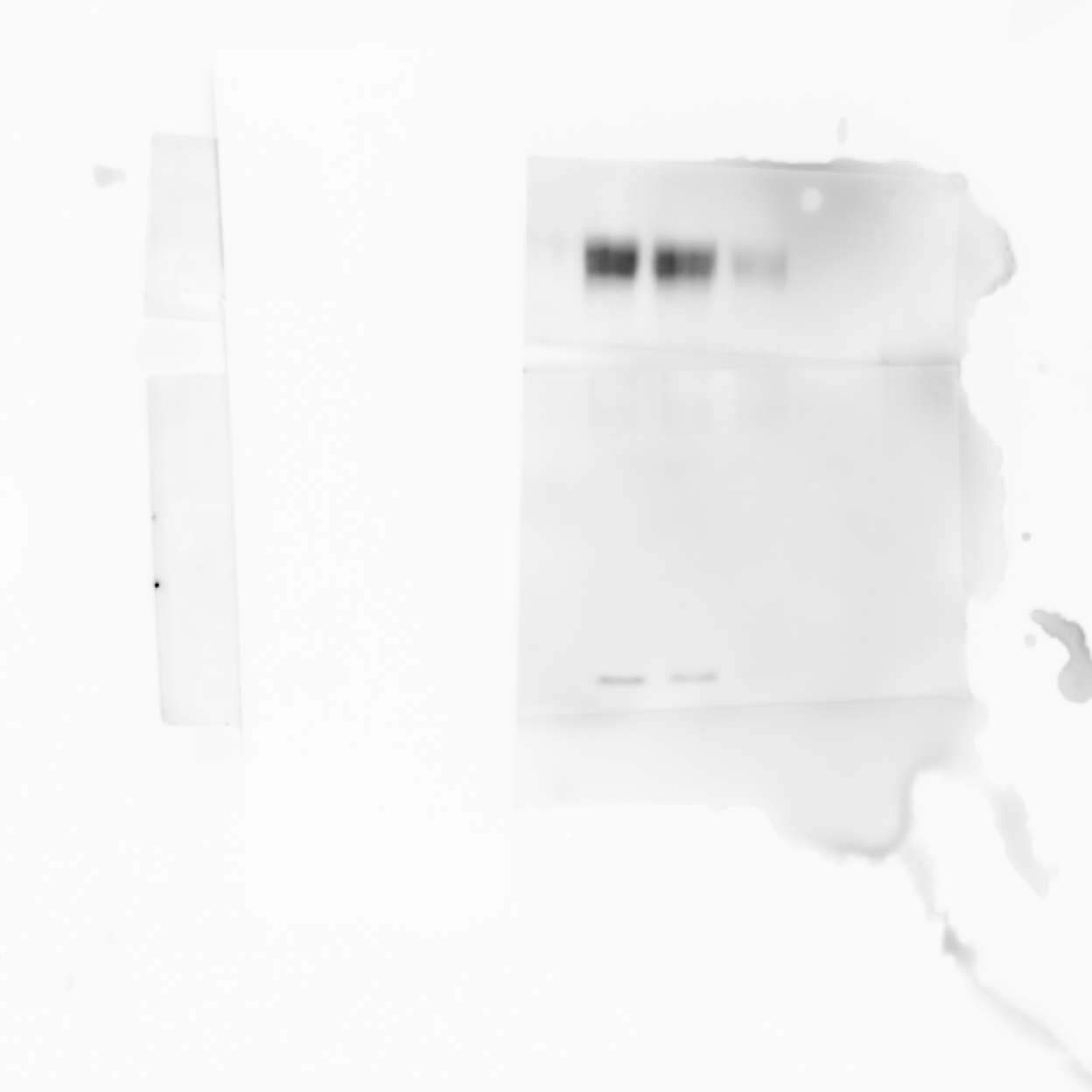

Supplement: Supplementary file 5 — Source data Fig. 1 [file 44318_2024_177_MOESM5_ESM.zip › Figure 1/1E/Western LAMP1/Western LAMP1 20220119_120850-05_Ch.tif]

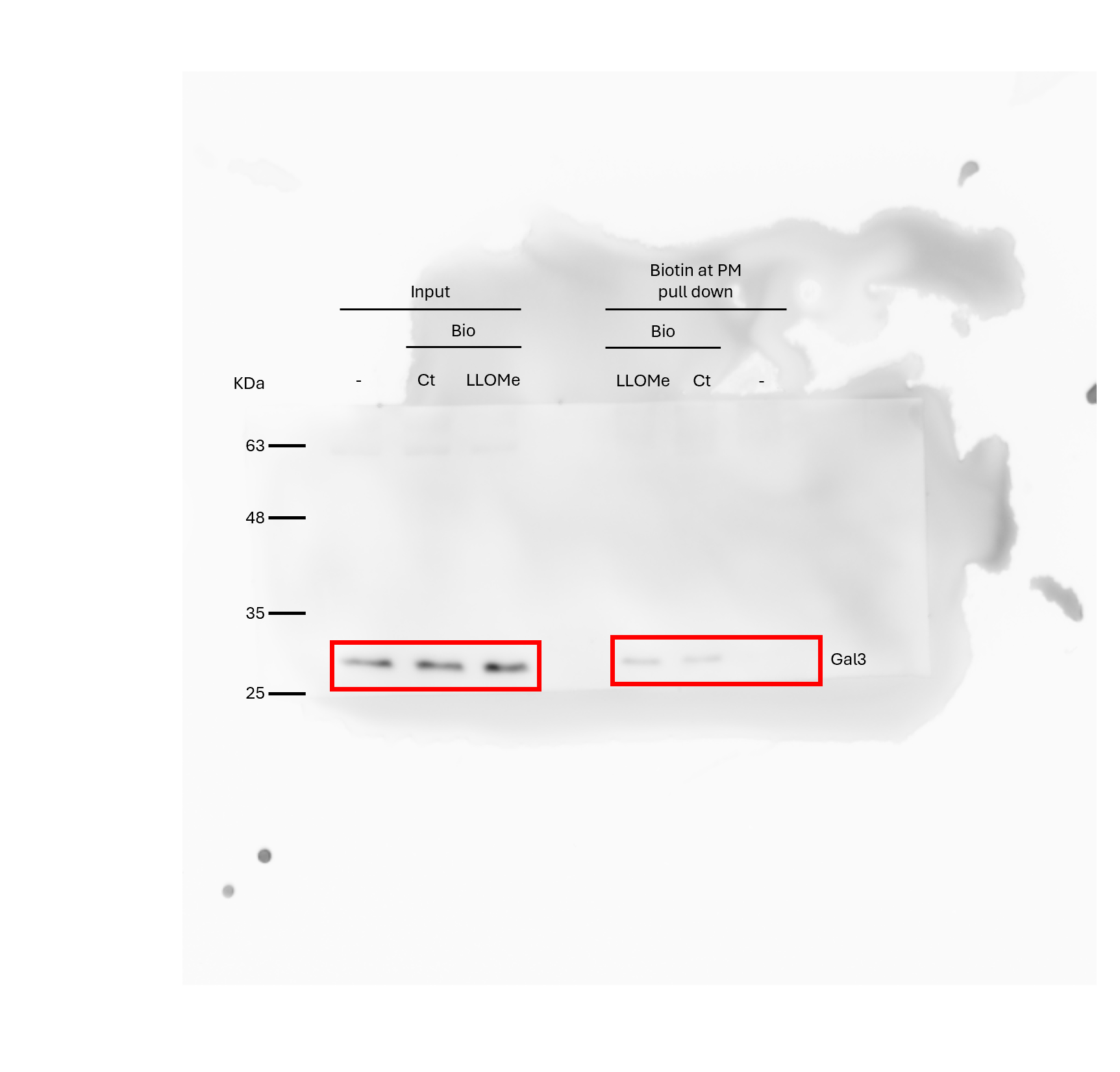

Supplement: Supplementary file 5 — Source data Fig. 1 [file 44318_2024_177_MOESM5_ESM.zip › Figure 1/1E/Western Gal3/Western Gal3.tif]

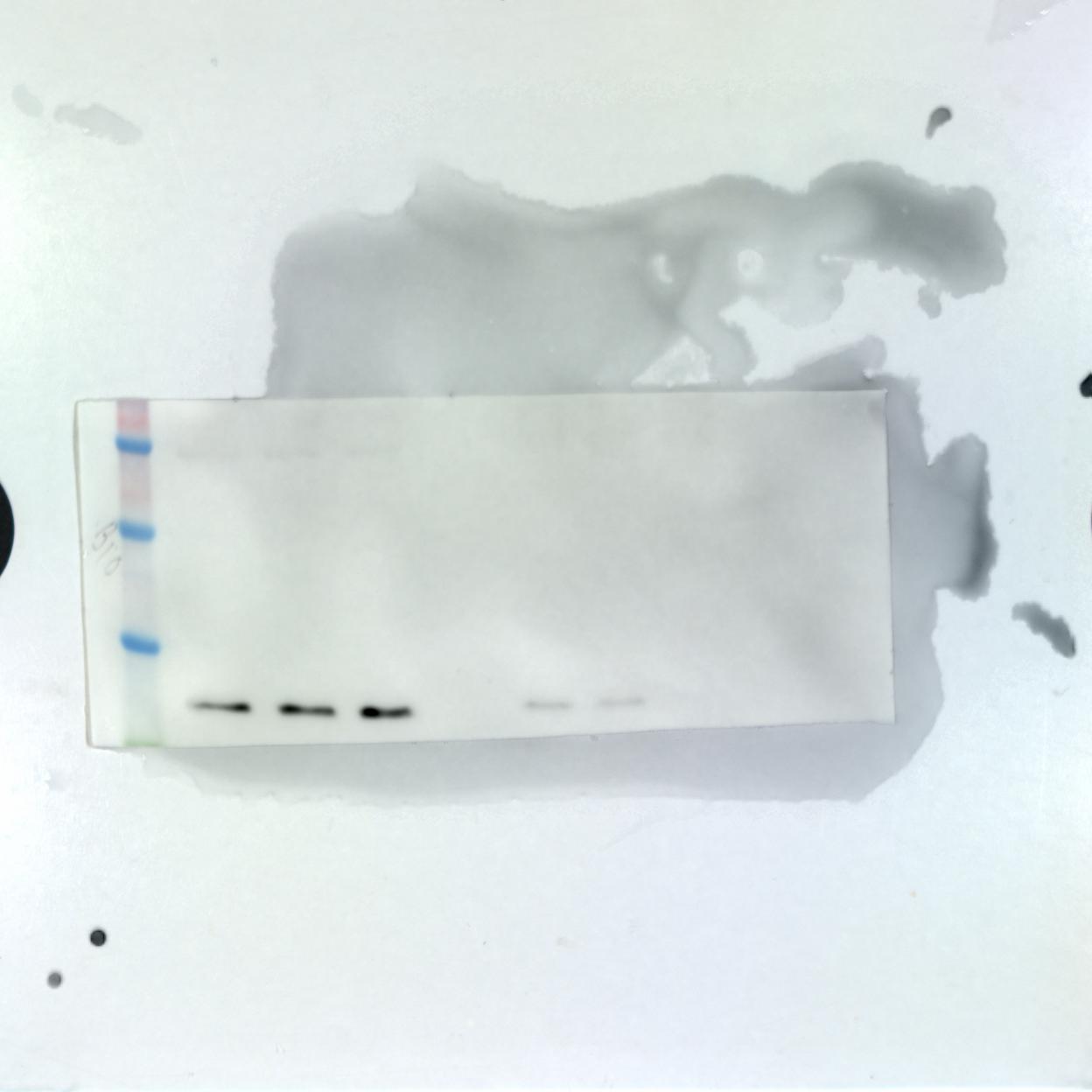

Supplement: Supplementary file 5 — Source data Fig. 1 [file 44318_2024_177_MOESM5_ESM.zip › Figure 1/1E/Western Gal3/Western Gal3 20220119_115404_Ch+Marker.jpg]

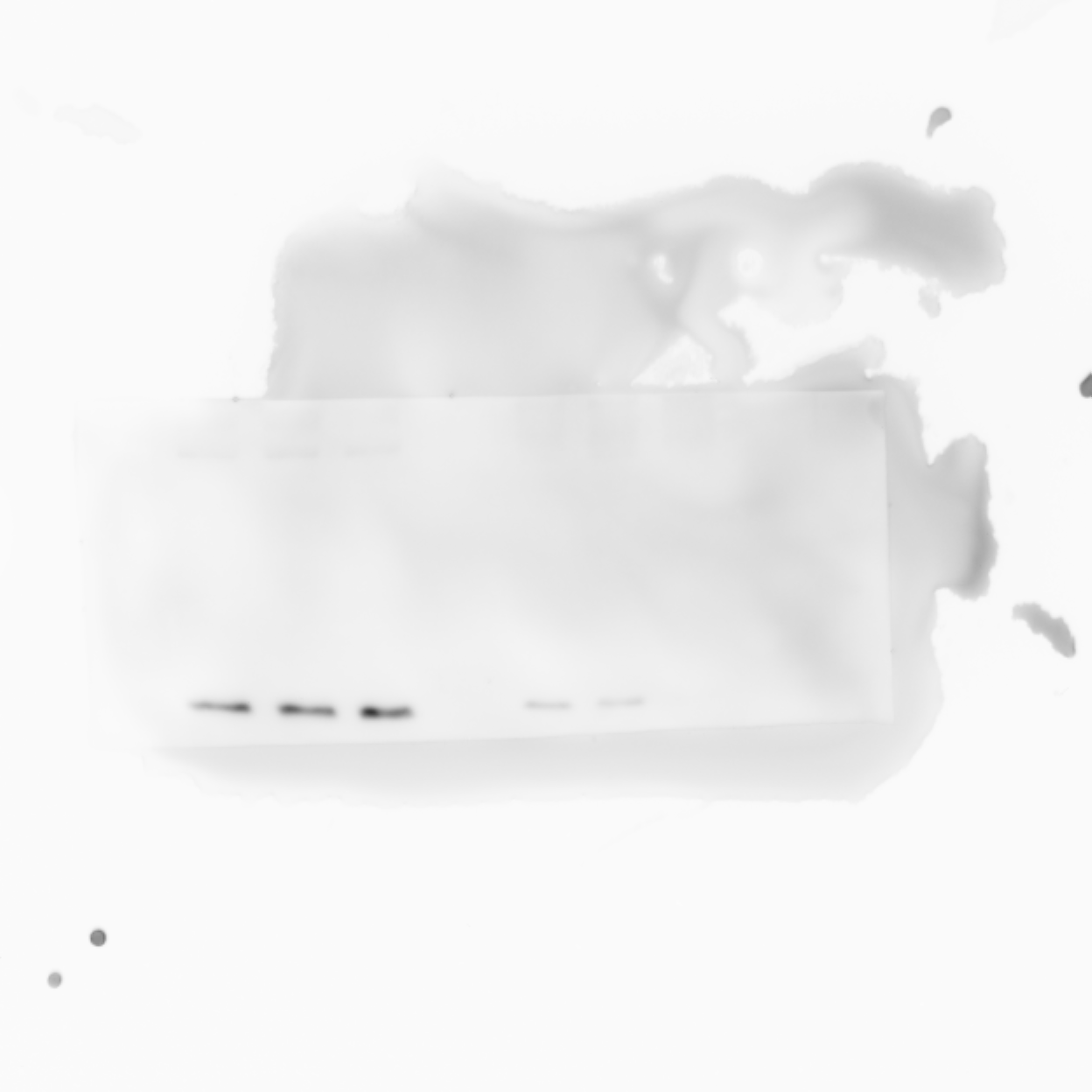

Supplement: Supplementary file 5 — Source data Fig. 1 [file 44318_2024_177_MOESM5_ESM.zip › Figure 1/1E/Western Gal3/Western Gal3 20220119_115404_Ch.tif]

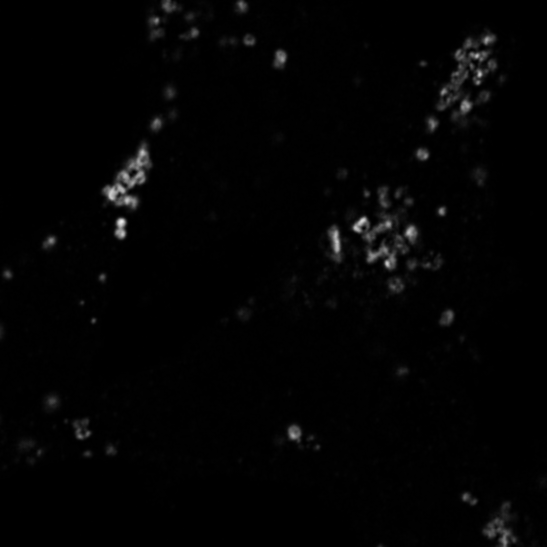

Supplement: Supplementary file 6 — Source data Fig. 2 [file 44318_2024_177_MOESM6_ESM.zip › Figure 2/2H/Fluorescence Microscopy HEK293A LLOMe LAMP.tif]

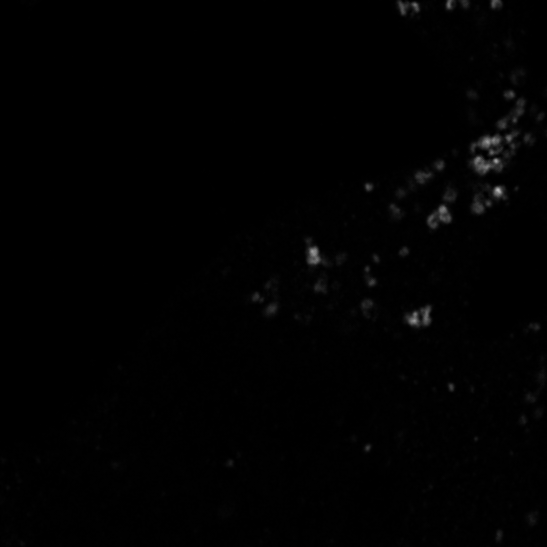

Supplement: Supplementary file 6 — Source data Fig. 2 [file 44318_2024_177_MOESM6_ESM.zip › Figure 2/2H/Microscopy HEK293Cx43+ LLOMe LAMP.tif]

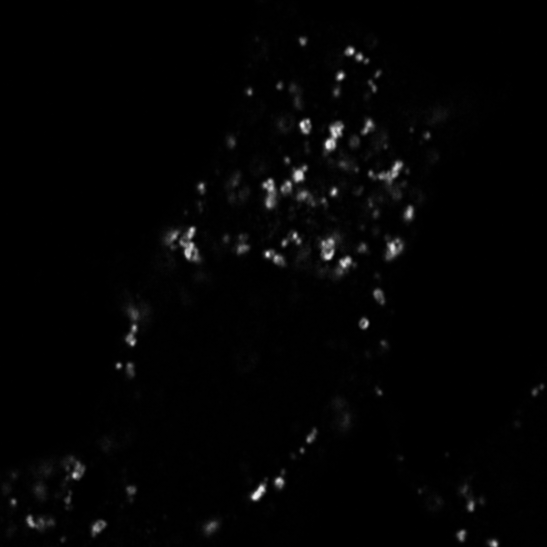

Supplement: Supplementary file 6 — Source data Fig. 2 [file 44318_2024_177_MOESM6_ESM.zip › Figure 2/2H/Fluorescence Microscopy HEK293A Ct LAMP.tif]

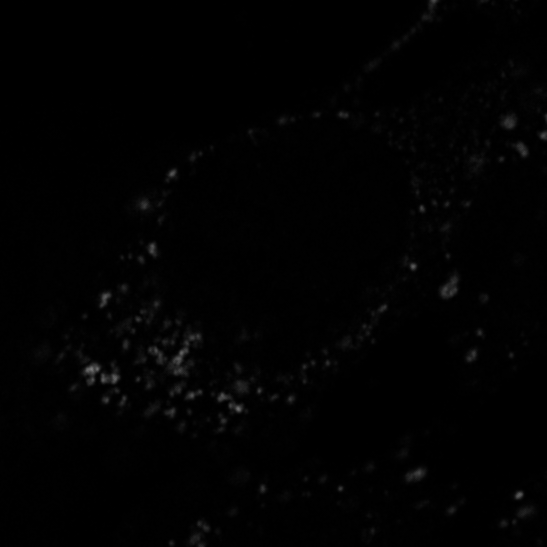

Supplement: Supplementary file 6 — Source data Fig. 2 [file 44318_2024_177_MOESM6_ESM.zip › Figure 2/2H/Fluorescence Microscopy HEK293Cx43+ Ct LAMP.tif]

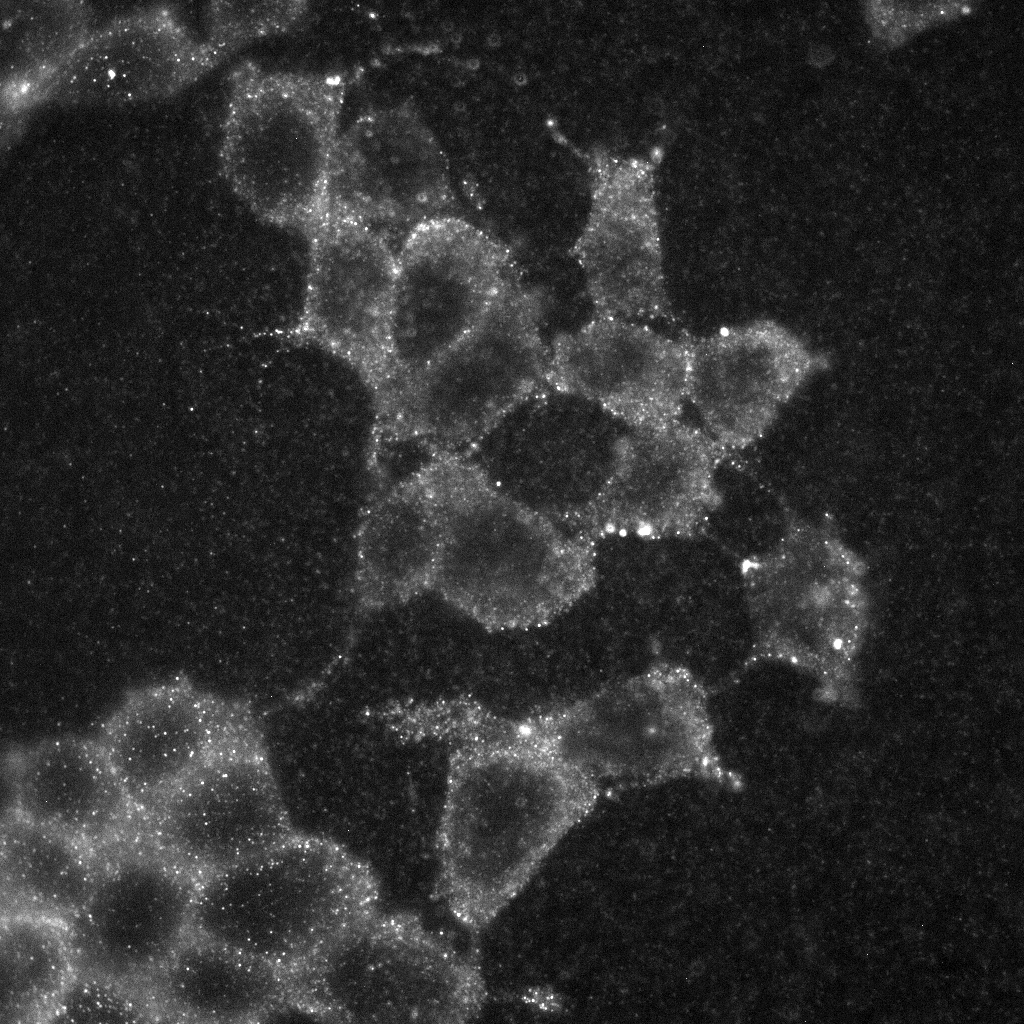

Supplement: Supplementary file 6 — Source data Fig. 2 [file 44318_2024_177_MOESM6_ESM.zip › Figure 2/2B/Fluorescence Microscopy HEK293A ion LAMP at PM.tif]

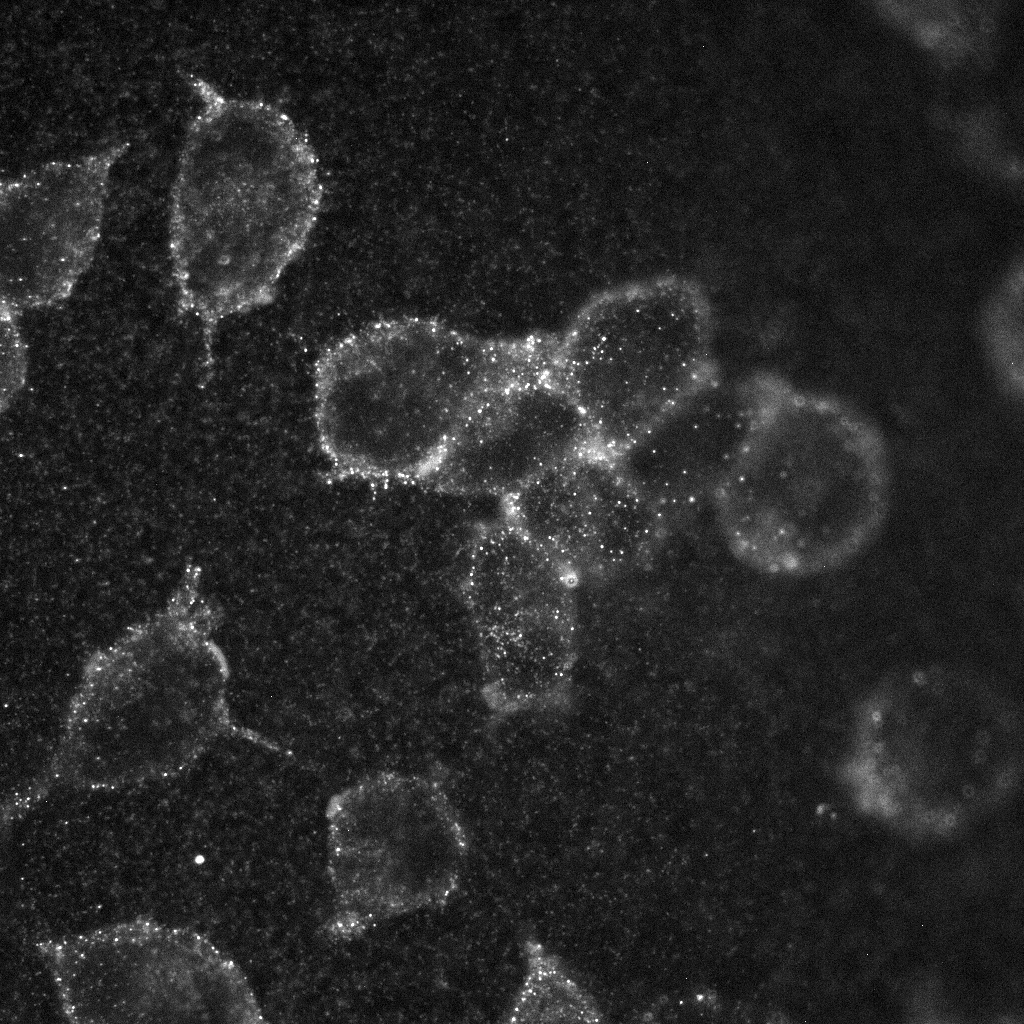

Supplement: Supplementary file 6 — Source data Fig. 2 [file 44318_2024_177_MOESM6_ESM.zip › Figure 2/2B/Fluorescence Microscopy HEK293A LLOMe LAMP at PM.tif]

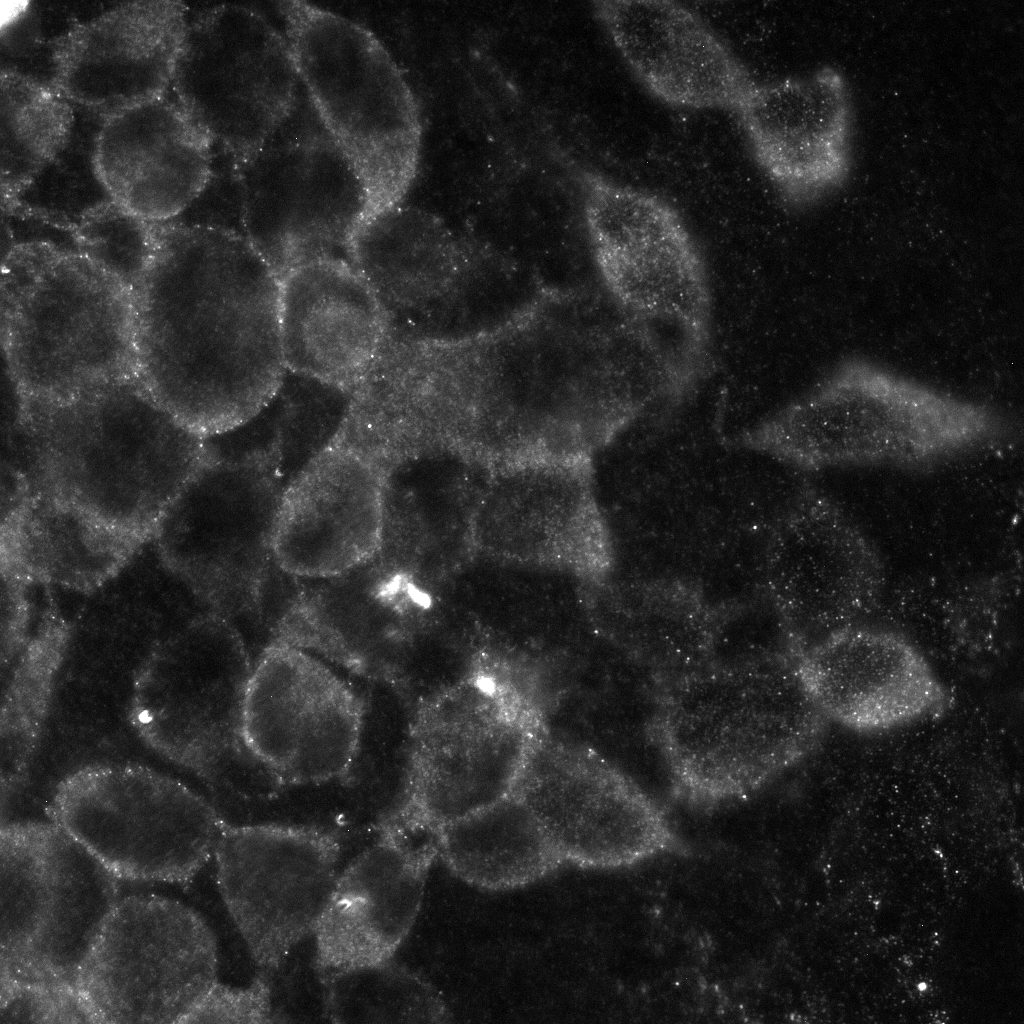

Supplement: Supplementary file 6 — Source data Fig. 2 [file 44318_2024_177_MOESM6_ESM.zip › Figure 2/2B/Fluorescence Microscopy HEK293Cx43+ LLOMe LAMP at PM.tif]

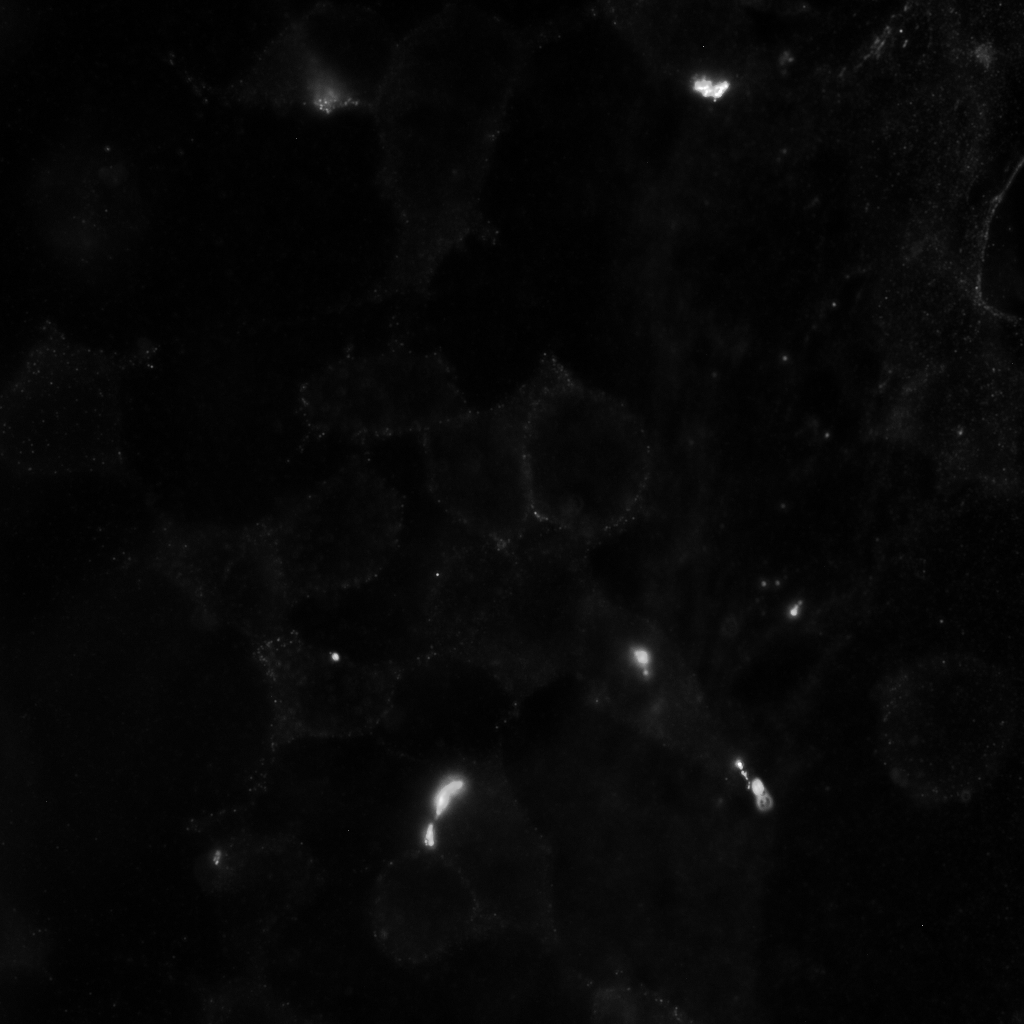

Supplement: Supplementary file 6 — Source data Fig. 2 [file 44318_2024_177_MOESM6_ESM.zip › Figure 2/2B/Fluorescence Microscopy HEK293Cx43+ ion LAMP at PM.tif]

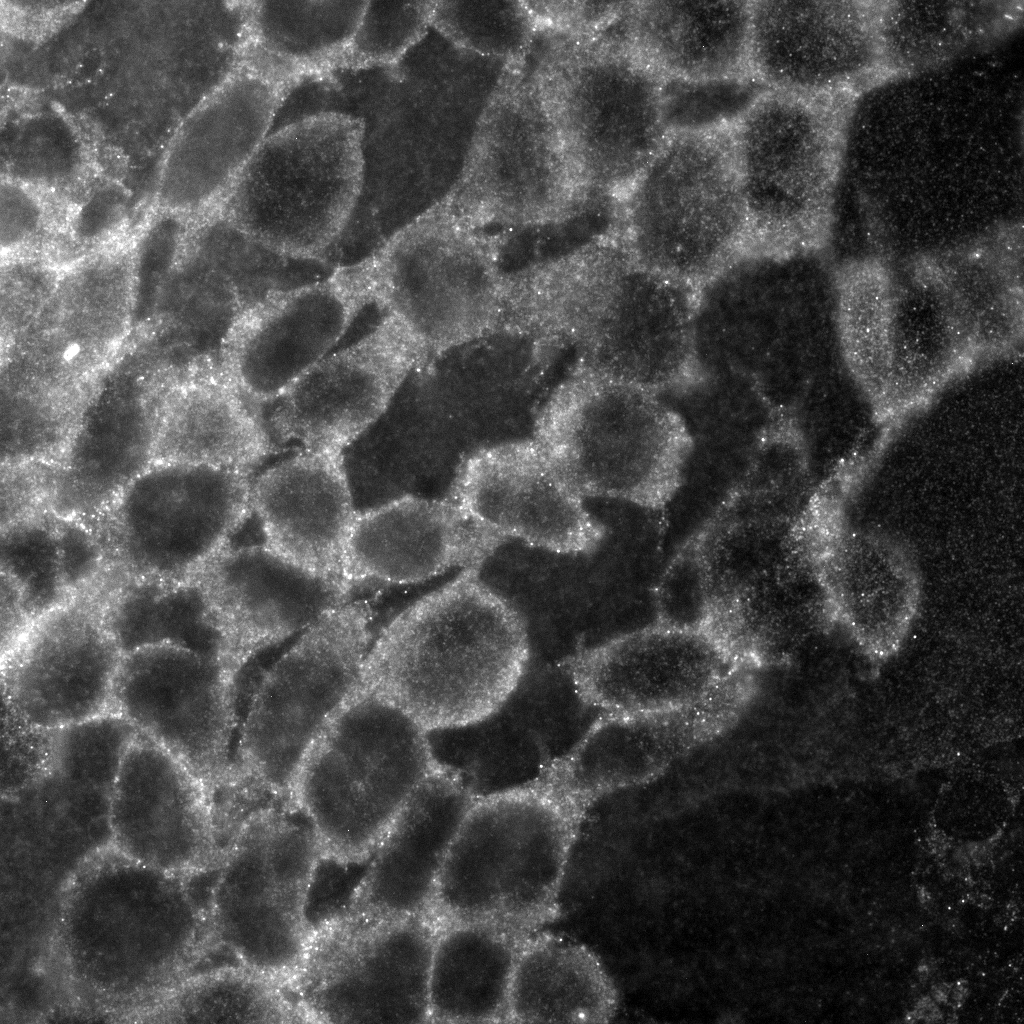

Supplement: Supplementary file 6 — Source data Fig. 2 [file 44318_2024_177_MOESM6_ESM.zip › Figure 2/2B/Fluorescence Microscopy HEK293Cx43+ Ct LAMP at PM.tif]

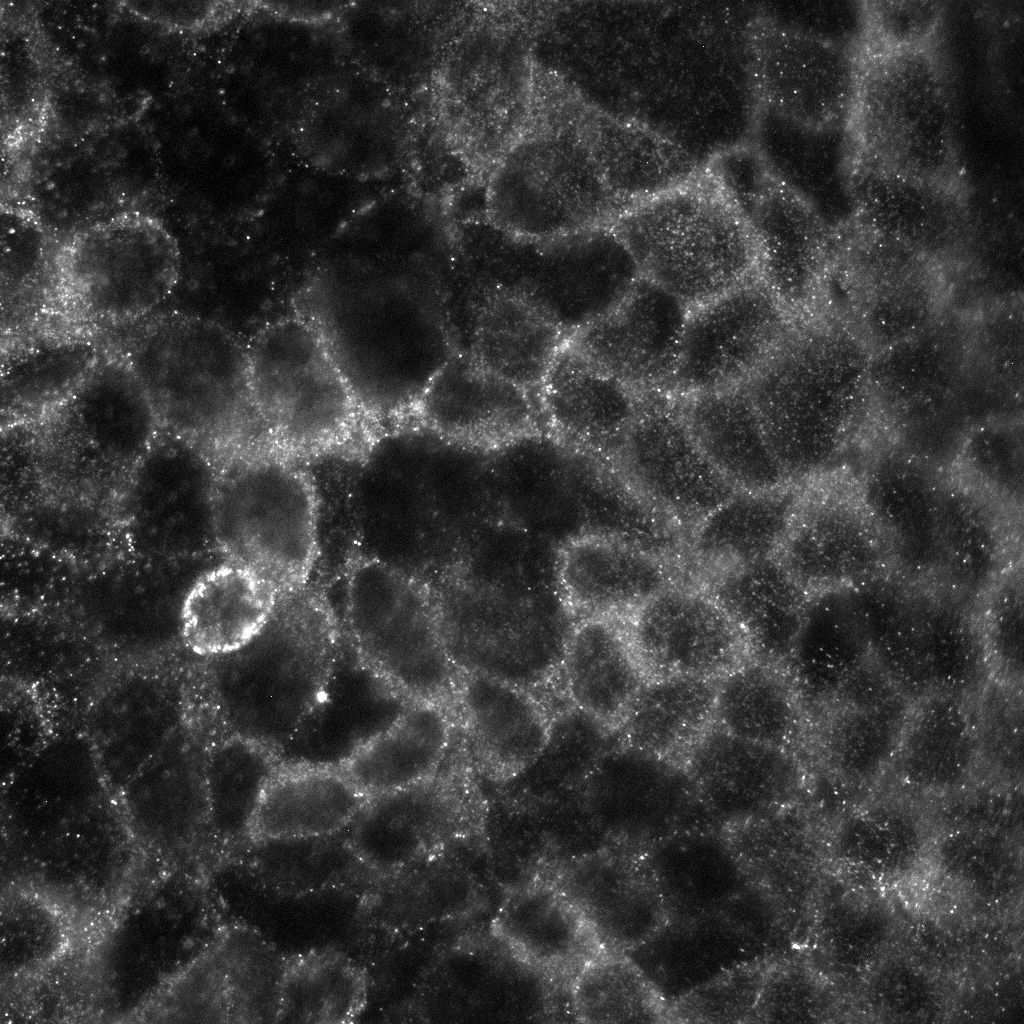

Supplement: Supplementary file 6 — Source data Fig. 2 [file 44318_2024_177_MOESM6_ESM.zip › Figure 2/2B/Fluorescence Microscopy HEK293A Ct LAMP at PM.tif]

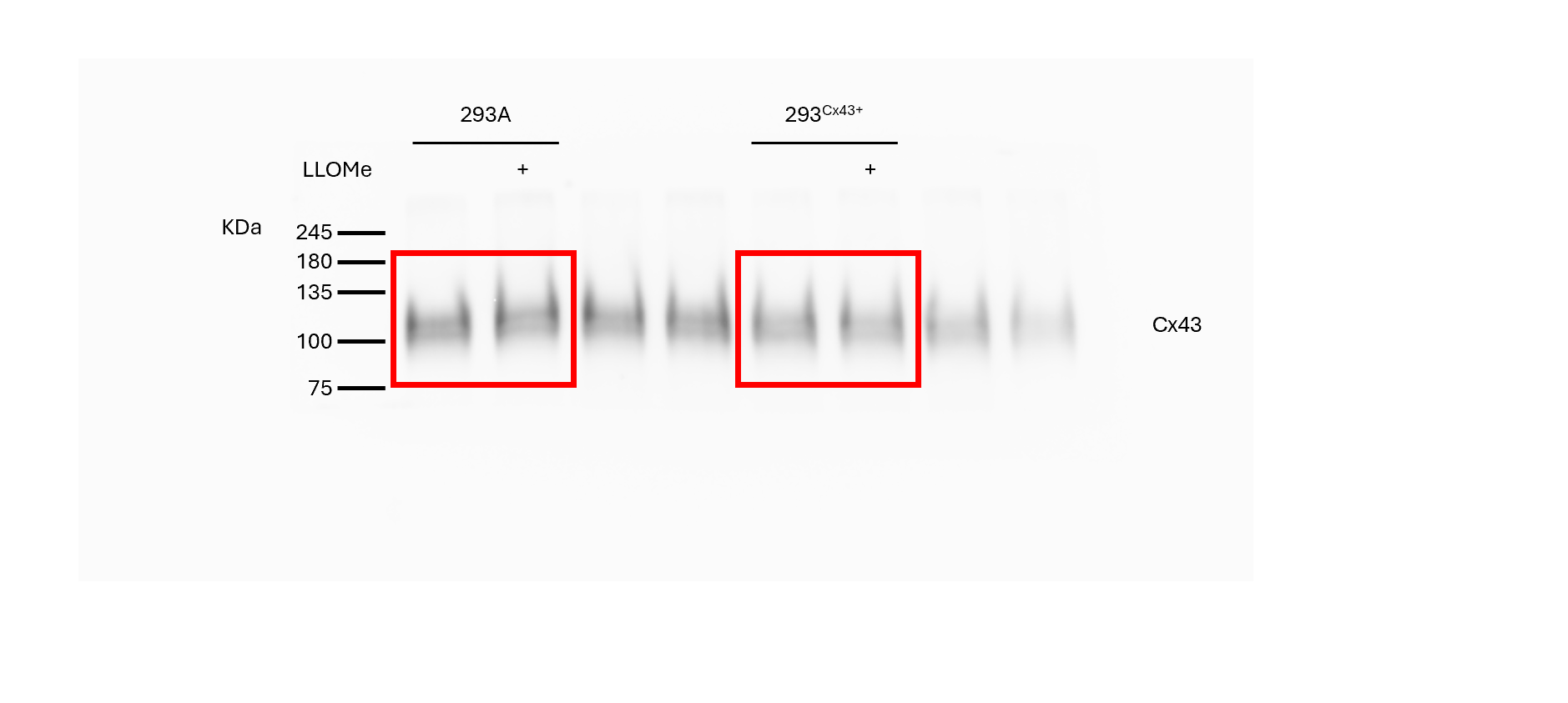

Supplement: Supplementary file 6 — Source data Fig. 2 [file 44318_2024_177_MOESM6_ESM.zip › Figure 2/2K/Western LAMP1 Cellular Extract/Western LAMP1 Cellular Extracts.tif]

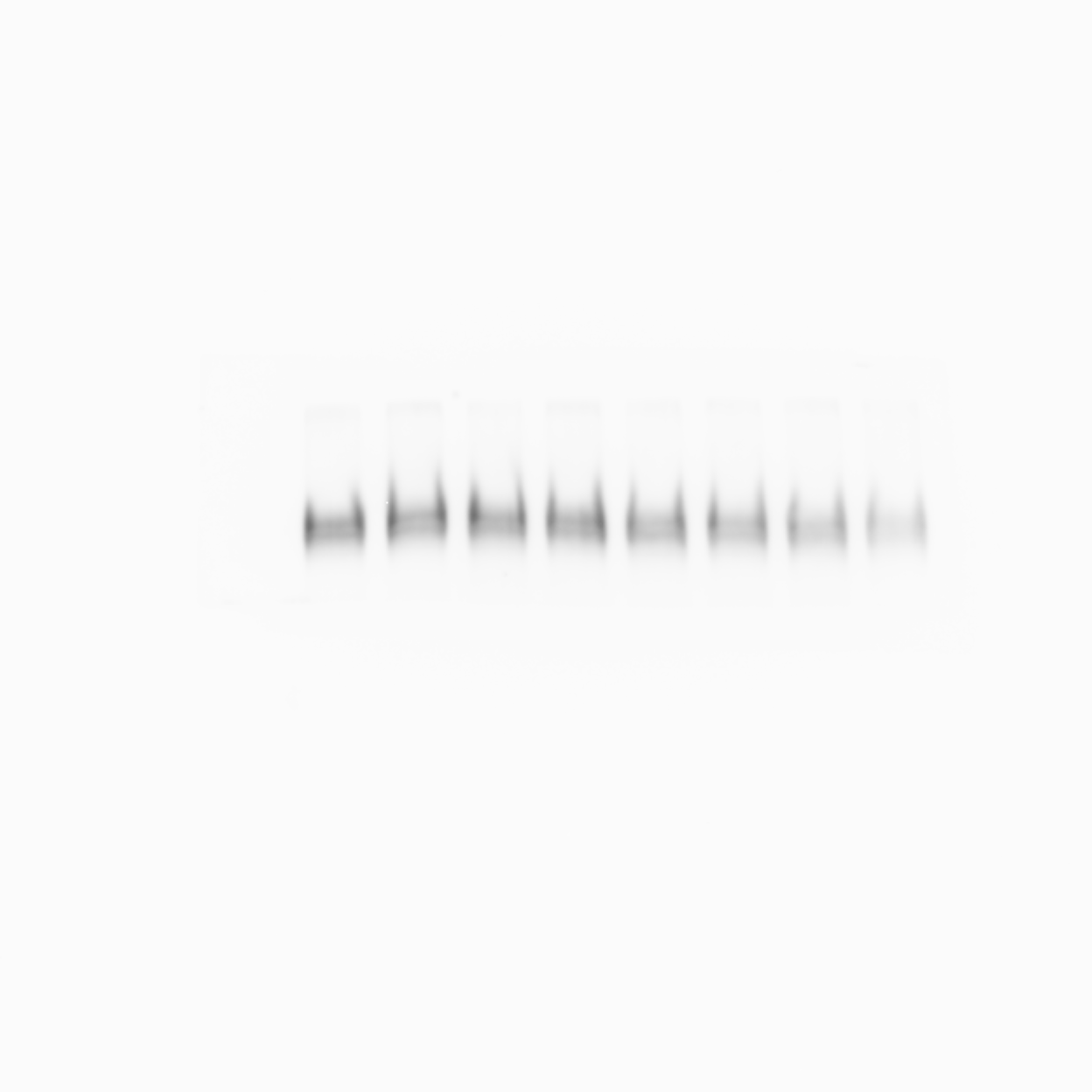

Supplement: Supplementary file 6 — Source data Fig. 2 [file 44318_2024_177_MOESM6_ESM.zip › Figure 2/2K/Western LAMP1 Cellular Extract/Western LAMP1 Cellular Extracts 20220203_131814_Ch.tif]

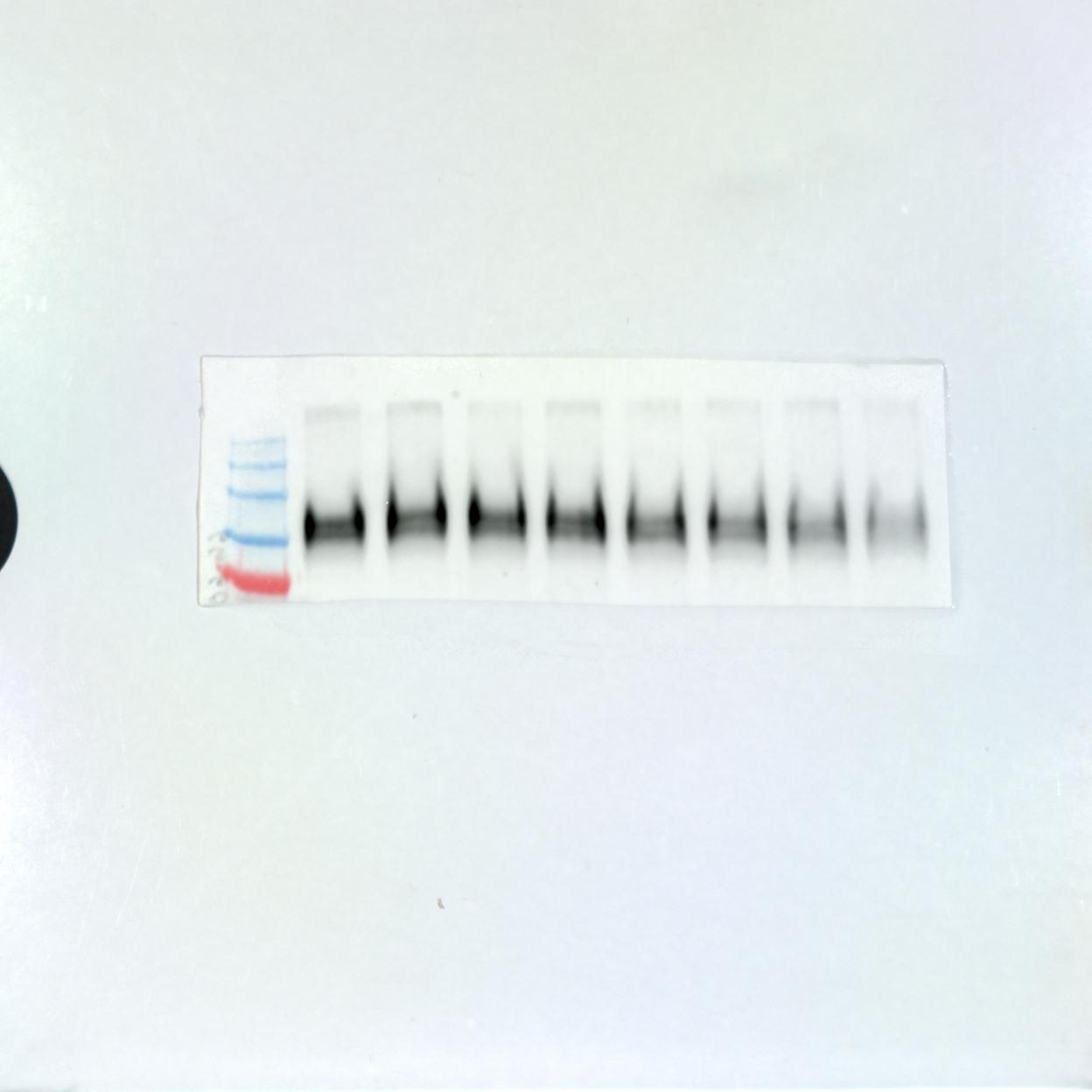

Supplement: Supplementary file 6 — Source data Fig. 2 [file 44318_2024_177_MOESM6_ESM.zip › Figure 2/2K/Western LAMP1 Cellular Extract/Western LAMP1 Cellular Extracts 20220203_131814_Ch+Marker.jpg]

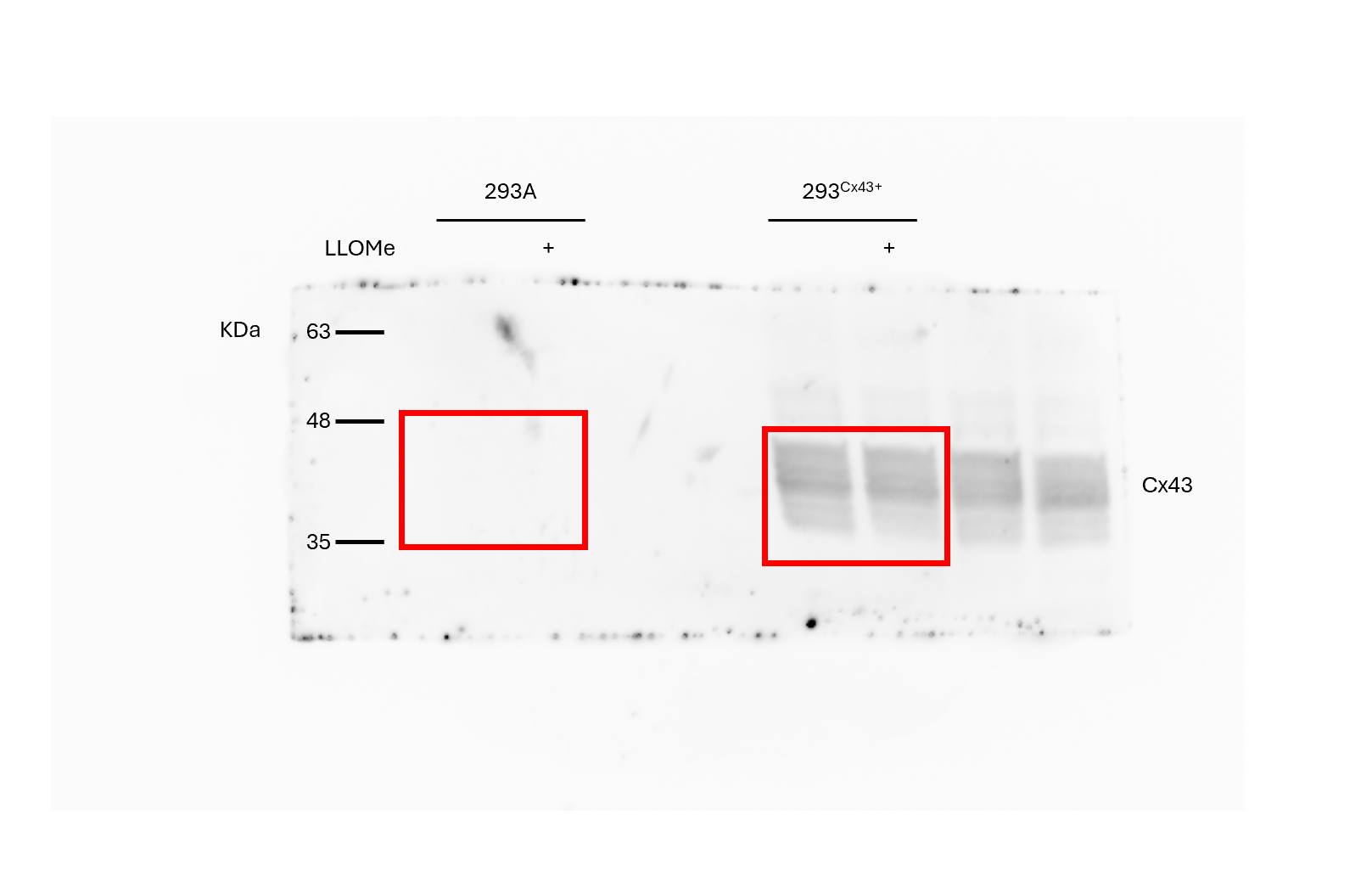

Supplement: Supplementary file 6 — Source data Fig. 2 [file 44318_2024_177_MOESM6_ESM.zip › Figure 2/2K/Western Cx43 Cellular Extract/Western Cx43 Cellular Extracts.tif]

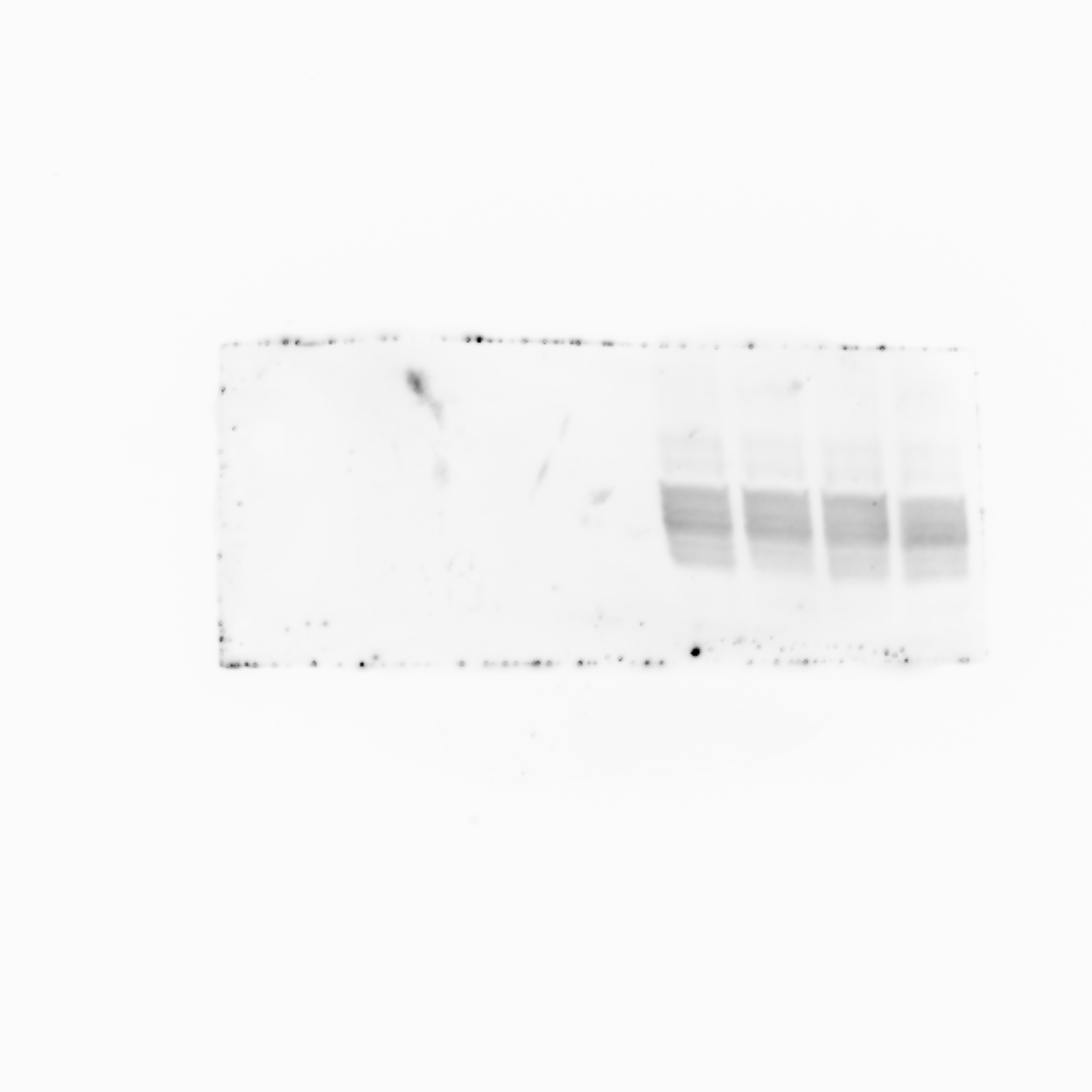

Supplement: Supplementary file 6 — Source data Fig. 2 [file 44318_2024_177_MOESM6_ESM.zip › Figure 2/2K/Western Cx43 Cellular Extract/Western Cx43 Cellular Extract 20220203_131432_Ch.tif]

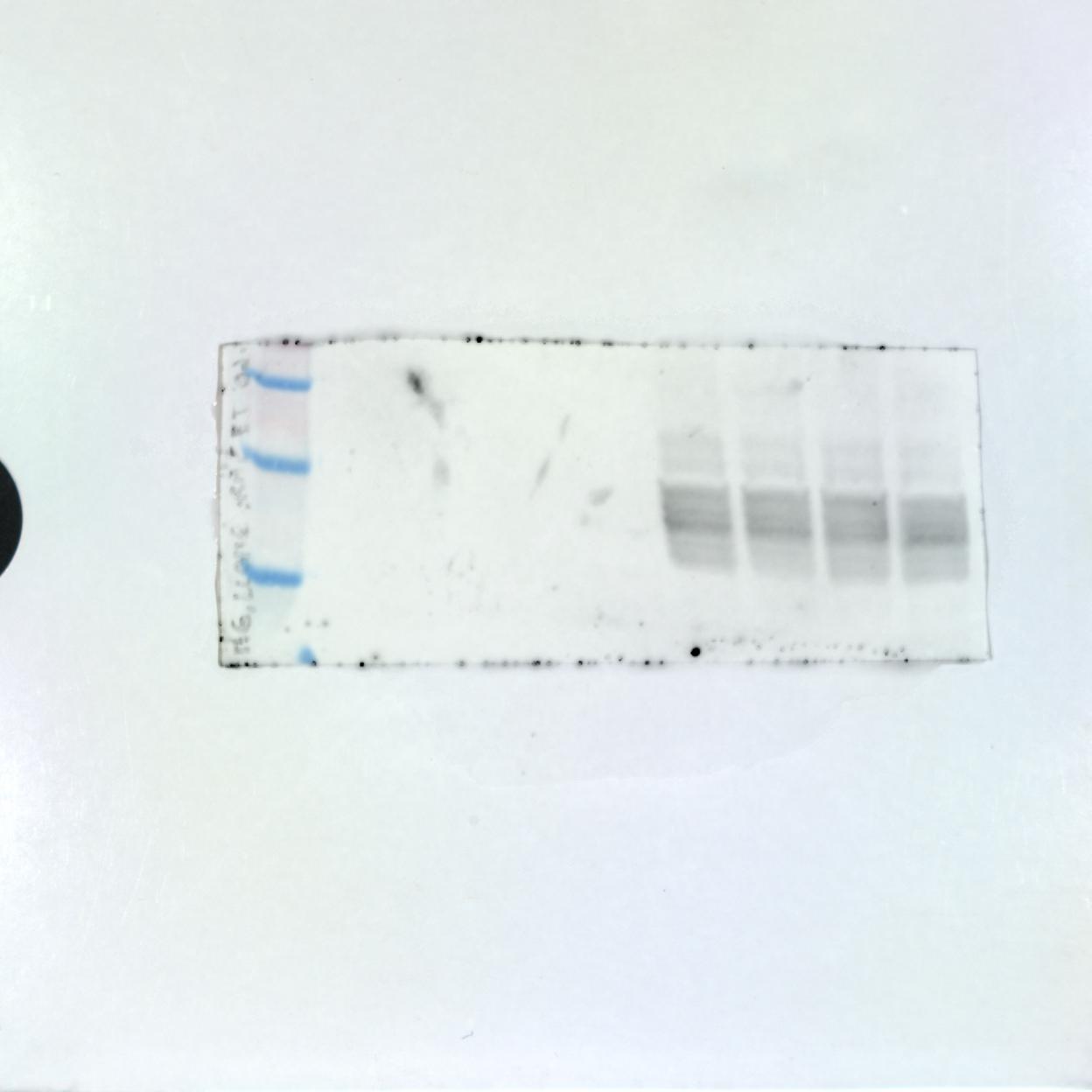

Supplement: Supplementary file 6 — Source data Fig. 2 [file 44318_2024_177_MOESM6_ESM.zip › Figure 2/2K/Western Cx43 Cellular Extract/Western Cx43 Cellular Extract 20220203_131432_Ch+Marker.jpg]

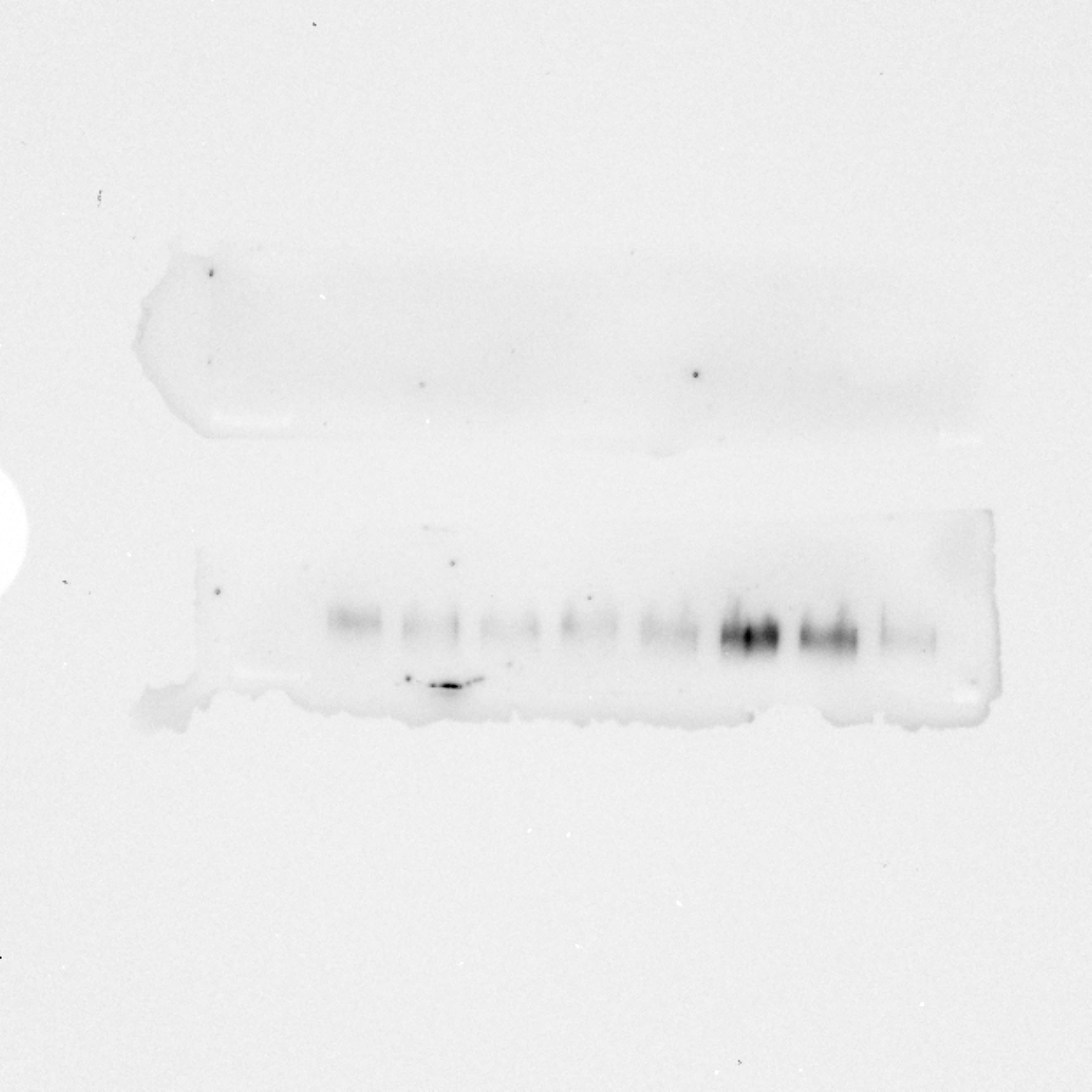

Supplement: Supplementary file 6 — Source data Fig. 2 [file 44318_2024_177_MOESM6_ESM.zip › Figure 2/2K/Western LAMP1 Vesicles/Western LAMP1 Vesicles 20220128_134007_Ch.tif]

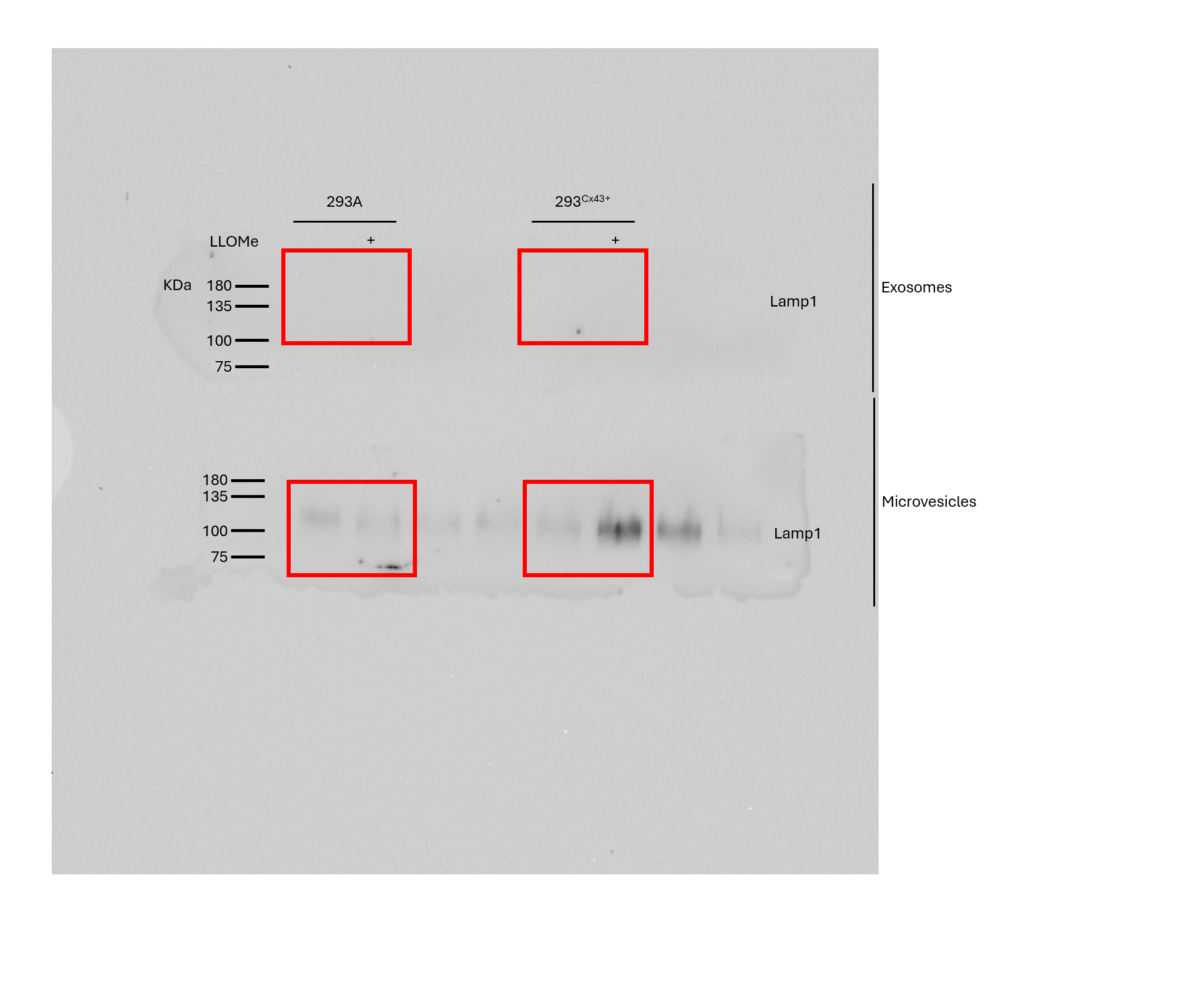

Supplement: Supplementary file 6 — Source data Fig. 2 [file 44318_2024_177_MOESM6_ESM.zip › Figure 2/2K/Western LAMP1 Vesicles/Western LAMP1 Vesicular.tif]

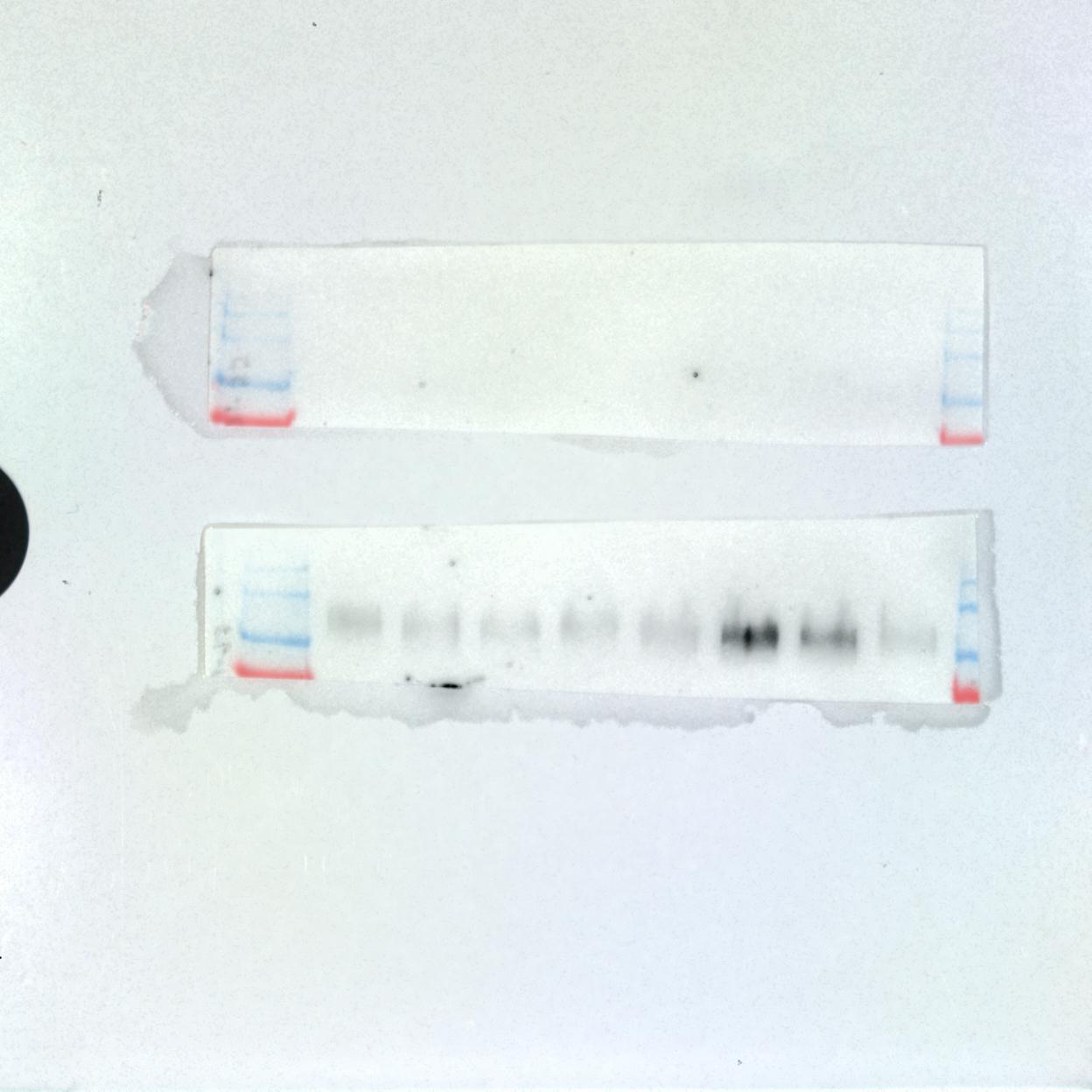

Supplement: Supplementary file 6 — Source data Fig. 2 [file 44318_2024_177_MOESM6_ESM.zip › Figure 2/2K/Western LAMP1 Vesicles/Western LAMP1 Vesicles 20220128_134007_Ch+Marker.jpg]

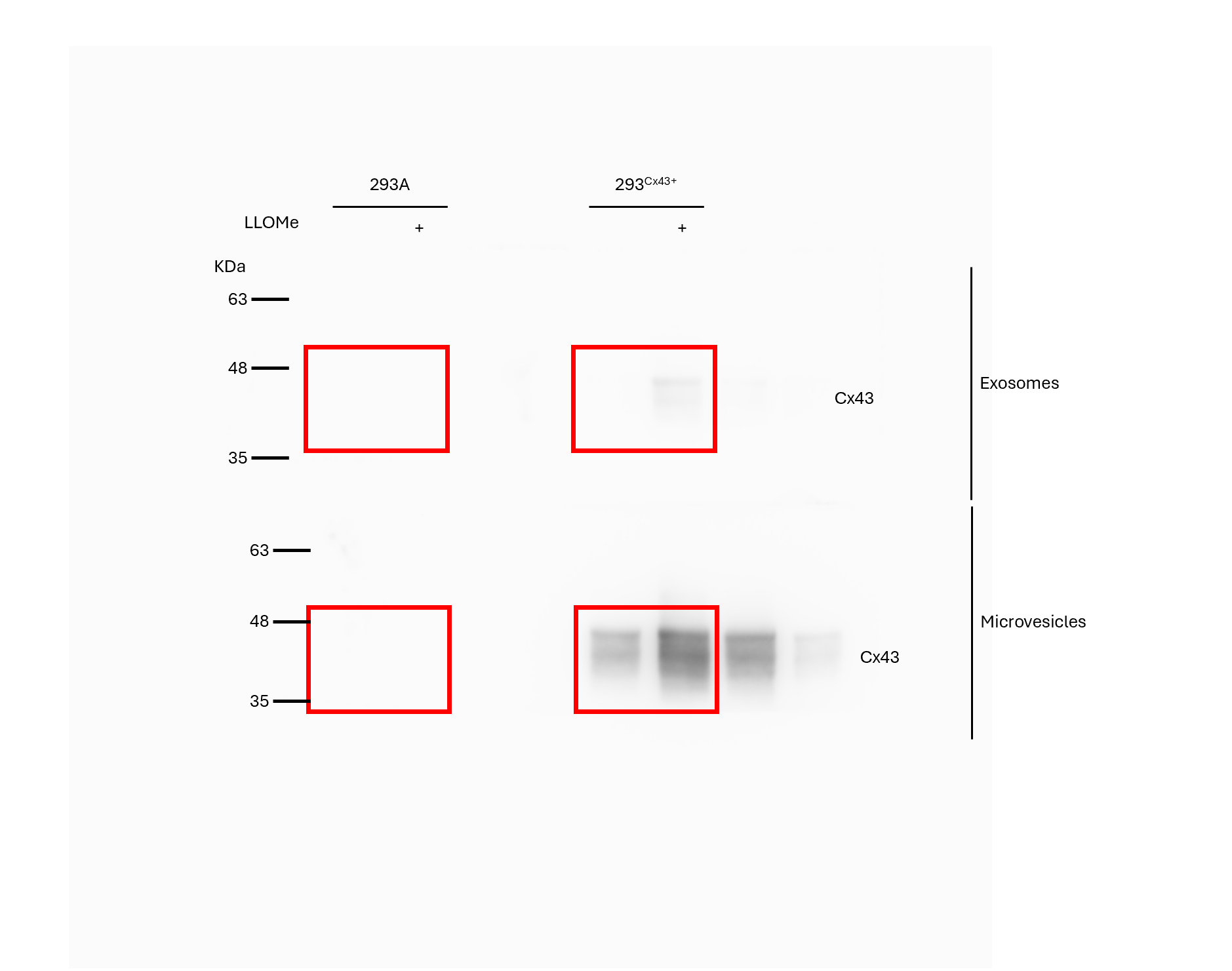

Supplement: Supplementary file 6 — Source data Fig. 2 [file 44318_2024_177_MOESM6_ESM.zip › Figure 2/2K/Western Cx43 Vesicles/Western Cx43 Vesicles.tif]

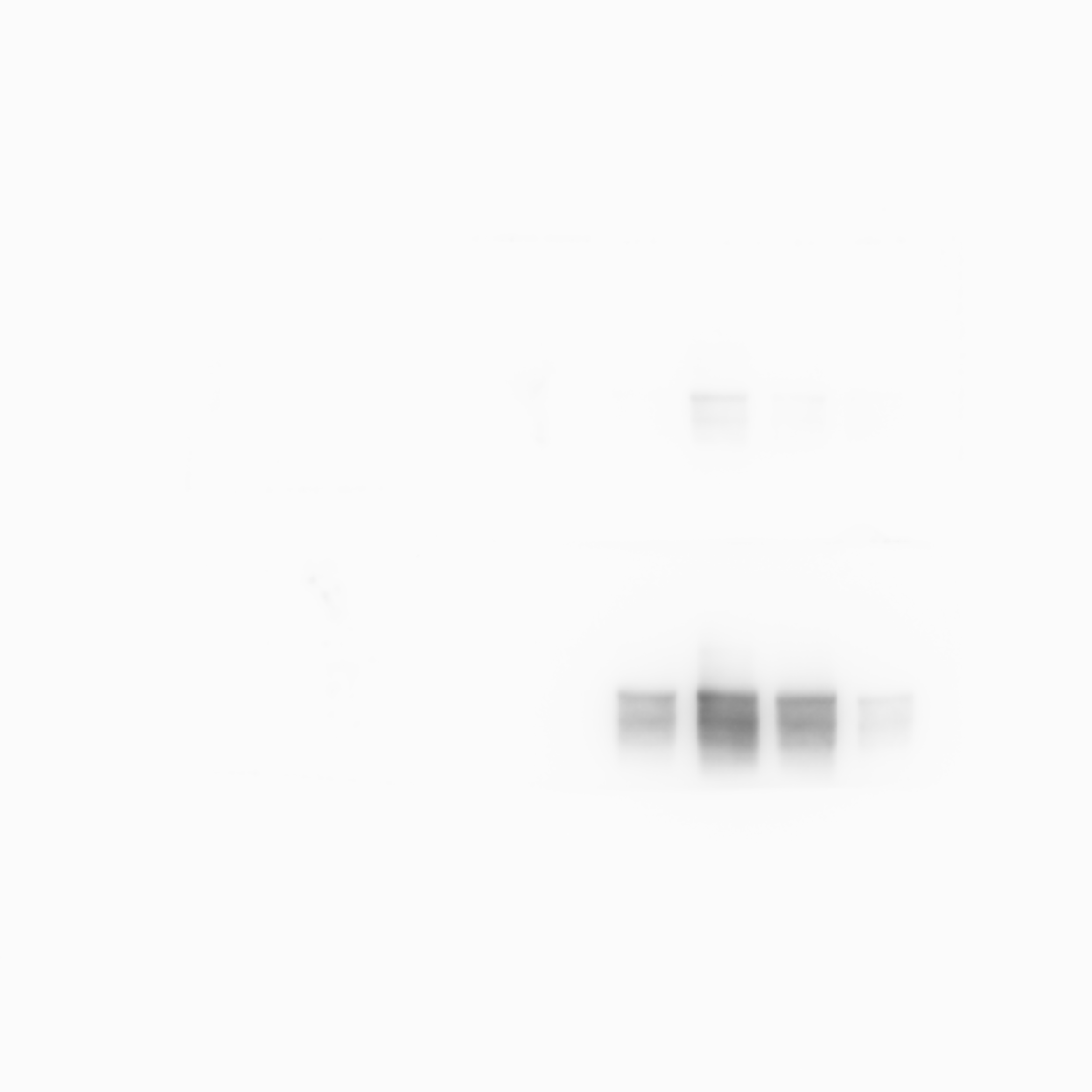

Supplement: Supplementary file 6 — Source data Fig. 2 [file 44318_2024_177_MOESM6_ESM.zip › Figure 2/2K/Western Cx43 Vesicles/Western Cx43 Vesicles 20220128_140639_Ch.tif]

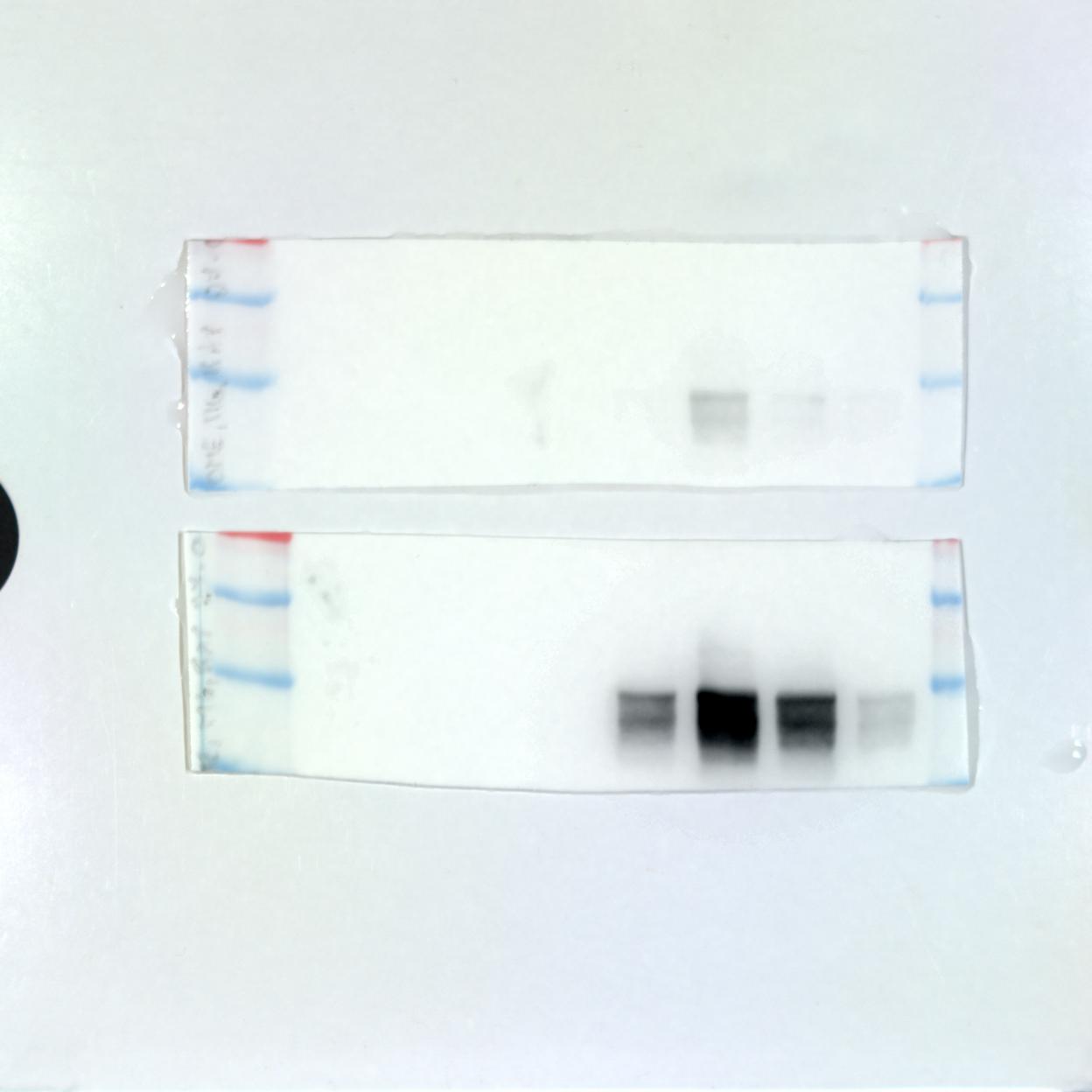

Supplement: Supplementary file 6 — Source data Fig. 2 [file 44318_2024_177_MOESM6_ESM.zip › Figure 2/2K/Western Cx43 Vesicles/Western Cx43 Vesicles 20220128_140639_Ch+Marker.jpg]

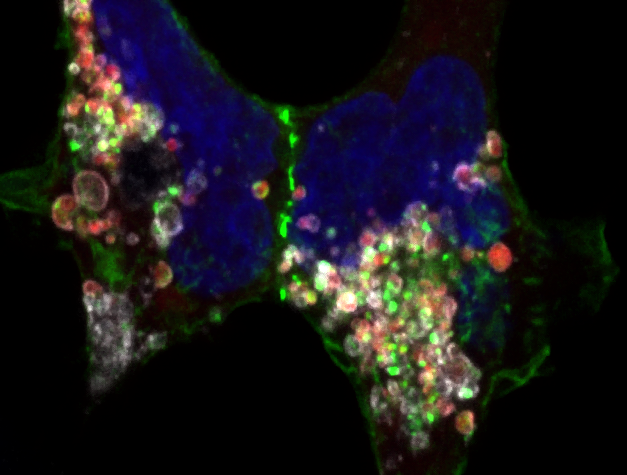

Supplement: Supplementary file 7 — Source data Fig. 3 [file 44318_2024_177_MOESM7_ESM.zip › Figure 3/3A/Microscopy HEK293A+LLOMe Cx43-GFP and Gal3 mCherry LAMP 647.tif.tif]

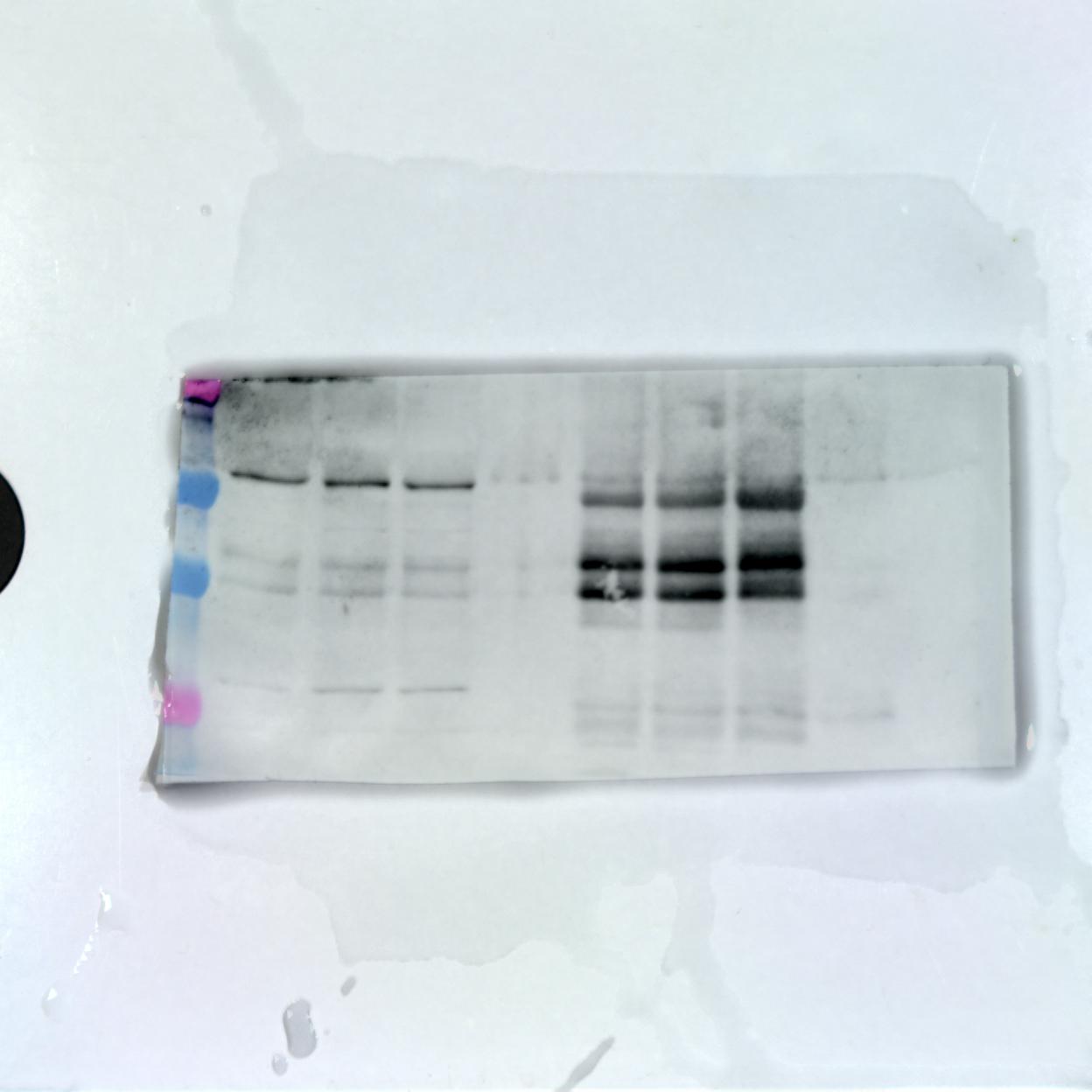

Supplement: Supplementary file 7 — Source data Fig. 3 [file 44318_2024_177_MOESM7_ESM.zip › Figure 3/3F/Western Cx43/Western Cx43 20210409_095324_Ch+Marker.jpg]

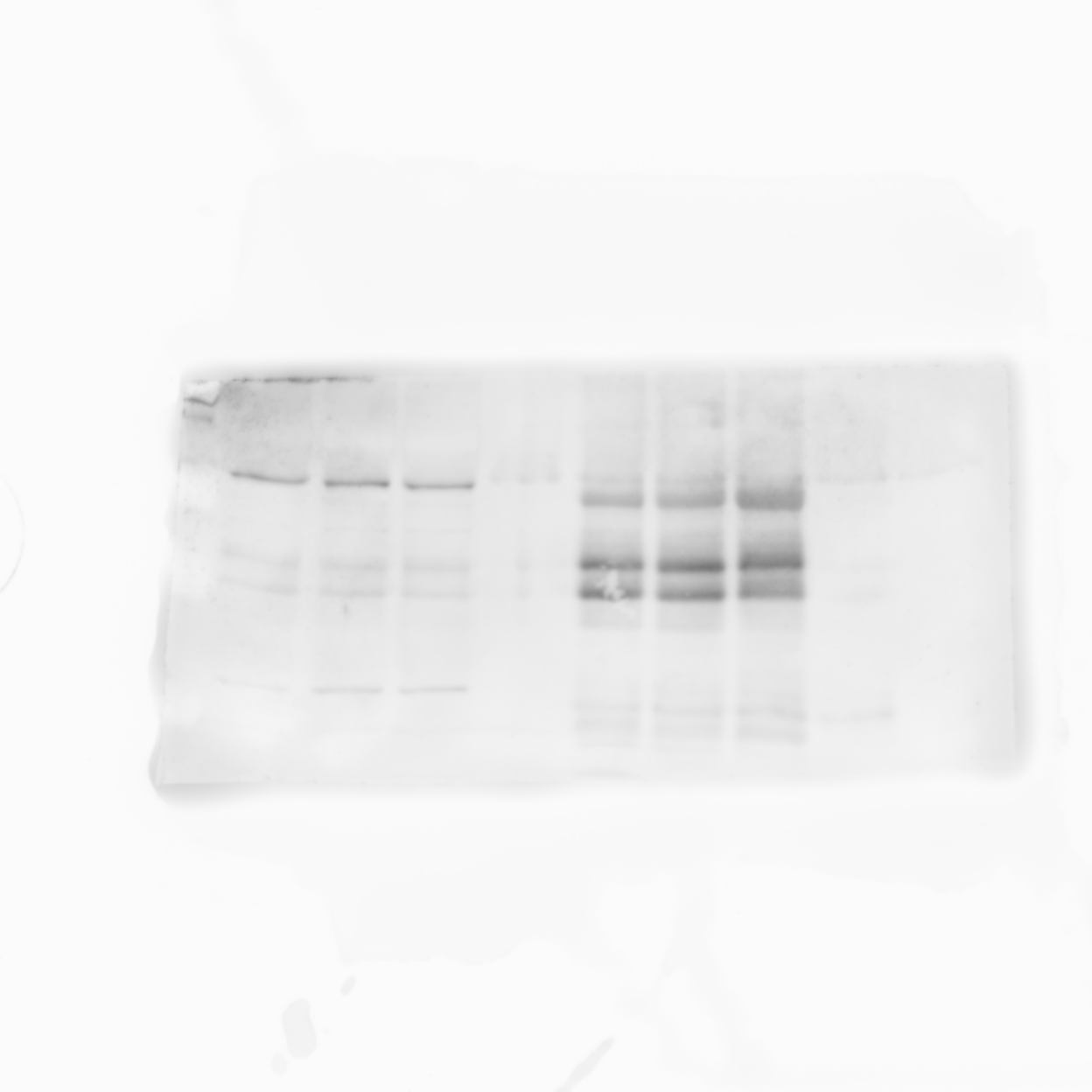

Supplement: Supplementary file 7 — Source data Fig. 3 [file 44318_2024_177_MOESM7_ESM.zip › Figure 3/3F/Western Cx43/Western Cx43 20210409_095324_Ch.tif]

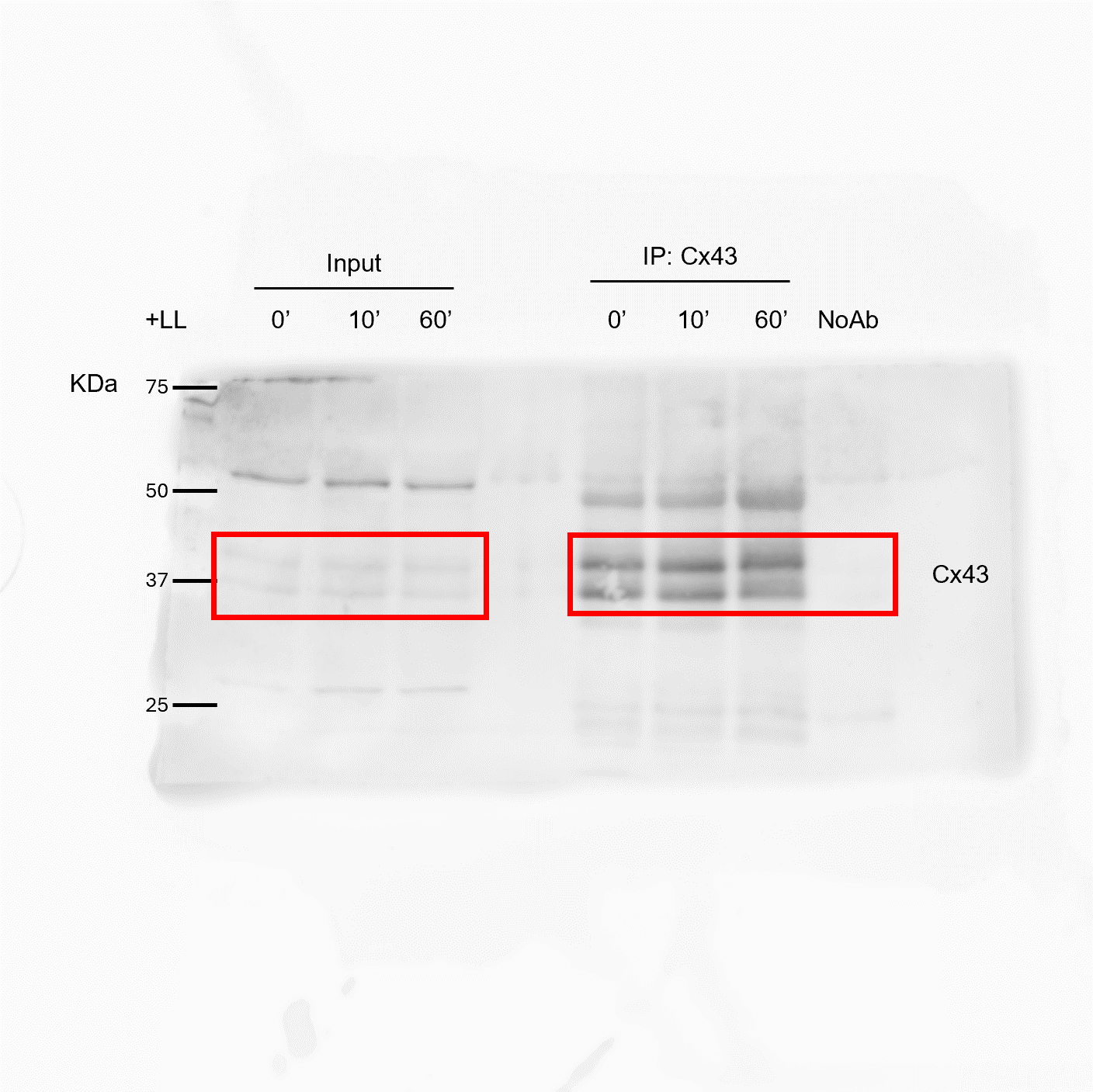

Supplement: Supplementary file 7 — Source data Fig. 3 [file 44318_2024_177_MOESM7_ESM.zip › Figure 3/3F/Western Cx43/Western Cx43.png]

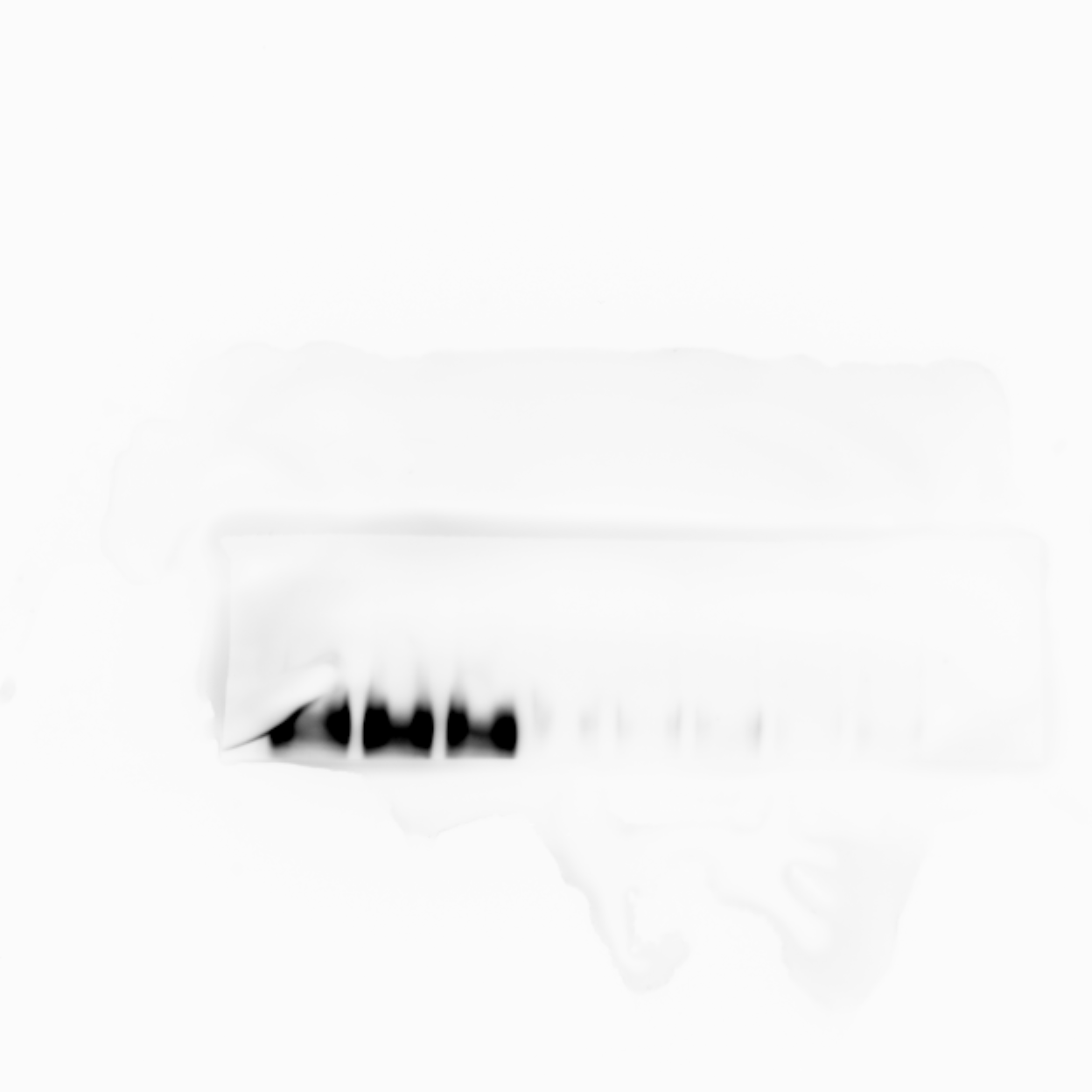

Supplement: Supplementary file 7 — Source data Fig. 3 [file 44318_2024_177_MOESM7_ESM.zip › Figure 3/3F/Western LAMP1/Western LAMP1 20210408_115045_Ch.tif]

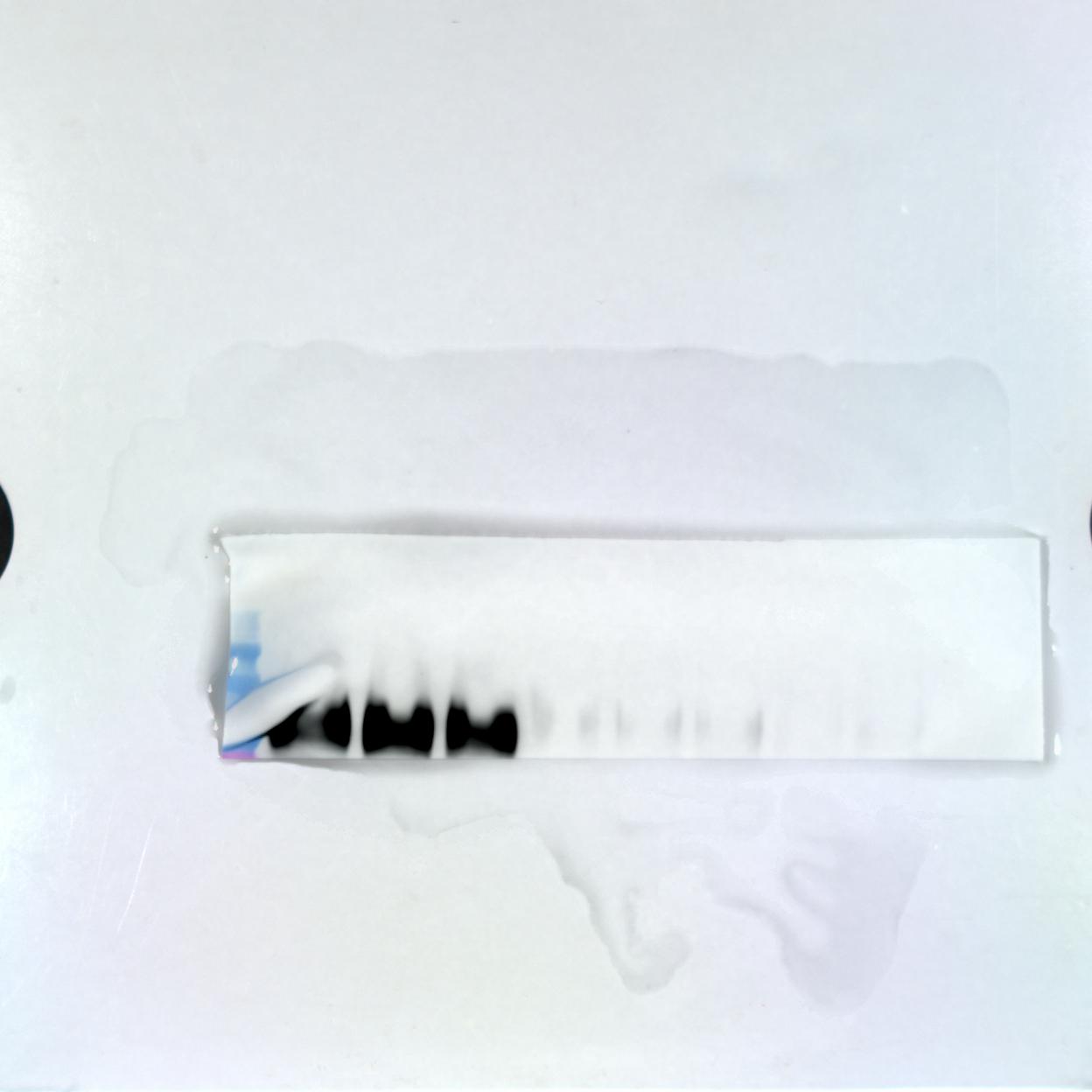

Supplement: Supplementary file 7 — Source data Fig. 3 [file 44318_2024_177_MOESM7_ESM.zip › Figure 3/3F/Western LAMP1/Western LAMP1 20210408_115045_Ch+Marker.jpg]

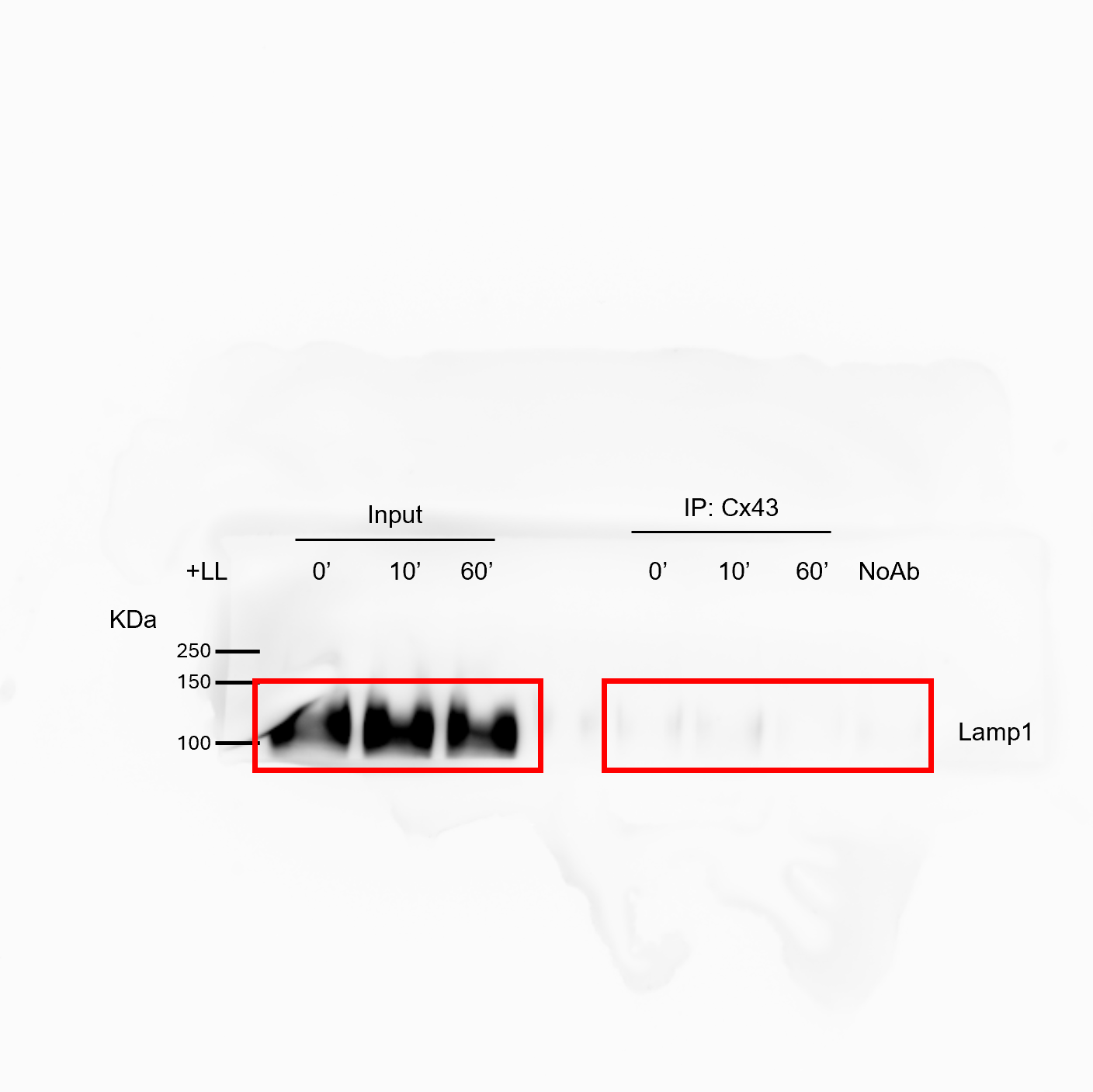

Supplement: Supplementary file 7 — Source data Fig. 3 [file 44318_2024_177_MOESM7_ESM.zip › Figure 3/3F/Western LAMP1/Western LAMP1.tif]

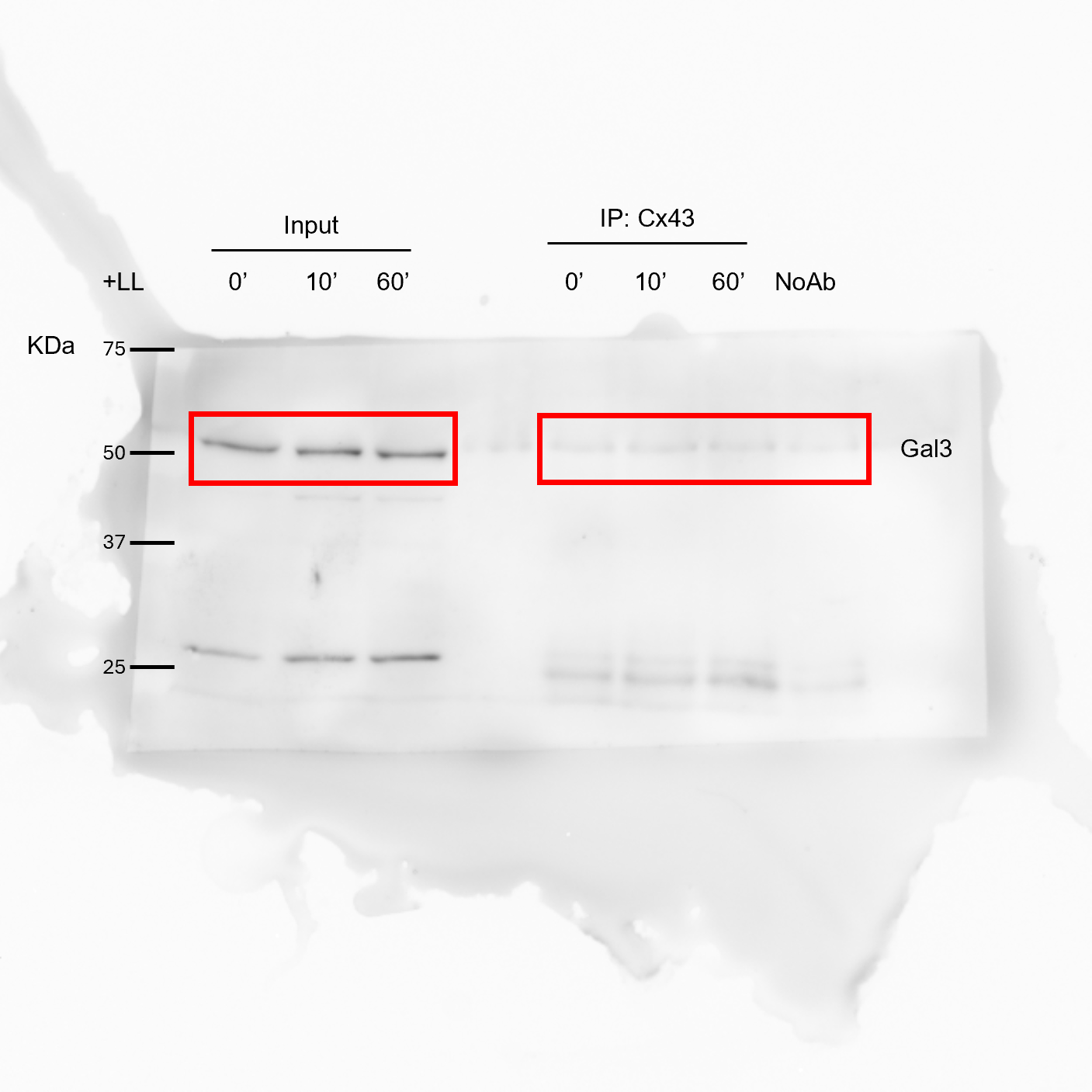

Supplement: Supplementary file 7 — Source data Fig. 3 [file 44318_2024_177_MOESM7_ESM.zip › Figure 3/3F/Western Gal3/Western Gal3.tif]

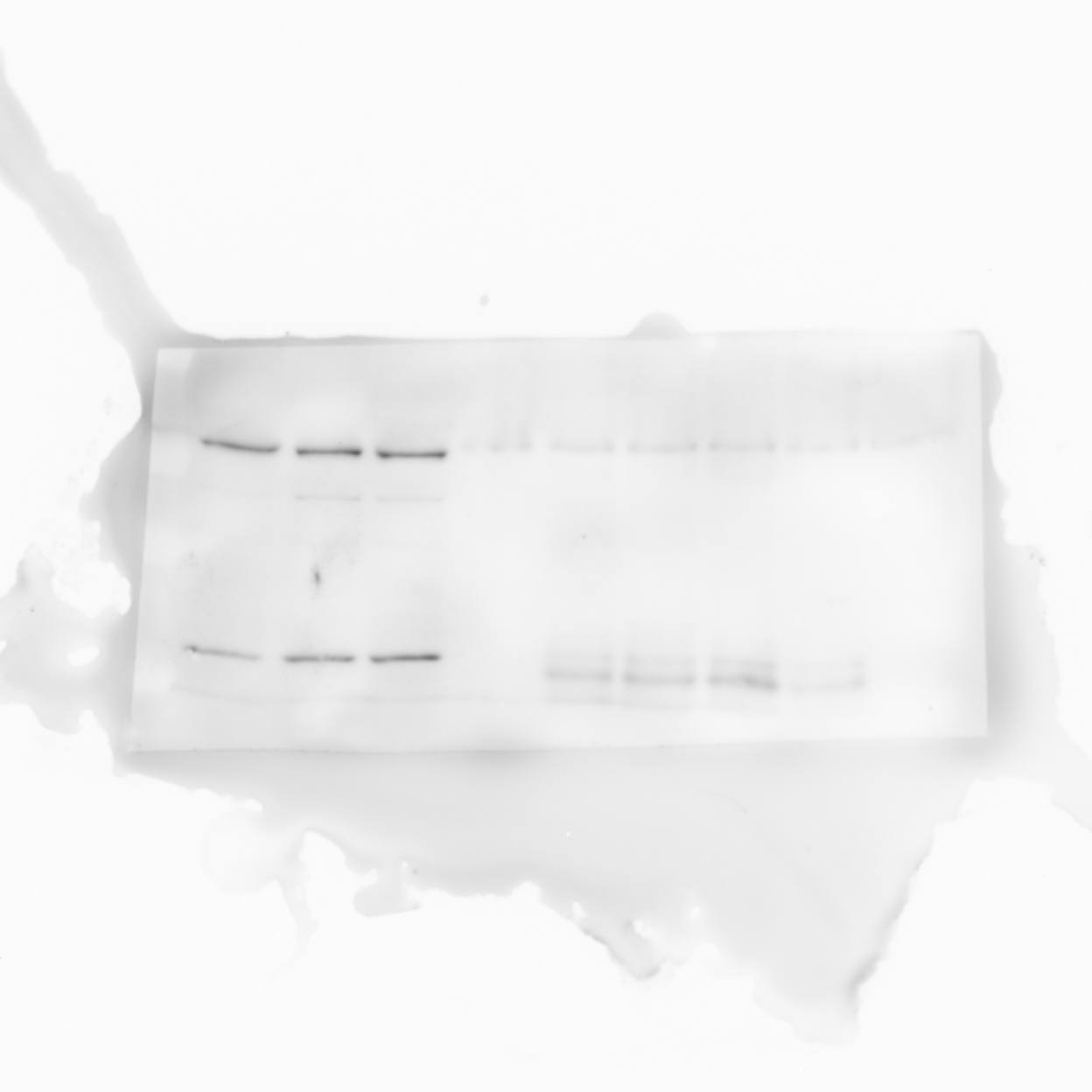

Supplement: Supplementary file 7 — Source data Fig. 3 [file 44318_2024_177_MOESM7_ESM.zip › Figure 3/3F/Western Gal3/Western Gal3 20210408_115450-12_Ch.tif]

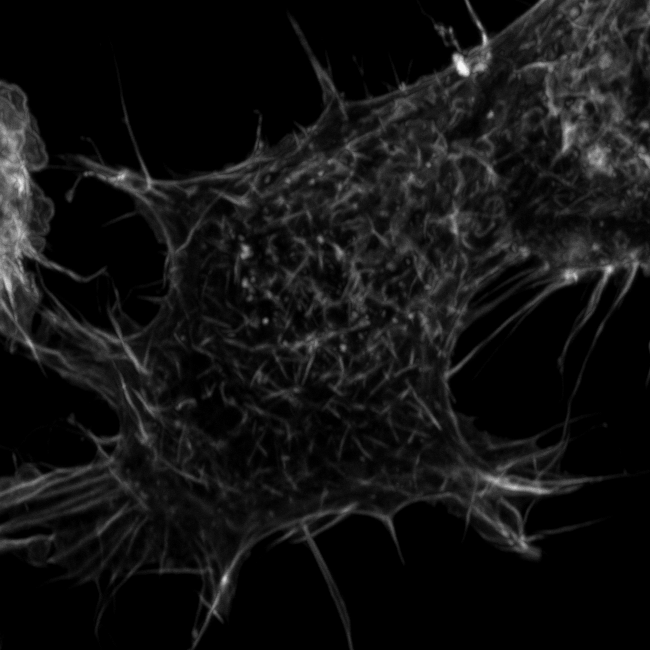

Supplement: Supplementary file 9 — Source data Fig. 5 [file 44318_2024_177_MOESM9_ESM.zip › Figure 5/5C/Fluorescence Microscopy HEK293A LLOMe phalloindin.tif]

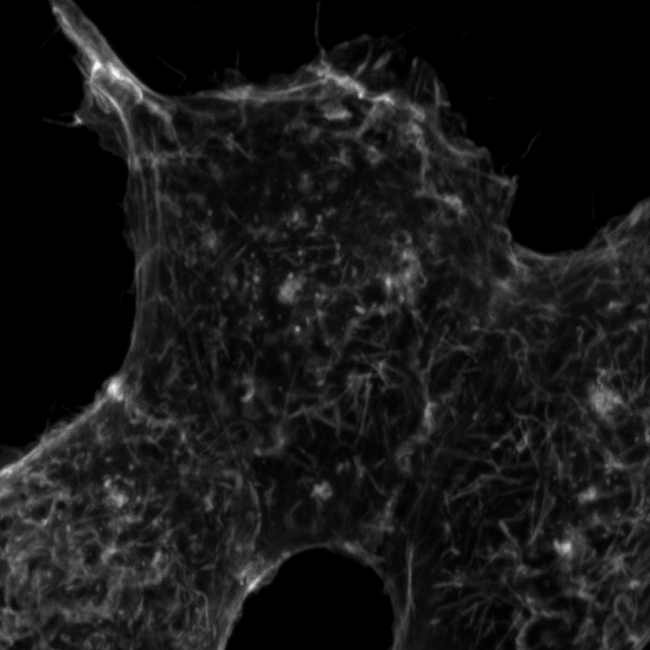

Supplement: Supplementary file 9 — Source data Fig. 5 [file 44318_2024_177_MOESM9_ESM.zip › Figure 5/5C/Fluorescence Microscopy HEK293Cx43+ LLOMe phalloindin.tif]

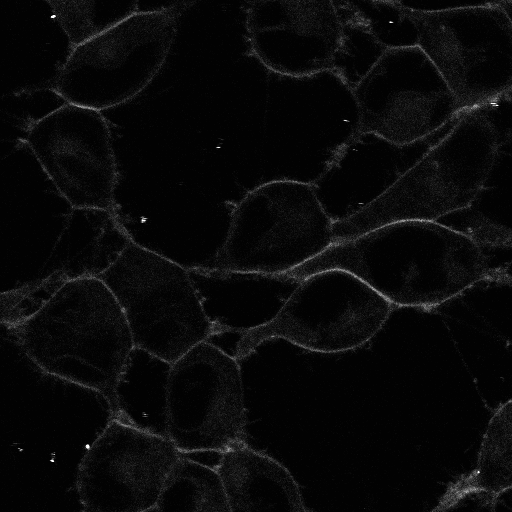

Supplement: Supplementary file 9 — Source data Fig. 5 [file 44318_2024_177_MOESM9_ESM.zip › Figure 5/5F/Two-photon excitation microscopy HEK293A Ct_ch00.tif]

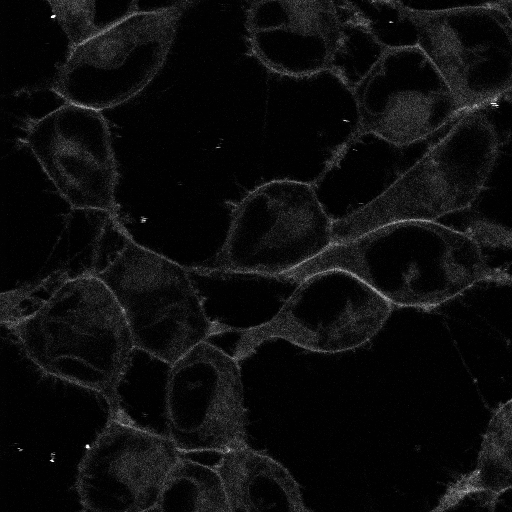

Supplement: Supplementary file 9 — Source data Fig. 5 [file 44318_2024_177_MOESM9_ESM.zip › Figure 5/5F/Two-photon excitation microscopy HEK293A Ct_ch01e.tif]

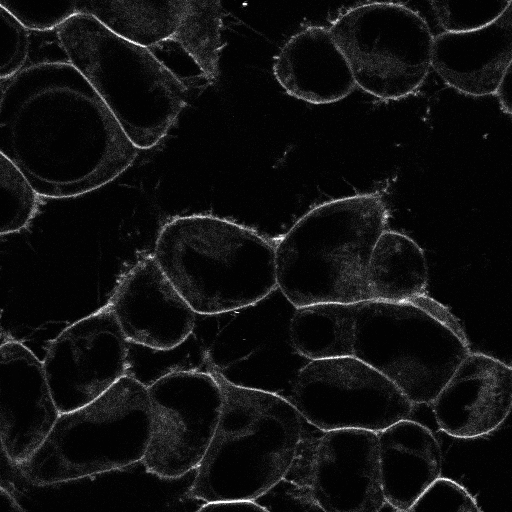

Supplement: Supplementary file 9 — Source data Fig. 5 [file 44318_2024_177_MOESM9_ESM.zip › Figure 5/5F/Two-photon excitation microscopy HEK293A LLOMe_ch00.tif]

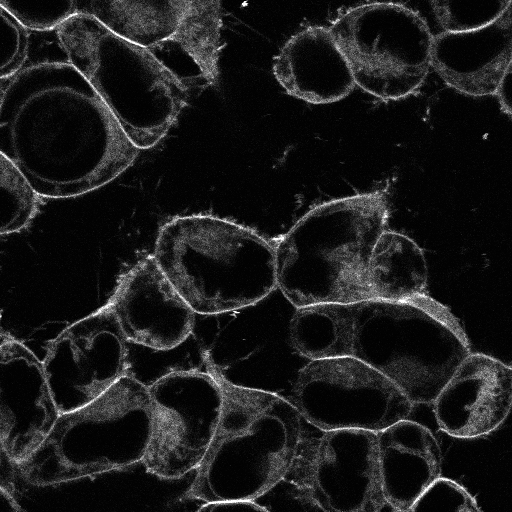

Supplement: Supplementary file 9 — Source data Fig. 5 [file 44318_2024_177_MOESM9_ESM.zip › Figure 5/5F/Two-photon excitation microscopy HEK293A LLOMe_ch01.tif]

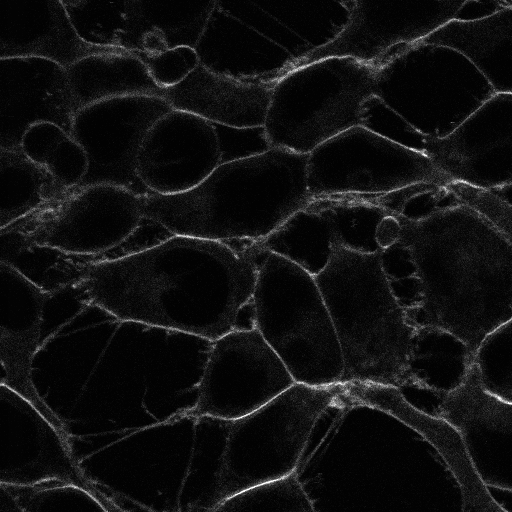

Supplement: Supplementary file 9 — Source data Fig. 5 [file 44318_2024_177_MOESM9_ESM.zip › Figure 5/5F/Two-photon excitation microscopy HEK293Cx43+ Ct_ch00.tif]

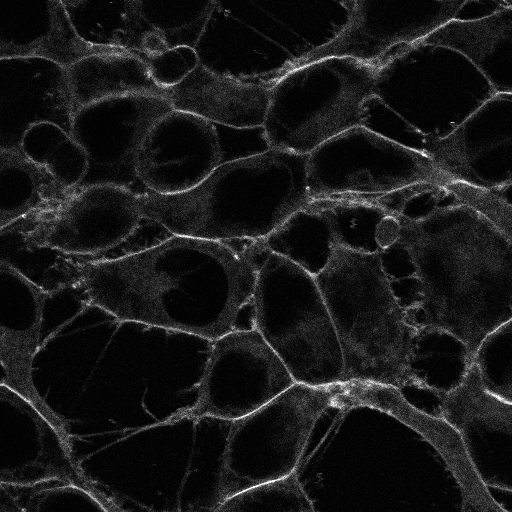

Supplement: Supplementary file 9 — Source data Fig. 5 [file 44318_2024_177_MOESM9_ESM.zip › Figure 5/5F/Two-photon excitation microscopy HEK293Cx43+ Ct_ch01.tif]

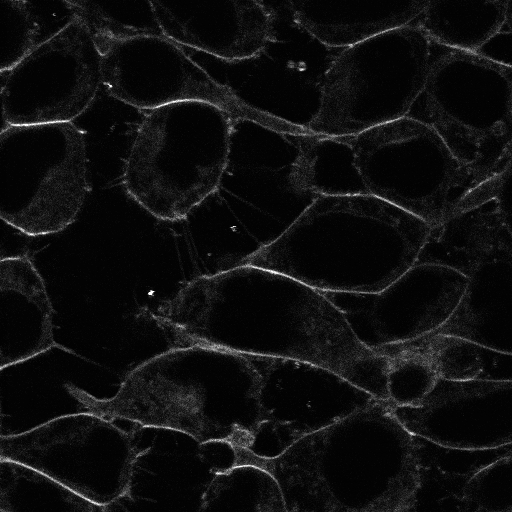

Supplement: Supplementary file 9 — Source data Fig. 5 [file 44318_2024_177_MOESM9_ESM.zip › Figure 5/5F/Two-photon excitation microscopy HEK293Cx43+ LLOMe_ch00.tif]

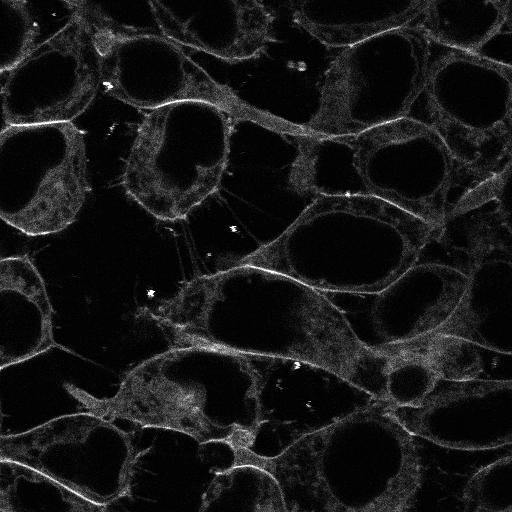

Supplement: Supplementary file 9 — Source data Fig. 5 [file 44318_2024_177_MOESM9_ESM.zip › Figure 5/5F/Two-photon excitation microscopy HEK293Cx43+ LLOMe_ch01.tif]

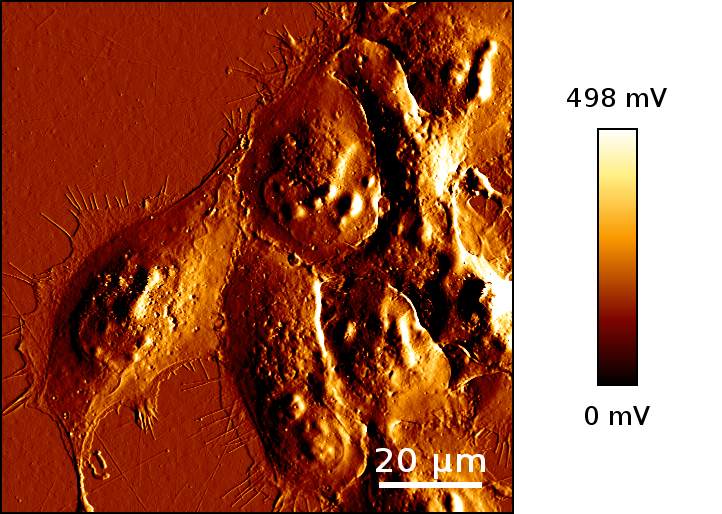

Supplement: Supplementary file 9 — Source data Fig. 5 [file 44318_2024_177_MOESM9_ESM.zip › Figure 5/5H/Atomic force microscopy HEK293A Ct error_trace.tiff]

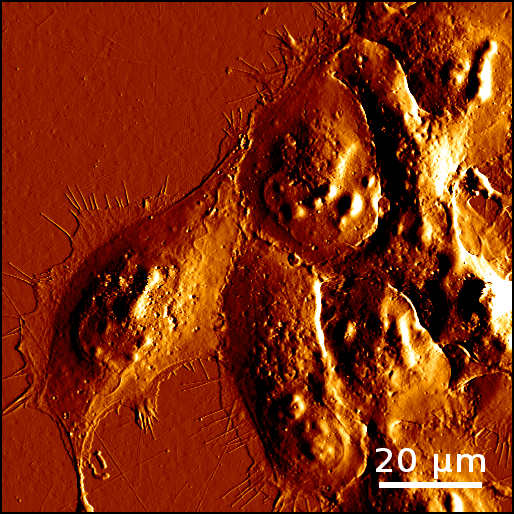

Supplement: Supplementary file 9 — Source data Fig. 5 [file 44318_2024_177_MOESM9_ESM.zip › Figure 5/5H/Atomic force microscopy HEK293A Ct.tiff]

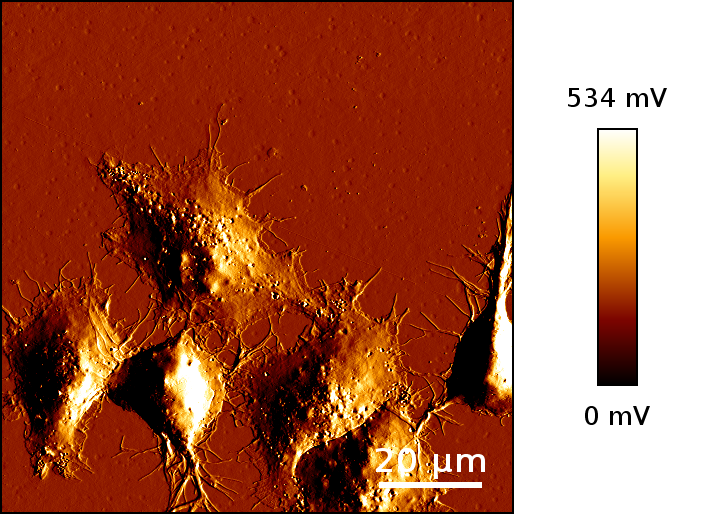

Supplement: Supplementary file 9 — Source data Fig. 5 [file 44318_2024_177_MOESM9_ESM.zip › Figure 5/5H/Atomic force microscopy HEK293A LLOMe error_trace.tiff]

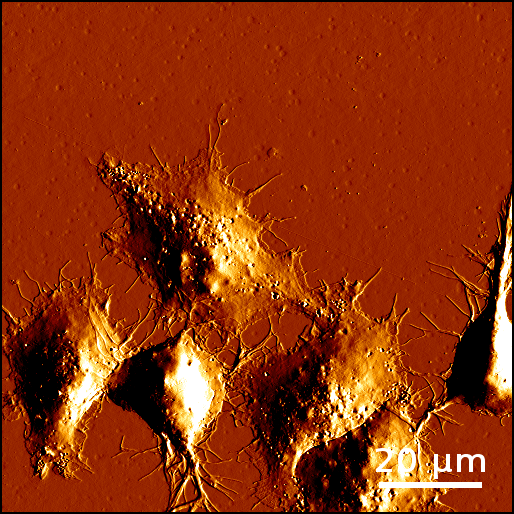

Supplement: Supplementary file 9 — Source data Fig. 5 [file 44318_2024_177_MOESM9_ESM.zip › Figure 5/5H/Atomic force microscopy HEK293A LLOMe.tiff]

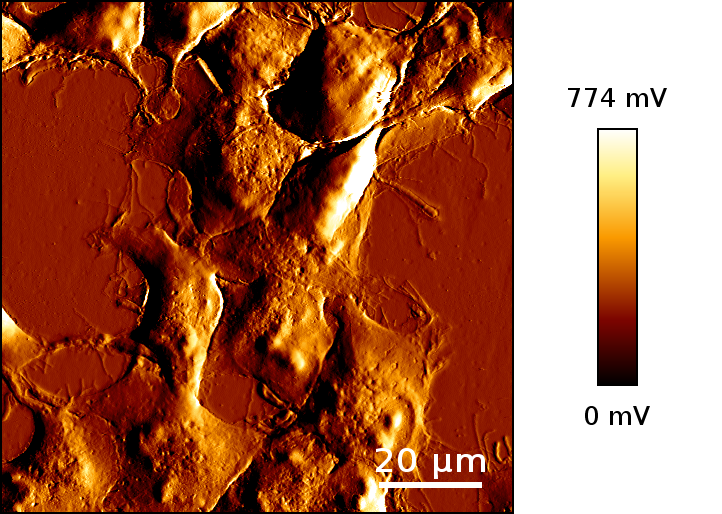

Supplement: Supplementary file 9 — Source data Fig. 5 [file 44318_2024_177_MOESM9_ESM.zip › Figure 5/5H/Atomic force microscopy HEK293Cx43+ Ct error_trace.tiff]

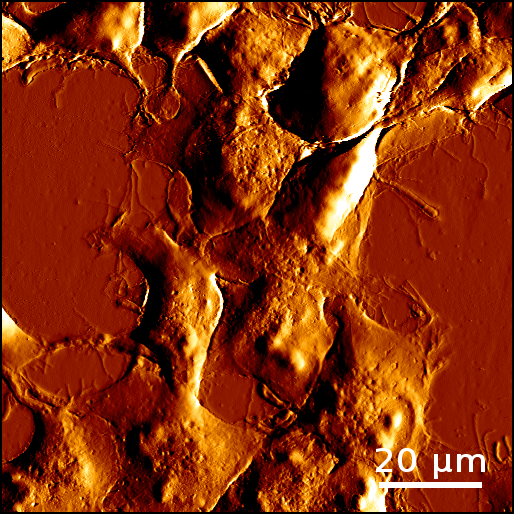

Supplement: Supplementary file 9 — Source data Fig. 5 [file 44318_2024_177_MOESM9_ESM.zip › Figure 5/5H/Atomic force microscopy HEK293Cx43+ Ct.tiff]

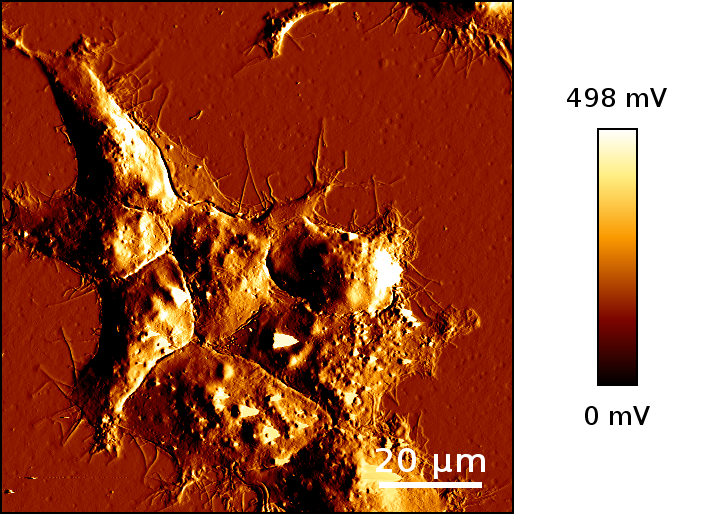

Supplement: Supplementary file 9 — Source data Fig. 5 [file 44318_2024_177_MOESM9_ESM.zip › Figure 5/5H/Atomic force microscopy HEK293Cx43+ LLOMe error_trace.tiff]

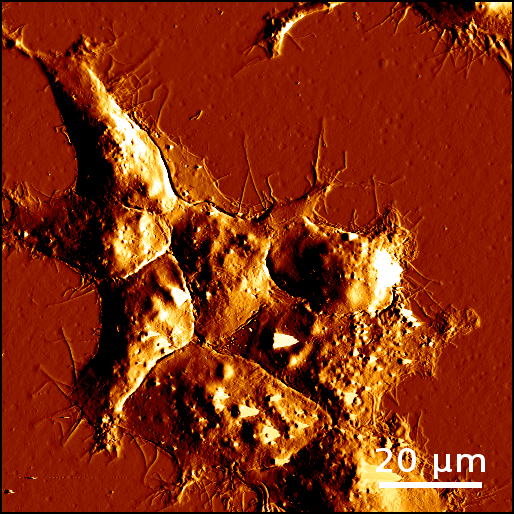

Supplement: Supplementary file 9 — Source data Fig. 5 [file 44318_2024_177_MOESM9_ESM.zip › Figure 5/5H/Atomic force microscopy HEK293Cx43+ LLOMe.tiff]

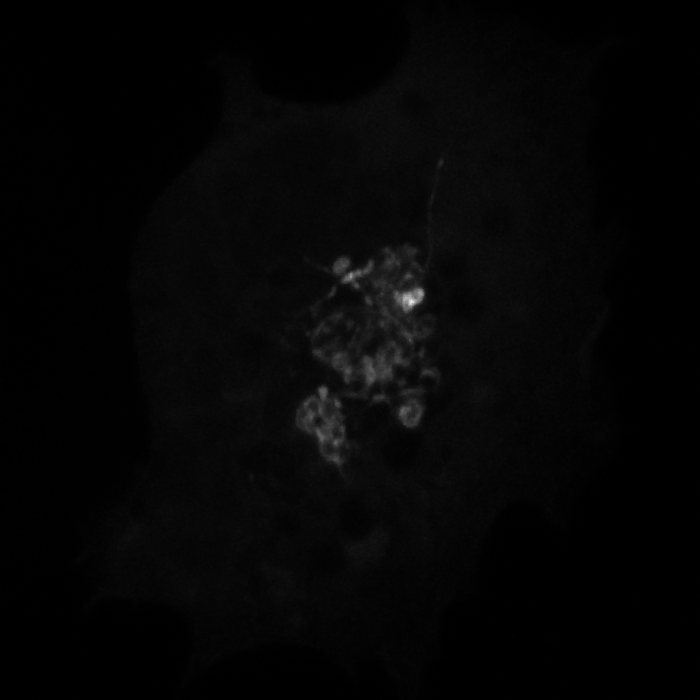

Supplement: Supplementary file 10 — Source data Fig. 6 [file 44318_2024_177_MOESM10_ESM.zip › Figure 6/6G/Fluorescence Microscopy HEK293Cx43+ Salmonella mCherry-Gal3 GFP-Arp2 Cx43 647 ARP2 GFP A.tif]

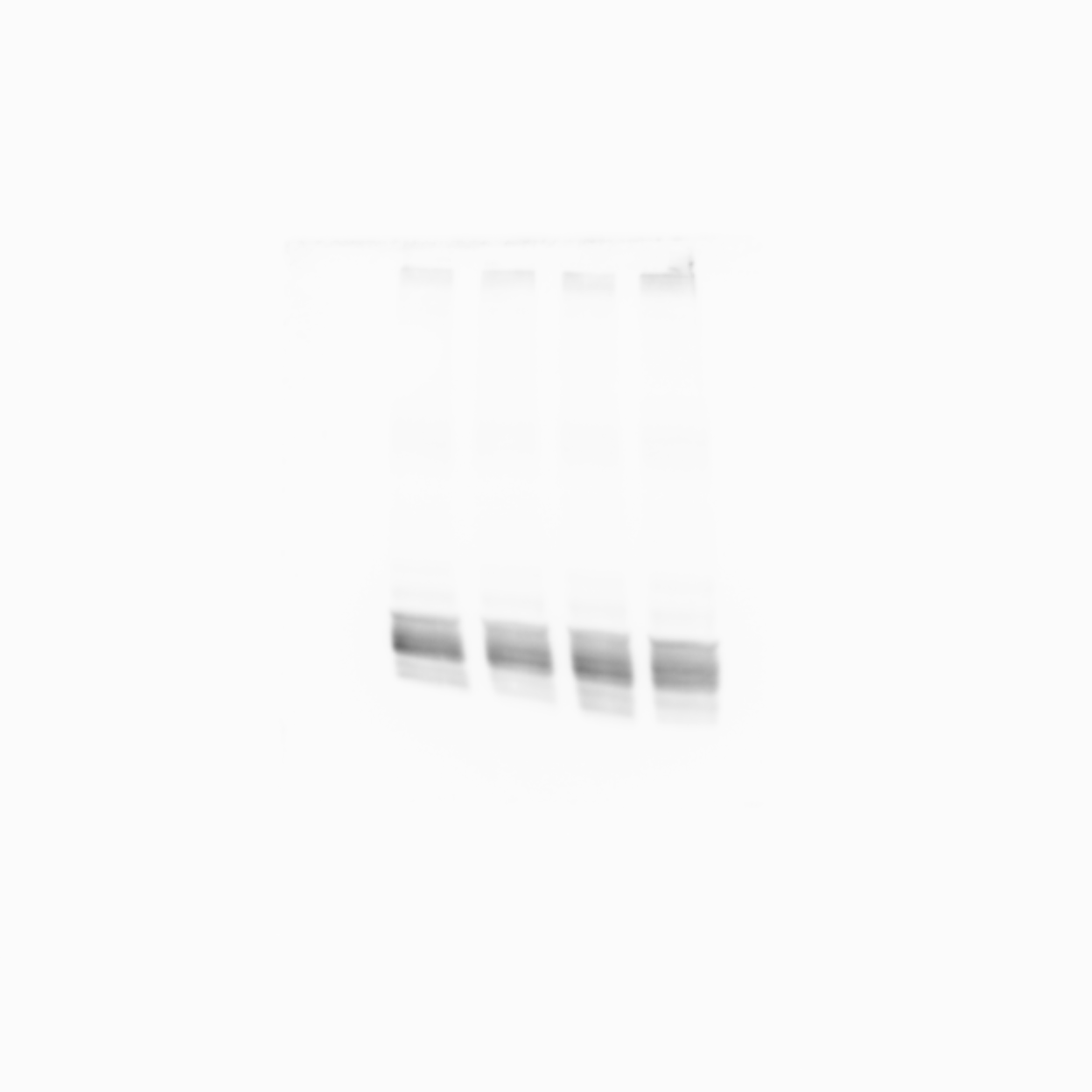

Supplement: Supplementary file 10 — Source data Fig. 6 [file 44318_2024_177_MOESM10_ESM.zip › Figure 6/6H/Input Cx43/Western Cx43 Input 20211028_161618_Ch.tif]

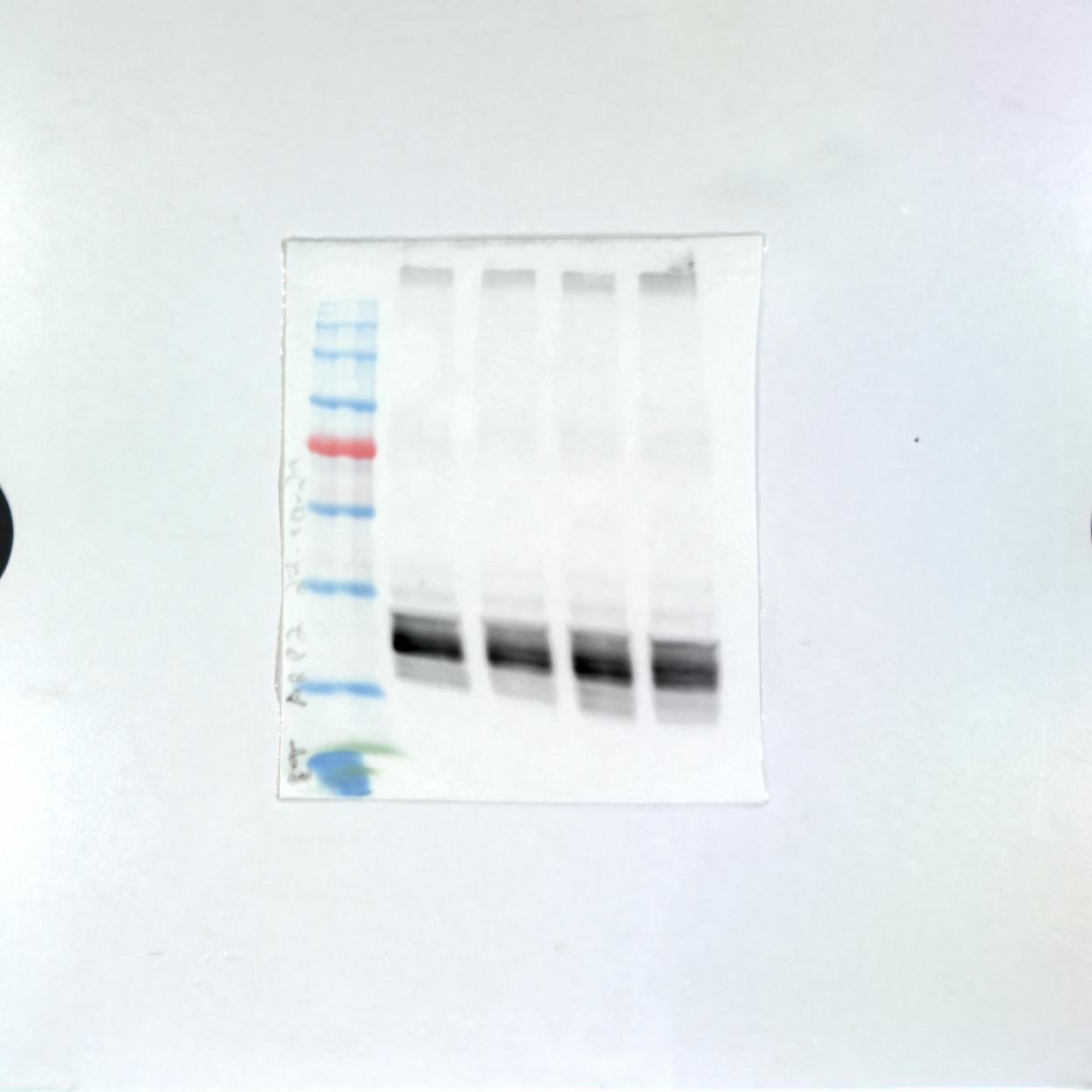

Supplement: Supplementary file 10 — Source data Fig. 6 [file 44318_2024_177_MOESM10_ESM.zip › Figure 6/6H/Input Cx43/Western Cx43 Input 20211028_161618_Ch+Marker.jpg]

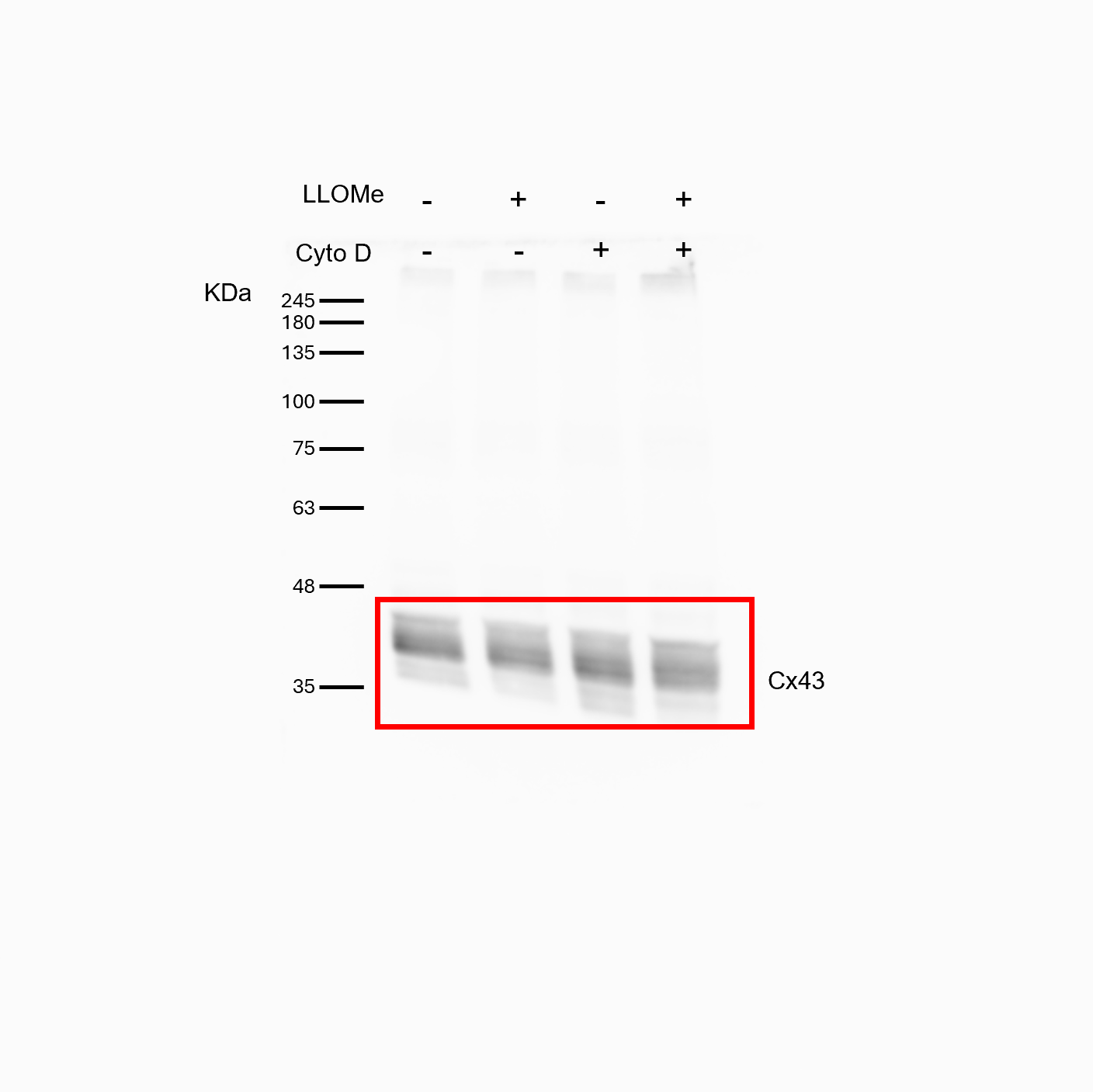

Supplement: Supplementary file 10 — Source data Fig. 6 [file 44318_2024_177_MOESM10_ESM.zip › Figure 6/6H/Input Cx43/Western Cx43 Input.tif]

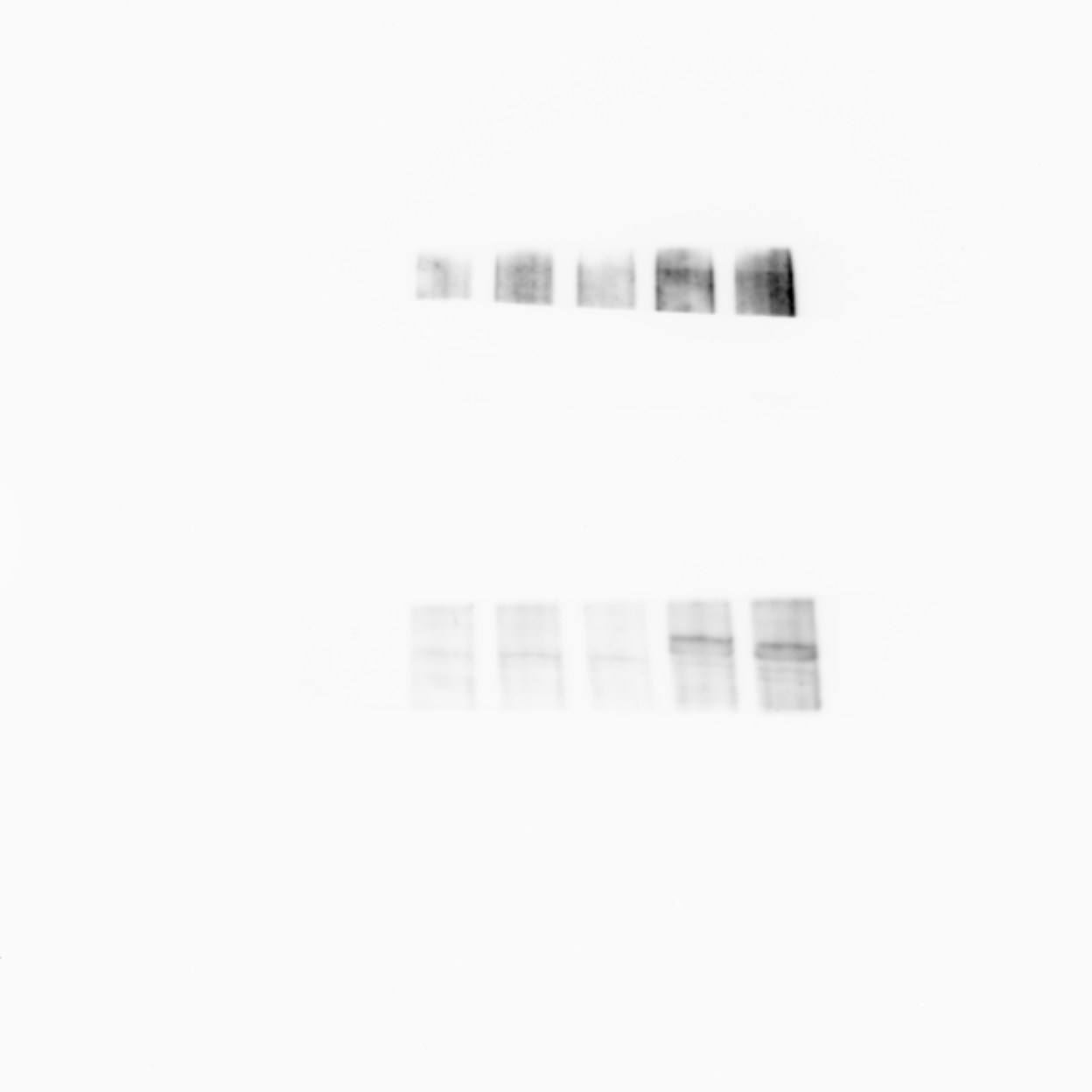

Supplement: Supplementary file 10 — Source data Fig. 6 [file 44318_2024_177_MOESM10_ESM.zip › Figure 6/6H/IP Arp2/Western Arp2-2 IP 20211029_135444_Ch.tif]

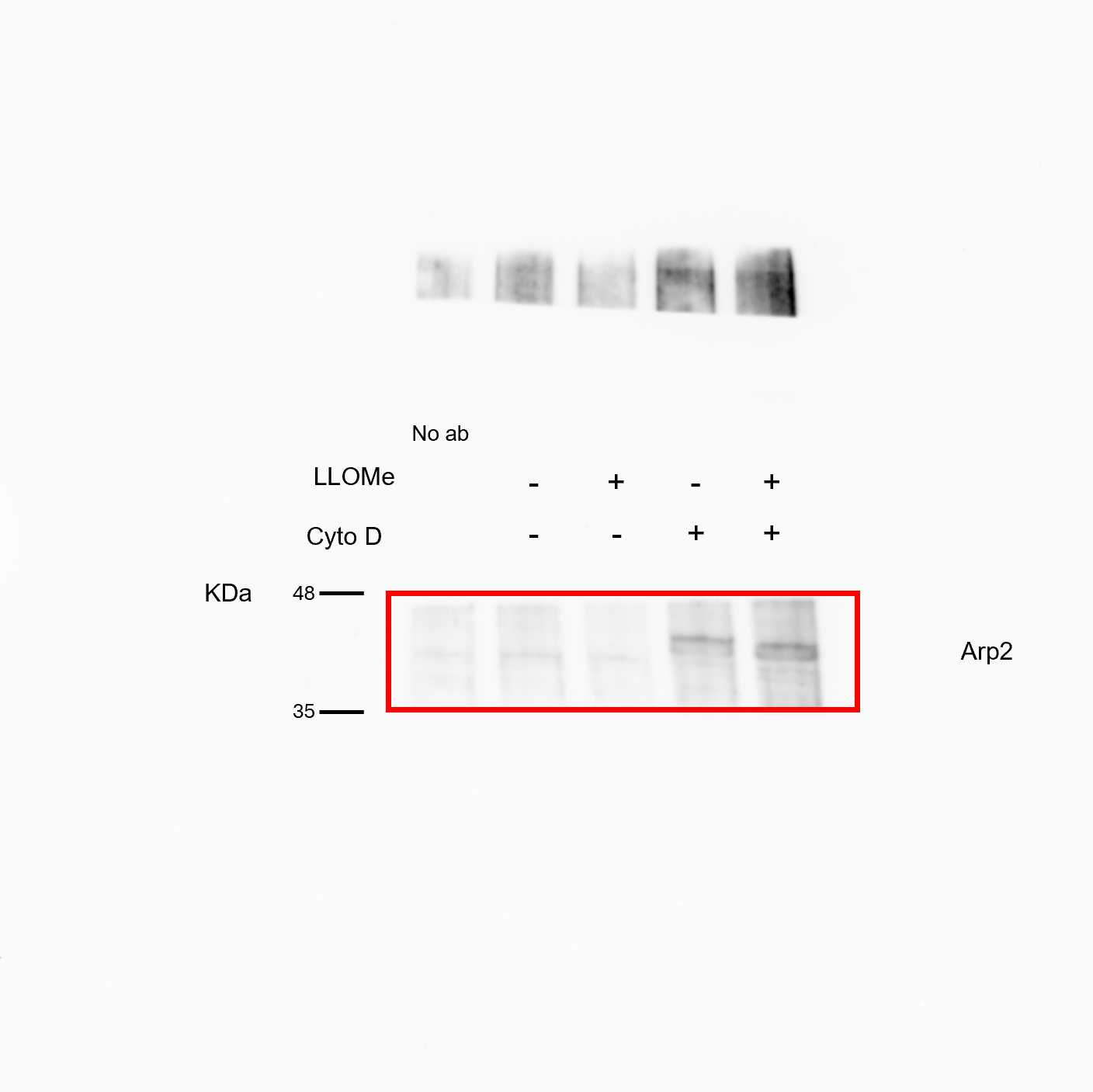

Supplement: Supplementary file 10 — Source data Fig. 6 [file 44318_2024_177_MOESM10_ESM.zip › Figure 6/6H/IP Arp2/Western Arp2 IP.tif]

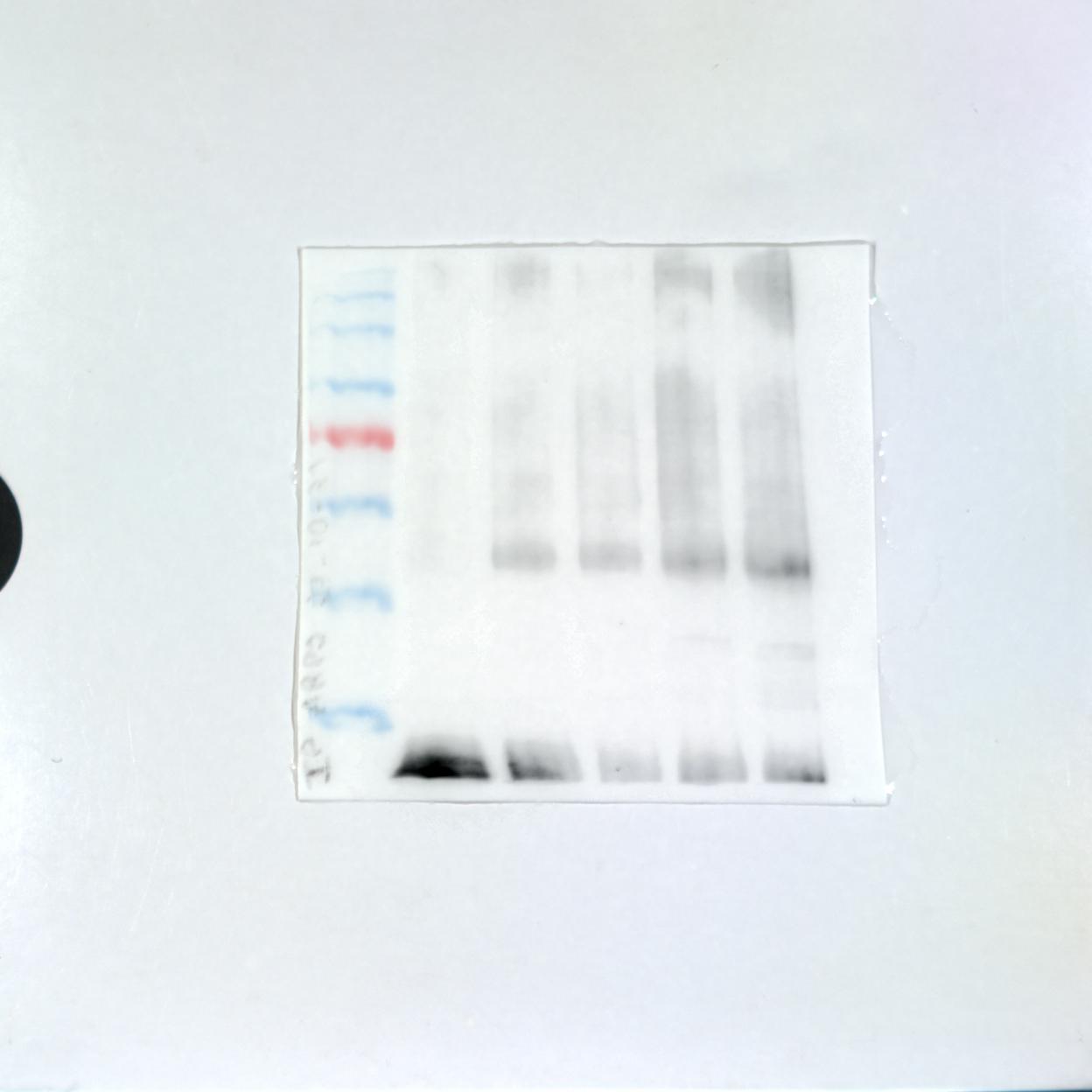

Supplement: Supplementary file 10 — Source data Fig. 6 [file 44318_2024_177_MOESM10_ESM.zip › Figure 6/6H/IP Arp2/Western Arp2 IP 20211029_134921_Ch+Marker.jpg]

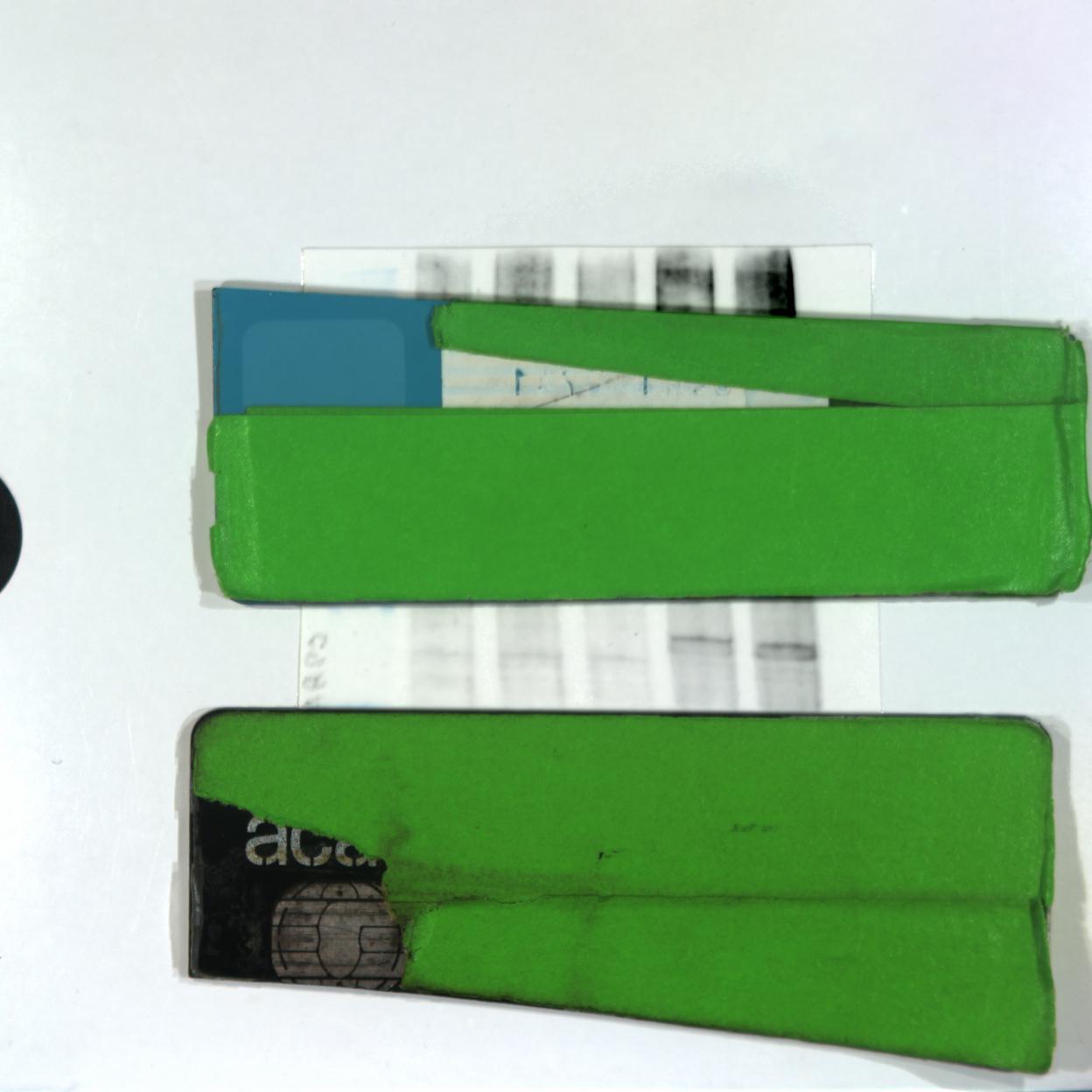

Supplement: Supplementary file 10 — Source data Fig. 6 [file 44318_2024_177_MOESM10_ESM.zip › Figure 6/6H/IP Arp2/Western Arp2-2 IP 20211029_135444_Ch+Marker.jpg]

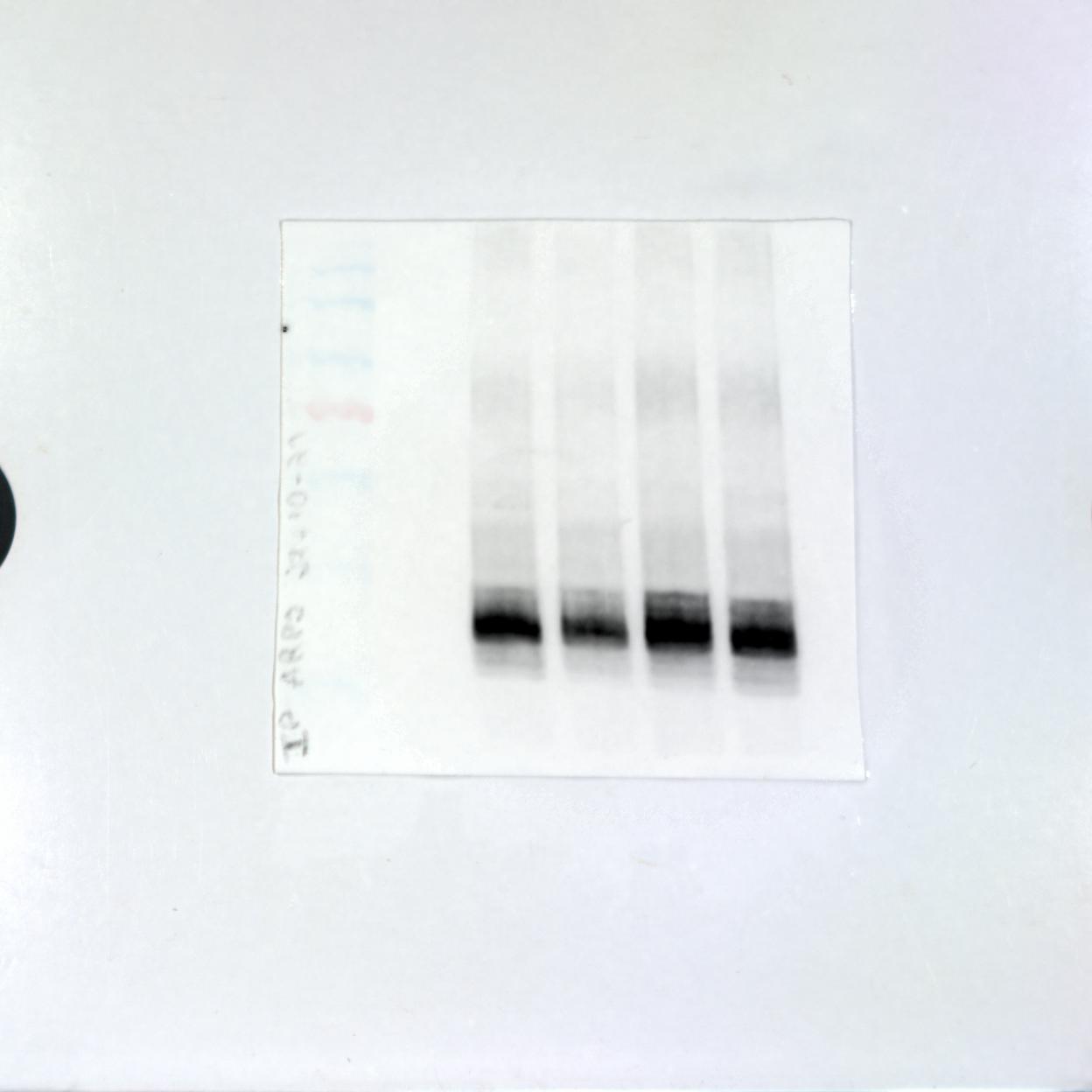

Supplement: Supplementary file 10 — Source data Fig. 6 [file 44318_2024_177_MOESM10_ESM.zip › Figure 6/6H/IP Cx43/Western Cx43 IP 20211103_152318_Ch+Marker.jpg]

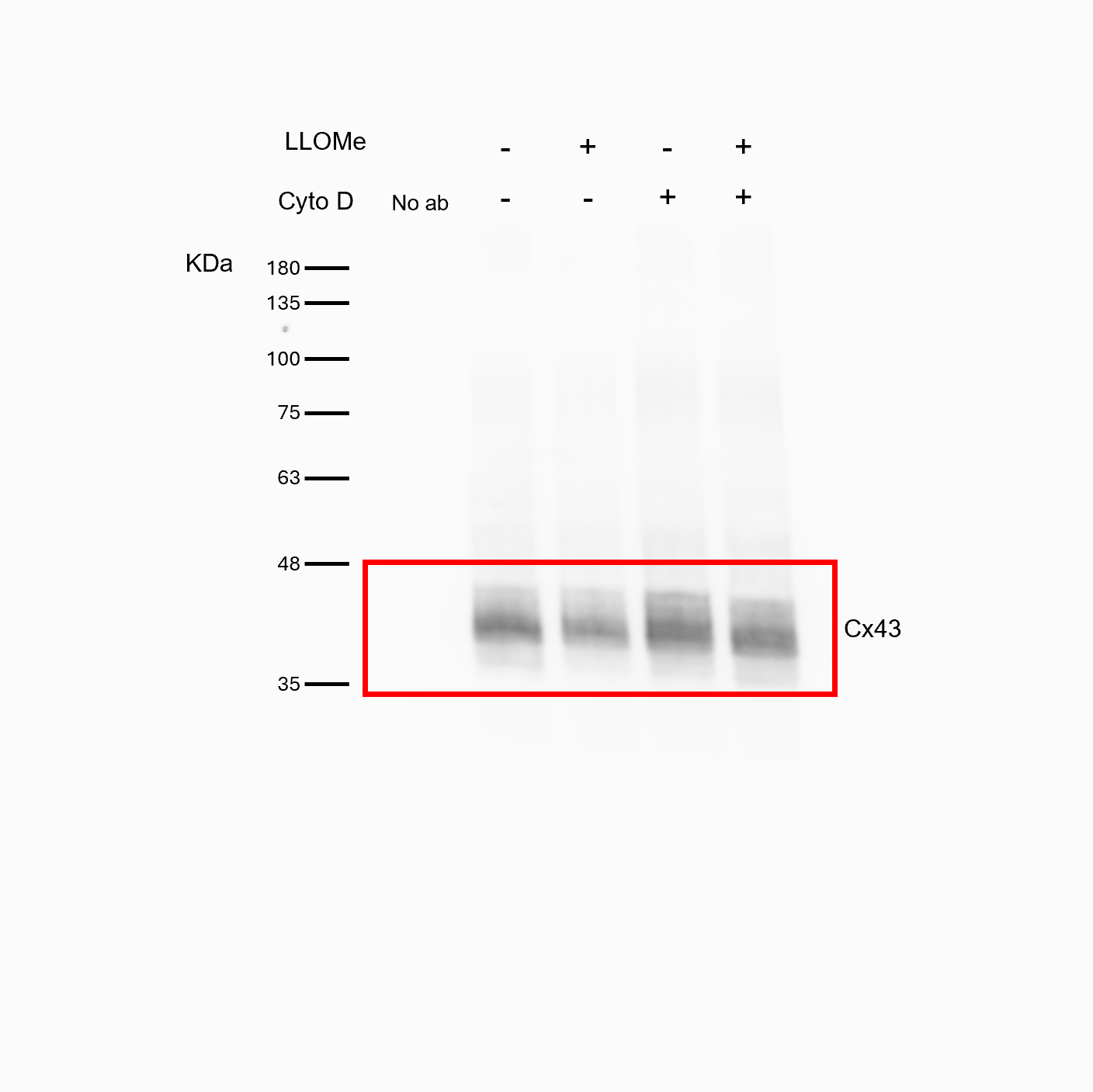

Supplement: Supplementary file 10 — Source data Fig. 6 [file 44318_2024_177_MOESM10_ESM.zip › Figure 6/6H/IP Cx43/Western Cx43 IP.tif]

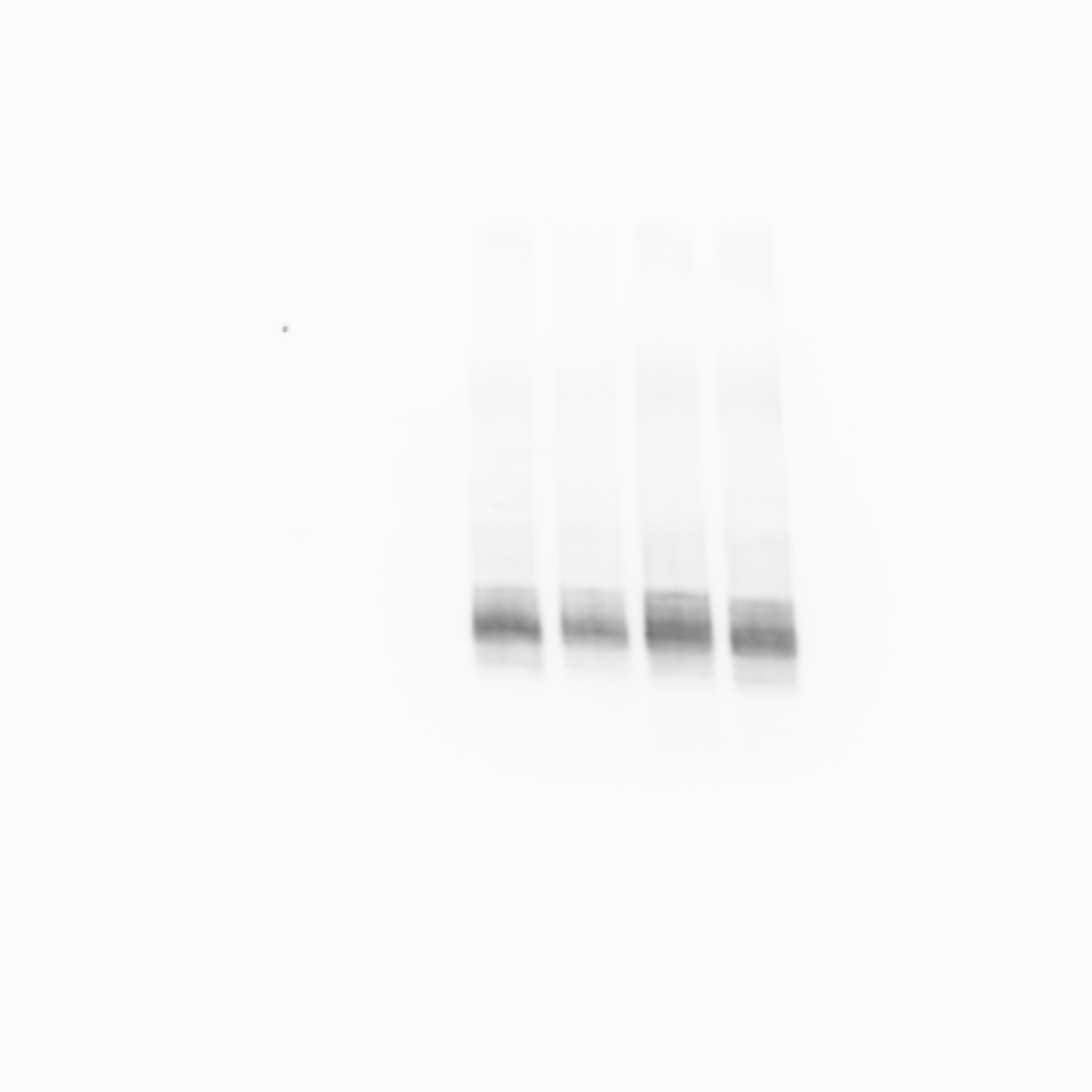

Supplement: Supplementary file 10 — Source data Fig. 6 [file 44318_2024_177_MOESM10_ESM.zip › Figure 6/6H/IP Cx43/Western Cx43 IP 20211103_152318_Ch.tif]

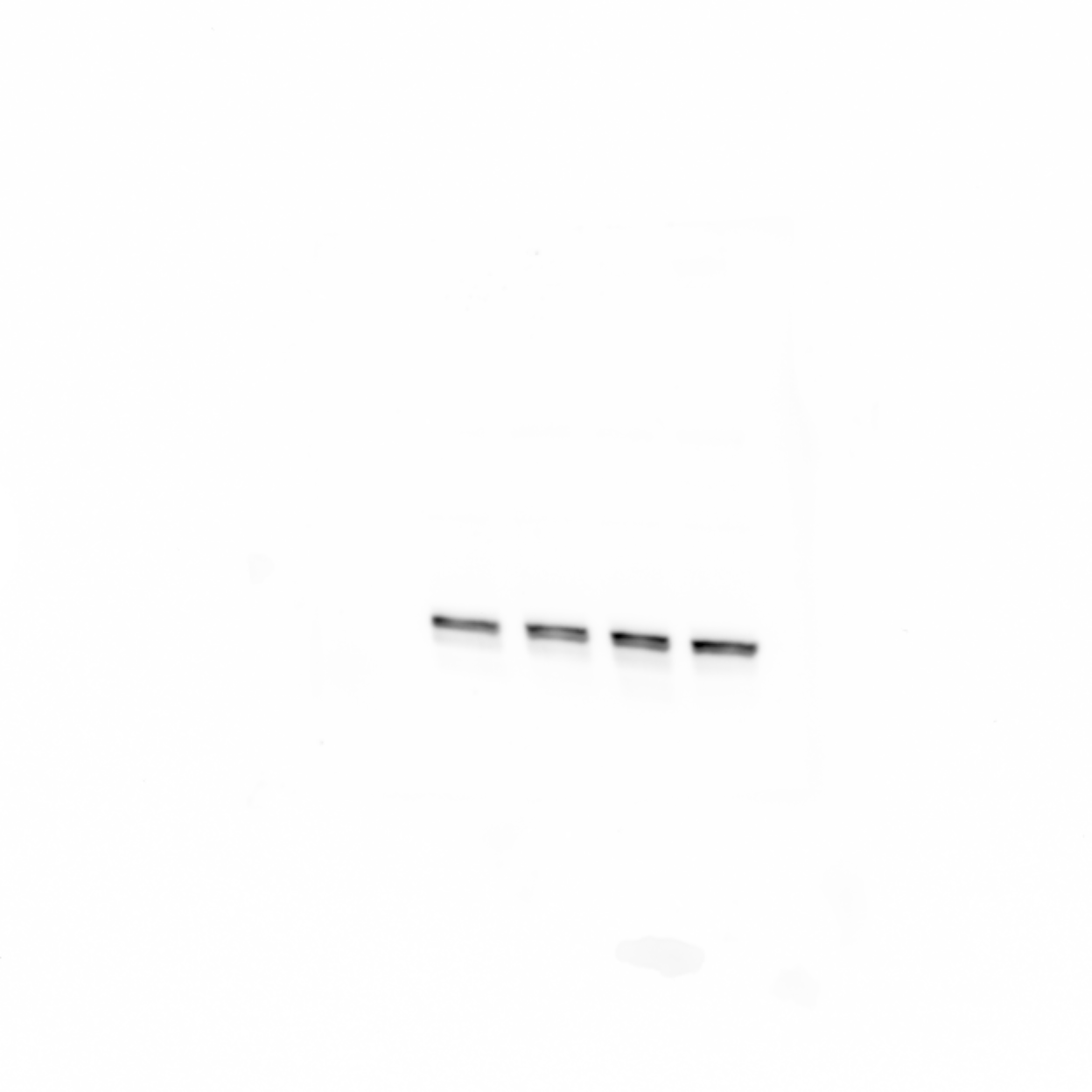

Supplement: Supplementary file 10 — Source data Fig. 6 [file 44318_2024_177_MOESM10_ESM.zip › Figure 6/6H/Input Arp2/Western Arp2 Input 20211029_133413_Ch.tif]

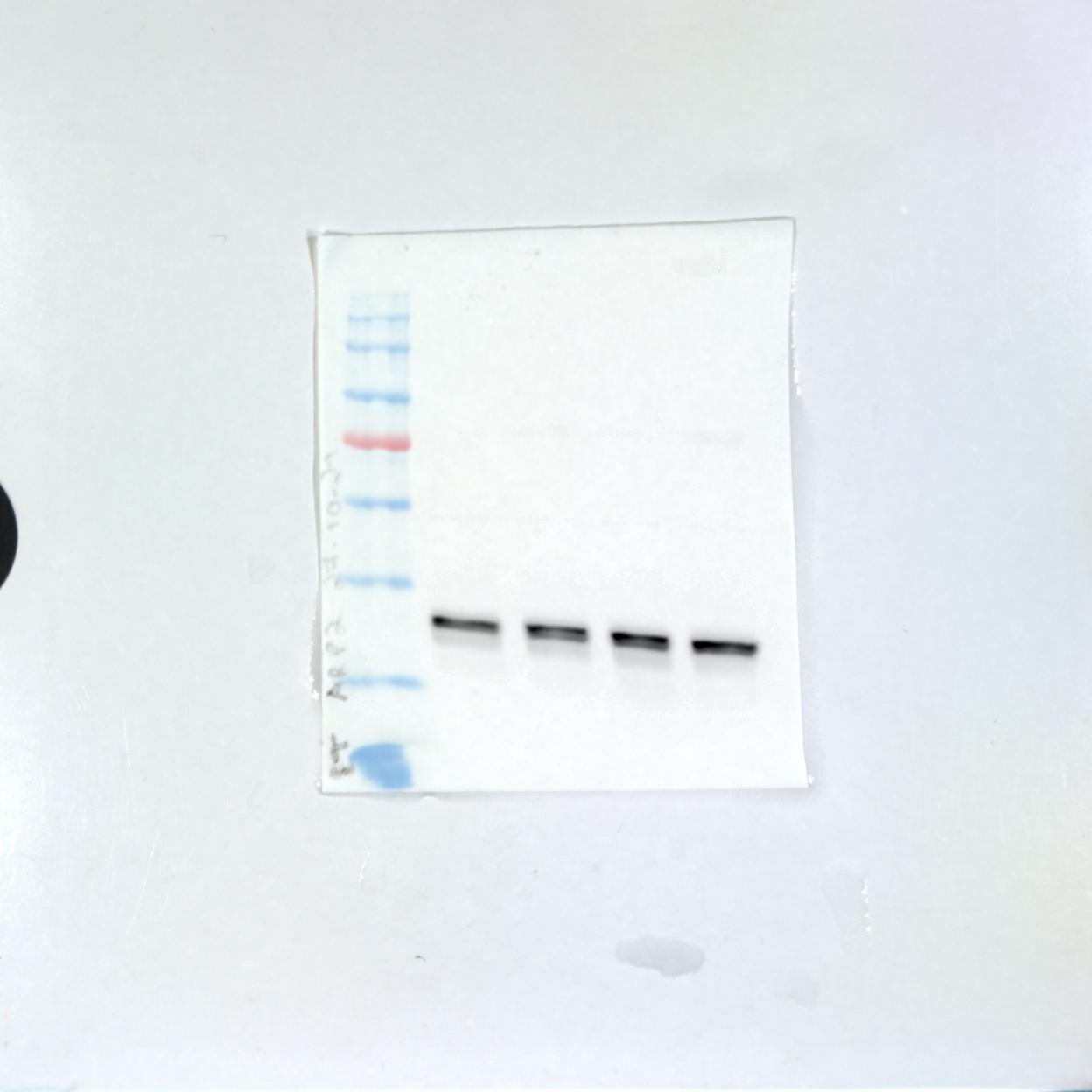

Supplement: Supplementary file 10 — Source data Fig. 6 [file 44318_2024_177_MOESM10_ESM.zip › Figure 6/6H/Input Arp2/Western Arp2 Input 20211029_133413_Ch+Marker.jpg]

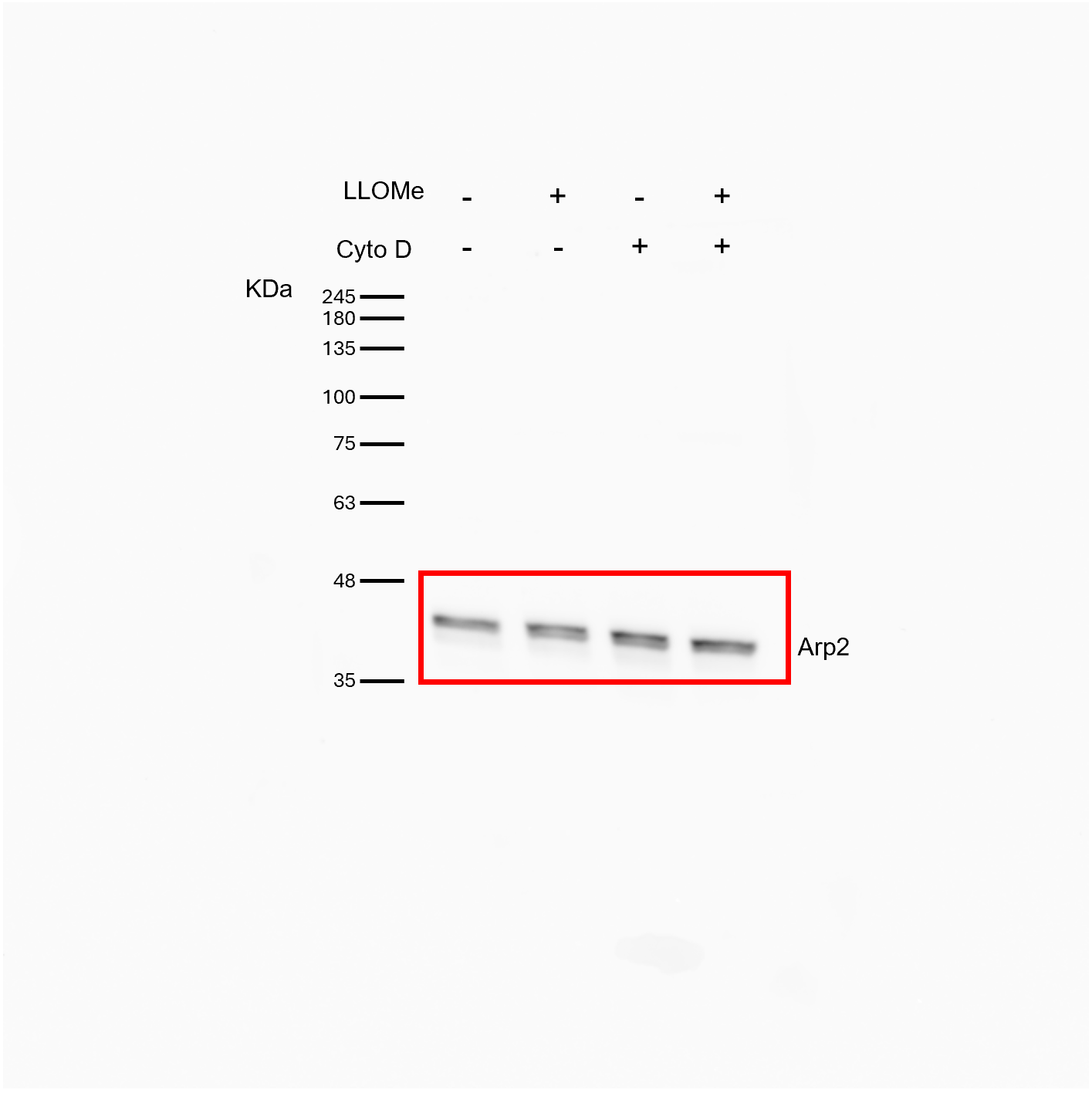

Supplement: Supplementary file 10 — Source data Fig. 6 [file 44318_2024_177_MOESM10_ESM.zip › Figure 6/6H/Input Arp2/Western Arp2 Input.tif]

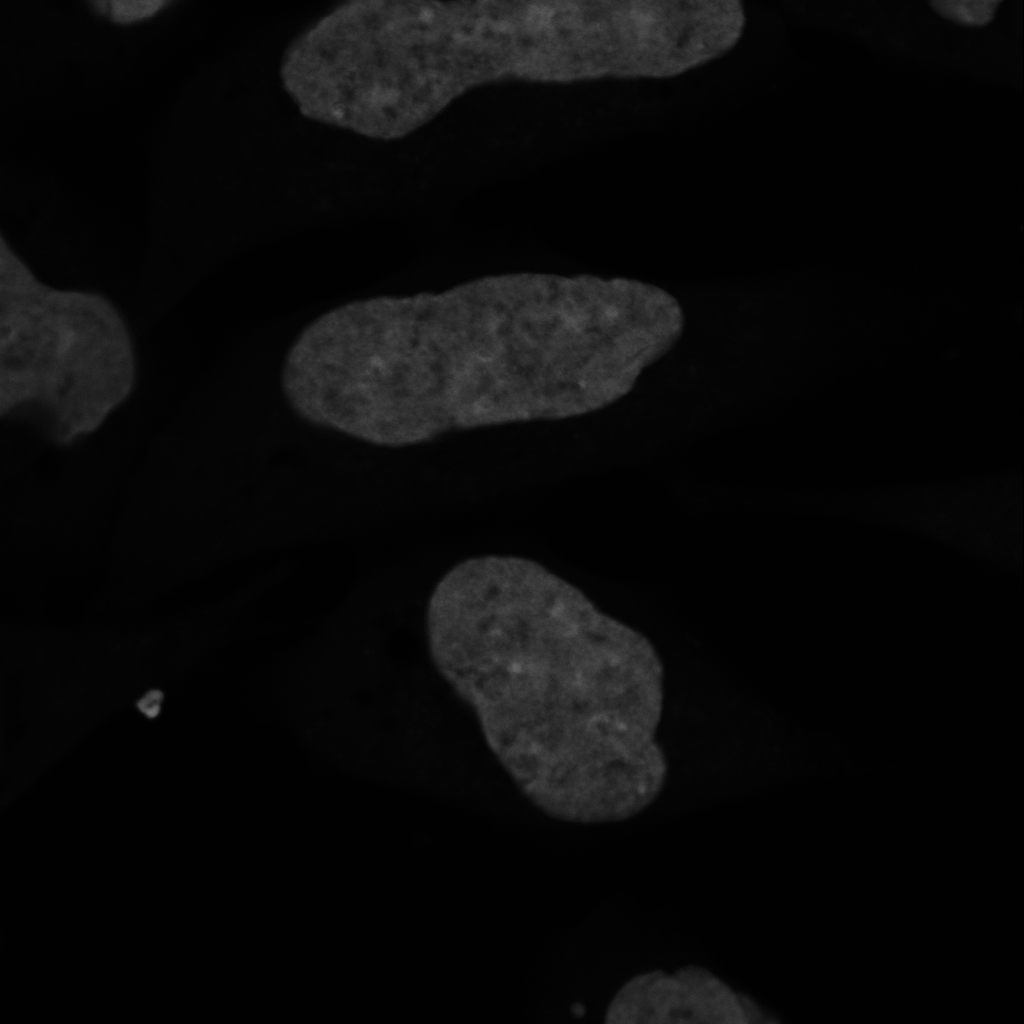

Supplement: Supplementary file 11 — Source data Fig. 7 [file 44318_2024_177_MOESM11_ESM.zip › Figure 7/7G/Confocal Fluorescence Microscopy HEK293Cx43+ siCt Dapi Cx43 488 Phalloidin 564 LAMP1 647.tif]

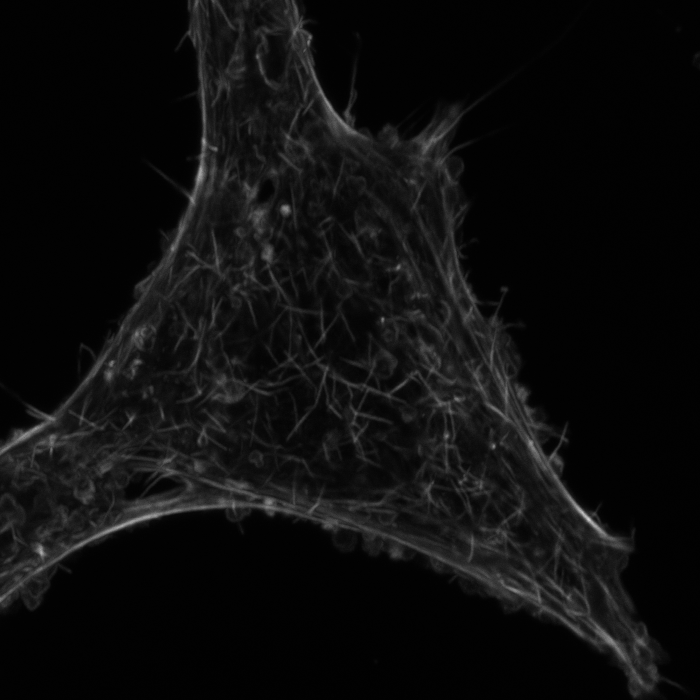

Supplement: Supplementary file 11 — Source data Fig. 7 [file 44318_2024_177_MOESM11_ESM.zip › Figure 7/7A/Confocal Fluorescence Microscopy HEK293Cx43+ siArp2 LLOMe phalloidin.tif]
