# Supplementary material for: Connexin43 promotes exocytosis of damaged lysosomes through actin remodelling
Source: EMBO J. 2024 Jul 23;43(17):3627–49. doi: 10.1038/s44318-024-00177-3 (PMC11377567; doi:10.1038/s44318-024-00177-3)
Supplement: Supplementary file 10 — Source data Fig. 6 [file 44318_2024_177_MOESM10_ESM.zip › Figure 6/6H/IP Arp2/IP Arp2 README.docx]

To obtain the “Arp2 IP” image, the upper and lower parts of the membrane were covered so the Arp2 signal could be more readily visible.

Source images of the same membrane without the covers are also provided.
